# The impact of urinary incontinence on falls: A systematic review and meta-analysis

**DOI:** 10.1371/journal.pone.0251711

**Published:** 2021-05-19

**Authors:** Shinje Moon, Hye Soo Chung, Yoon Jung Kim, Sung Jin Kim, Ohseong Kwon, Young Goo Lee, Jae Myung Yu, Sung Tae Cho

**Affiliations:** 1 Division of Endocrinology and Metabolism, Department of Internal Medicine, Hallym University Kangnam Sacred Heart Hospital, Hallym University College of Medicine, Seoul, South Korea; 2 Department of Urology, Hallym University Kangnam Sacred Heart Hospital, Hallym University College of Medicine, Seoul, South Korea; University Medical Center Utrecht, NETHERLANDS

## Abstract

**Objective:**

Previous studies on the association between urinary incontinence (UI) and falls have reported conflicting results. We, therefore, aimed to evaluate and clarify this association through a systematic review and meta-analysis of relevant studies.

**Methods:**

We performed a literature search for relevant studies in databases including PubMed and EMBASE from inception up to December 13, 2020, using several search terms related to UI and falls. Based on the data reported in these studies, we calculated the pooled odds ratios (ORs) for falls and the corresponding 95% confidence intervals (CIs) using the Mantel–Haenszel method.

**Results:**

This meta-analysis included 38 articles and a total of 230,129 participants. UI was significantly associated with falls (OR, 1.62; 95% CI, 1.45–1.83). Subgroup analyses based on the age and sex of the participants revealed a significant association between UI and falls in older (≥65 years) participants (OR, 1.59; 95% CI, 1.31–1.93), and in both men (OR, 1.88; 95% CI, 1.57–2.25) and women (OR, 1.41; 95% CI, 1.29–1.54). Subgroup analysis based on the definition of falls revealed a significant association between UI and falls (≥1 fall event) (OR, 1.61; 95% CI, 1.42–1.82) and recurrent falls (≥2 fall events) (OR, 1.63; 95% CI, 1.49–1.78). According to the UI type, a significant association between UI and falls was observed in patients with urgency UI (OR, 1.76; 95% CI, 1.15–1.70) and those with stress UI (OR, 1.73; 95% CI, 1.39–2.15).

**Conclusions:**

This meta-analysis, which was based on evidence from a review of the published literature, clearly demonstrated that UI is an important risk factor for falls in both general and older populations.

## Introduction

The proportion of adults aged ≥65 years is increasing more rapidly than that of people in other age groups because of the global increase in life expectancy. However, this increase in life expectancy also increases the risk of geriatric syndromes, which are defined as the set of multifactorial conditions affecting older adults who are vulnerable to the changing circumstances [1]. Inouye et al. reported a high prevalence of five geriatric syndromes, namely, falls, incontinence, pressure ulcers, delirium, and functional decline, which are associated with high morbidity and poor quality of life [1].

Of these geriatric syndromes, falls represent one of the most important and increasing public health problems affecting older adults because these events often require medical attention. The World Health Organization (WHO) defines falls as “events that result in a person coming to rest inadvertently on the ground or floor or other lower-level.” These events are often recurrent, and approximately half of the affected individuals experience another fall within 1 year [2]. According to the WHO, 28–35% of people older than 65 years of age fall each year, and this prevalence increases with age [3]. Another study determined that more than 30% of older (>65 years) home-dwelling individuals fall at least once per year [4]. Consequently, a substantial proportion of these individuals develop serious injuries, pain, depression, and other comorbidities. Even a slight fall can cause a fracture, which increases the risk of institutionalization and the associated economic burden. Falls also instill a source of fear in caregivers and negatively affect the healthcare systems [3]. In summary, falls result in negative health outcomes and limit the quality of life of older individuals, and strategies to prevent this geriatric syndrome should be established.

Assessing the association between falls and other geriatric syndromes [1] is clinically important in preventing falls. This syndrome is highly prevalent in the general population and affects men and women of all ages. Of the other geriatric syndromes, urinary incontinence (UI) is more common in women than in men; however, and the prevalence increases with age. Current estimates suggest that approximately 20 million women and 6 million men in the United States experience UI during their lives. This condition has been shown to affect 11–34% of men and 13–50% of women older than 60 years and 43–80% of all older nursing home residents [5]. UI is associated with not only a decreased quality of life but also a longer hospital stay and a reduced chance of hospital discharge [5]. However, many patients, particularly older individuals, avoid or do not receive treatment for UI due to the social stigma attached to the condition.

Although several epidemiological studies have evaluated the effects of UI on falls, the results of analyses based on age, sex, and the definition of falls have been inconclusive. Although some studies reported that UI is positively associated with falls [6–8], others indicated no association [9–11]. Hence, a meta-analysis was warranted to clarify our understanding of the role of UI in falls. We, therefore, performed a meta-analysis to provide evidence and determine the effect of UI on the risk of falls based on a comprehensive investigation of the literature. Furthermore, we conducted subgroup analyses based on patients’ mean age, sex, the definition of falls, and type of UI.

## Methods

### Search strategy

A literature search was conducted in adherence to the principles outlined by the Preferred Reporting Items for Systematic Reviews and Meta-Analyses—PRISMA (S1 Table). The study protocol was registered in PROSPERO (CRD42021225038). Two independent investigators (S.M. and S.T.C.) searched citation databases (PubMed, EMBASE, and Web of Science) for relevant studies. The search terms were a combination of “urinary incontinence” and “fall.” The search was limited to original articles written in English and published between database inception and December 13, 2020 (S2 Table).

### Study selection

The inclusion criteria were as follows: 1) population: studies with participants aged ≥ 50 years or mean age ≥ 60 years; 2) exposure: the presence of UI; 3) comparators: participants without UI; 4) outcomes: incidence of falls; and 5) study design: case-control or cohort studies.

The exclusion criteria were as follows: 1) articles published as experimental studies, containing only abstracts, and published as non-original articles, including expert opinions or reviews; 2) studies that enrolled young adults aged <40 years; 3) observational studies without a control group.

### Data extraction

Data of the following variables were extracted independently by two investigators using the same criteria: name of the first author, year of publication, country, demographic characteristics of the participants, mean age of the participants, number of study participants, number of cases of falls, and odds ratios (OR) with 95% confidence intervals (CI).

### Risk of bias assessment

We used the Risk Of Bias In Non-randomized Studies—of Exposures (ROBINS-E), a modified form of ROBINS—of Interventions (ROBINS-I), to assess the methodological quality of the included studies [12, 13]. Discrepancies were resolved by discussion with a third investigator (J.M.Y).

### Data analyses and statistical methods

The overall ORs and 95% CIs of all studies were computed using the Mantel–Haenszel method. Heterogeneity among the studies was tested using the Higgins I^2^ statistic, where an I^2^ of ≥50% indicated heterogeneity. We computed the ORs using the random-effects model. Publication bias was calculated using a funnel plot and Egger’s test. Sensitivity analysis was also performed.

### Subgroup analysis

All analyses were conducted using the Comprehensive Meta-Analysis software version 3 (Biostat, Englewood, NJ, USA).

## Results

### Study characteristics

**Fig 1:**
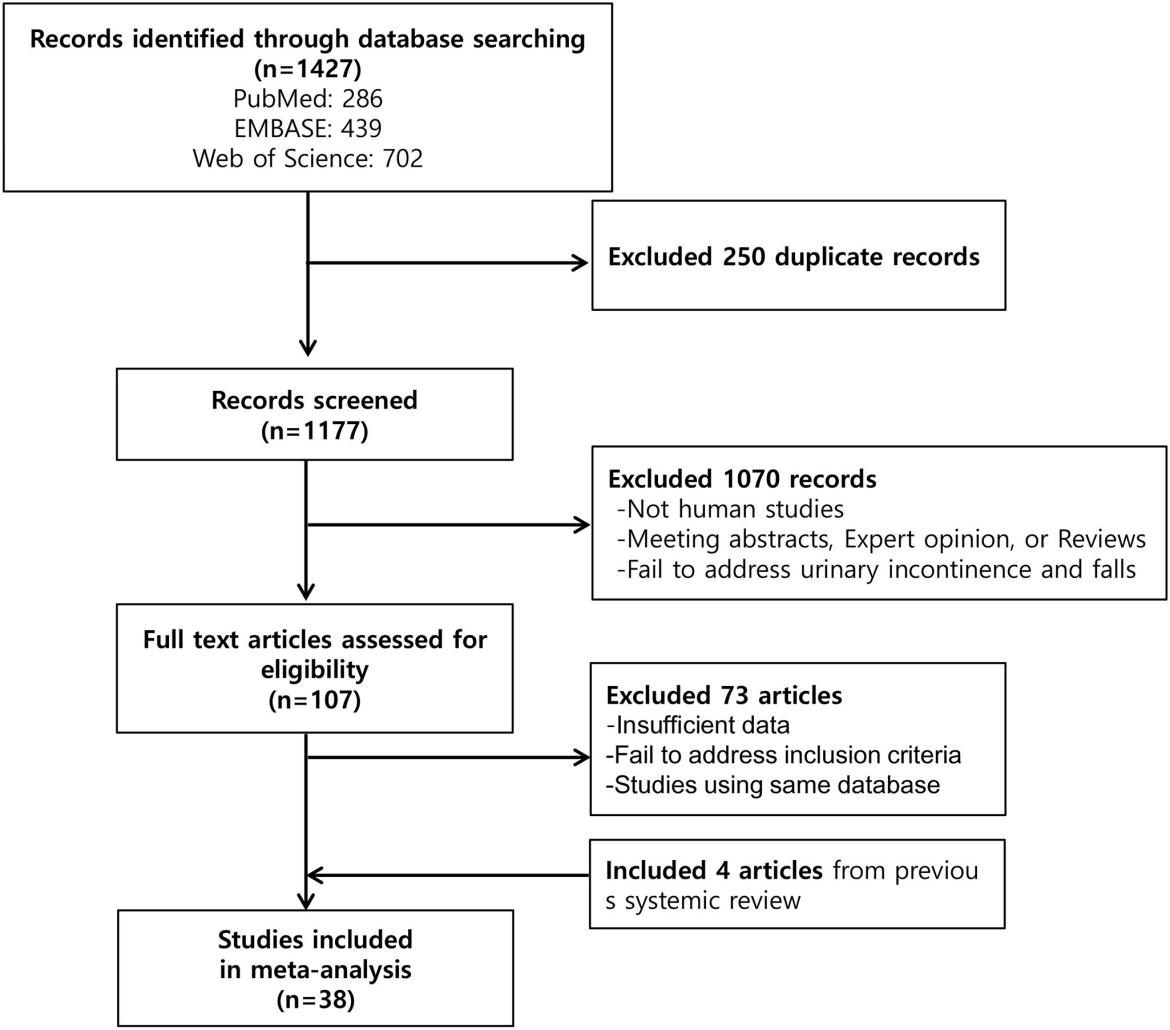
Schematic diagram of the search strategy.

In total, 1,427 studies were identified from the literature search (PubMed: 286, EMBASE: 439, and Web of Science: 702). After excluding 250 duplicate studies, we reviewed the remaining studies. Next, 1,177 studies were excluded during primary screening. After reviewing the texts of 107 articles, we excluded 73 studies, resulting in the inclusion of 34 articles [2, 7–11, 14–40]. In addition, we found four eligible studies from a previous review [41]. Finally, a total of 38 studies with 230,129 participants were included in this meta-analysis (Fig 1).

The main characteristics of the studies are summarized in Table 1 [2, 7–11, 14–40, 42–46]. The meta-analysis revealed that, overall, 27.6% of participants (n = 63,618) experienced falls. The definitions of falls varied across the reviewed studies. Twenty-nine studies defined a fall as ≥1 fall event [7–9, 11, 17, 18, 20, 22, 23, 26–40, 42–46], four studies defined a fall as ≥2 fall events [14, 15, 21, 25], and five studies defined a fall as ≥1 fall event and recurrent falls as ≥2 fall events [2, 10, 16, 19, 24].

**Table 1:** Summary of the 38 studies included in the present meta-analysis.

Study [Reference]	Country	Source of sample	Population characteristics	No. of total participants	Definition of falls/ No. of participants with falls	Definition and type of UI/ No. of participants with UI	Relative risk (95% CI)	Risk of bias
Tinetti ME et al. 1995 [14]	USA	Community dwelling adults, aged 72 years and older	Mean age: 79.7 Women:73%	927	At least two falls in 1 year	At least one UI / week in 1 year	Crude OR: 1.9 (95% CI, 1.2–2.9)	Critical
					96	146		
Luukinen H et al. 1996 [15]	Finland	Community dwelling adults, aged 70 years and older	Mean age: 76.1	1,016	At least two falls in 1 year	UI during the past 2 years	Adjusted ^a^ OR: 1.70 (95% CI, 1.03–2.89)	Serious
			Men: 396 Women: 620		88	158		
Johansson C et al. 1996 [9]	Sweden	Community dwelling women, aged 85 year old	Mean age: 85	658	At least one falls	UI monthly, weekly, several/week, daily, several/day.	Crude OR: 1.00 (95% CI, 0.75–1.33)	Critical
			Women:100%		286	urge type (46%),		
						stress (21%), mixed (33%)		
						384		
Brown JS et al. 2000 [7]	USA	Community-dwelling women, aged 65 years and older	Mean age: 78.5	6,049	At least one falls in 1 year	UI during the past 1 year.	Adjusted ^b^ OR:	Moderate
			Women:100%		1,927	At least one UI:2,818 (46.6%),	-Stress type: 1.06 (95% CI, 0.95–1.19)	
						At least weekly urge type: 1,493 (24.7%), At least weekly stress type: 1,137(18.8%), Both type: 708(11.7%).	-Urge type: 1.26 (95% CI, 1.14–1.40)	
Tromp AM et al. 2001 [16]	Netherlands	Community-dwelling adults, aged 65 years and older	Mean age: 72.6	1,469	At least one falls in 1 year	Self- reported UI 24%	Adjusted ^c^ OR: 1.6 (95% CI, 1.2–2.1)	Serious
			Men: 705 Women: 764		464			
							Recurrent fall: Adjusted OR: 1.7 (95% CI, 1.2–2.5)	
de Rekeneire N et al. 2003 [17]	USA	Community-dwelling adults, aged 70 to 79 years	Age (70–79) Men: 1,447		At least one falls in 1 year	Self- reported UI1,175	Adjusted ^d^ OR:	Serious
			Women: 1,515	2,962	652		- Men 1.5 (95% CI, 1.1–2.0)	
							- Women: 1.5 (95% CI, 1.2–1.9)	
Takazawa K et al. 2005 [10]	Japan	Women in a day care service at geriatric health facility	Median age: 81	118	At least one falls in 1 year	At least once a week during the past 1 year	Crude OR: 1.12 (95% CI, 0.54–2.32)	Critical
			Women:100%		56			
						Stress type: 25 (49.0%), Urge type: 46(90.2%) 52		
Teo JS et al. 2006 [18]	Australia	Community-dwelling women	Mean age: 79.1	782	At least one falls in 1 year	Self- reported UI (regardless of amount and frequency)	Adjusted ^e^ OR: -Stress type: 1.06 (95% CI, 0.77–1.45)	Serious
			Women:100%		275			
							-Urge type: 1.96 (95% CI, 1.45–2.65)	
						Stress type: 69.4% (pure 36.8%)		
						Urge type: 36.3% (pure 3.7%), both type: 32.6%.		
						73.1%		
Hasegawa J et al. 2010 [19]	Japan	Disabled older people who were admitted to facilities	Mean age: 82.5	1,082	At least one falls	UI events during placement 180	Adjusted ^f^ OR: 2.14 (95% CI, 1.03–2.89)	Serious
					264			
			Men: 327					
			Women: 755					
Foley AL et al. 2012 [8]	UK	Community-dwelling adults aged 70 years or Over	Median age: 76	5,474	At least one falls in 1 year	Self- reported UI	Crude OR:	Critical
			Men: 2,245		1,813	Stress type: 16.5%, urge type: 24.9%	-Stress type: 3.56 (95% CI, 3.06–4.15)	
			Women: 2,917		26.7%	-Urge type: 2.19 (95% CI, 1.92–2.49)		
Allain TJ et al. 2014 [20]	Malawi	Community-dwelling adults aged 60 years or Over	Mean age: 72	98	At least one falls in 1 year	Self- reported UI	Crude OR: 3.27 (95% CI, 1.26–8.50)	Critical
			Men: 29					
			Women: 69		40	25%		
Huang LK et al. 2015 [21]	Taiwan	Community-dwelling adults aged 65 years or Over	Age ≥65 years	187	At least two falls in 1 year	UI in the past 1 year and 1week.	Adjusted ^g^ OR: 1.86 (95% CI, 0.86–4.02)	Serious
			Men: 65					
			Women: 122		53	29.9%		
Kim H et al. 2015 [22]	Japan	Community-dwelling women aged 75–84 years	Mean age: 78.5	1,399	At least one falls	UI over once a week	Crude OR: 1.57 (95% CI, 1.14–2.16)	Critical
			Women:100%		269	Stress type: 29.2% (76/260), Urge type: 25.0% (65/260), and Mixed type:45.8% (119/260)		
						260		
Sakushima K et al. 2016 [11]	Japan	Ambulatory patients with Parkinson’s disease in an outpatient clinic of an academic hospital	Mean age: 71.5	97	At least one falls in 6 months	Mild: less than once a day, severe: once a day or more past 1 week.	Crude OR: 2.05 (95% CI, 0.88–4.73)	Critical
			Men: 40		44			
			Women: 57					
						Mild 27		
						Severe 17		
Schluter PJ et al. 2018 [23]	New Zealand	Community-dwelling adults aged 65 years or Over	Mean age: 82.7	67,288	At least one falls in 90 days	UI in the last 3 days	Adjusted ^h^ OR:	Moderate
						Occasional UI: less than daily, frequently UI: daily		
			Men: 25,257		27,213		-Men	
			Women: 42,032				Occasional UI 1.53 (95% CI, 1.43–1.64)	
						Men 34.3%		
						Women 42.6%		
							Frequent UI 1.69 (95% CI, 1.57–1.82)	
							-Women	
							Occasional UI	
							1.33 (95% CI, 1.26–1.39)	
							Frequent UI 1.39 (95% CI, 1.32–1.46)	
Agudelo-Botero M et al. 2018 [24]	Mexico	Community-dwelling adults aged 60 years or Over	Age ≥60 years	9,598	At least one falls in 2 years	UI during the last 2 years	Adjusted ^i^ OR: -Occasional falls	Moderate
			Men: 4,271					
					4,466 (46%, one fall 16%, recurrent falls 30%))	3,021		
			Women: 5,327				1.12 (95% CI, 0.98–1.28)	
							-Recurrent falls 1.52 (95% CI, 1.37–1.69)	
Kang J et al. 2018 [25]	Korea	Patients older than 65 who visited the geriatric clinic	Mean age: 73	404	At least two falls in 6 months	UI during the last 1 month	Crude OR: 2.07 (95% CI, 1.23–3.35)	Critical
			Men: 114					
					89	133		
			Women: 290					
Kim HJ et al. 2018 [26]	Korea	Community-dwelling adults aged 66 years or over in nationwide cohort study	Age (66–80) Men: 20,943	39,854	At least one falls in 6 months	Self- reported UI	Crude OR: 5.29 (95% CI, 4.87–5.73)	Critical
						5,703		
			Women: 18,911		2,802			
Sohn K et al. 2018 [27]	Korea	Community-dwelling women aged 65 years or over in Korean Longitudinal Study of Ageing	Age ≥65 years	2,418	At least one falls in 2 years	UI in the past 1 year	Crude OR: 1.29 (95% CI, 0.92–1.79)	Critical
			Women:100%					
					204	506		
Singh DKA et al. 2019 [28]	Malaysia	Community-dwelling adults aged 60 years or Over	Mean age: 68.9	3,901	At least one falls in 1 year	Self- reported UI	Adjusted ^j^ OR: 1.35 (95% CI, 1.07–1.69)	Serious
			Men: 1,807 Women: 2,127		804	615		
Peeters G et al. 2019 [29]	Australia, Netherlands, Great Britain Ireland	Community-dwelling adults from four cohort (ALSWH, LASA, NSHD, TILDA)	Mean age:	ALSWH: 10,641	At least one falls in 1 year	Self- reported UI	Adjusted ^a^ OR:	Serious
			-ALSWH: 55.0–63.1.			-ALSWH: 45.6–59.0%		
				LASA: 802	-ALSWH: 2,352		-ALSWH:	
						-LASA: 16.7%	1.53 (95% CI, 1.44–1.63) -LASA:	
			Women:100%-LASA: 59.7	NSHD: 2,987	-LASA: 201	-NSHD: 32.2%		
					-NSHD: 520	-TILDA: 10.3–12.6%	1.62 (95% CI, 0.95–2.78)	
			Women:51.6%	TILDA: 4663	-TILDA: 820			
							-NSHD: 1.68 (95% CI,	
			-NSHD: 53.5–63.4					
							1.22–2.31) -TILDA: 2.09 (95% CI, 1.75–2.49)	
			Women:50.9–52.2%					
			-TILDA: 56.7–58.6					
			Women:55.5–57.3					
Giraldo-Rodriguez L et al. 2019 [30]	Mexico	Community-dwelling adults aged 50 years or Over	Aged ≥ 50	13,626	At least one falls in 2 years	UI during the past 2 years	Crude OR:	Critical
							- Men: 1.42	
			Men: 5,843		5,341	-Men: 730 (12.5%)	(95% CI, 1.18–1.71)	
			Women: 7,783			Stress type:141(2.4%), urge type:317(5.4%), mixed type:272(4.7%)		
						-Women: 2,155		
						(27.7%)		
							- Women:	
							1.22 (95% CI, 1.06–1.39)	

						Stress type:731(9.4%), urge		
						type:488(6.3%), mixed type:936(12%)		
Huang MH et al. 2019 [31]	USA	Men aged 65 years or over who had prostate cancer or breast cancer	74.5(men)	1097	At least one falls in 1 years	UI during the past 6 months	- Men Adjusted ^k^	Serious
			75.1(women)					
			Men: 660		231	285(men)	OR:	
			Women: 437			219 (women)	1.69 (95% CI, 1.08–2.65)	
							-Women	
							Crude OR: 2.27 (0.89–5.80)	
Abbs E et al. 2020 [32]	USA	Homeless adults aged 50 years or Over	Median age: 58	350	At least one falls in the past 6 months	UI during the past 6 months 167	Adjusted ^l^ OR: 1.40 (95% CI, 1.07–1.81)	Moderate
			Men: 270					
			Women: 80		118			
Abell JG et al. 2020 [33]	UK	Community-dwelling adults aged 60 years or Over	Mean age: 69.6	3,783	At least one falls in 1 year	UI during the past 12 months	Adjusted ^m^ HR:: 1.49 (95% CI, 1.14–1.95)	Moderate
			Men: 1,791		315	574		
			Women: 1,992					
Britting S et al. 2020 [34]	Austria	Community-dwelling adults aged 75 years or over from SCOPE cohort	Median age: 79.5	2,256	At least one falls in 1 year	UI during the last 1 month	Adjusted ^n^ OR: 1.33 (95% CI, 1.09–1.63)	Moderate
	Germany							
	Israel		Men: 1,000		746	653		
	Italy		Women: 1,256					
	Netherlands							
	Poland							
	Spain							
Dokuzlar O et al. 2020 a [35]	Turkey	Women aged 65 years or over	Mean age: 74.4	682	At least one falls in 1 year	UI during the past 12 months	Adjusted ^o^ OR: 1.61 (p value: 0.006)	Serious
			Women:100%		215	55.4%		
Dokuzlar O et al. 2020 b [36]	Turkey	Men aged 65 years or over	Mean age: 75.0	334	At least one falls in 1 year	UI during the past 12 months	Adjusted ^o^ OR: 2.468 (p-value: 0.001)	Serious
			Men:100%		85	33.2%		
Lee K et al. 2020 [37]	USA	Community-dwelling adults aged 65 years or over	Mean age: 70.4	17,712	At least one falls in 2 year	UI during the past 12 months	Adjusted ^p^ OR: 1.96 (95% CI, 1.59–2.40)	Serious
					4,779	3,340		
			Men: 7,626					
			Women: 10,086					
Magnuszewski L et al. 2020 [38]	Poland	Patients admitted to the department of geriatrics	Mean age: 85	358	At least one falls in 1 year	Self- reported UI	Adjusted ^q^ OR: 1.37 (95% CI, 0.75–2.49)	Serious
			Men:80			146		
			Women: 278		157			
Moon S et al. 2020 [2]	Korea	Community-dwelling women aged 65 years or over	Mean age: 74.5	6,134	At least one falls in 1 year	Self- reported UI	Adjusted ^r^ OR: 1.33(95% CI, 1.00−1.76)	Moderate
						281		
			Women:100%		1,152			
Savas S et al. 2020 [39]	Turkey	Community-dwelling adult	Mean age: 65	1176	At least one falls in 1 year	Self- reported UI	Crude OR:	Critical
			Men:592			346	1.21 (95% CI, 0.79–1.87)	
			Women: 584		276			
Tsai YJ et al. 2020 [40]	Taiwan	Community-dwelling adults aged 65 years or over (NHIS 2005, 2009, 2013)	Men:4,142	8,822	At least one falls in 1 year	Self- reported UI	Adjusted ^s^ OR: 1.09 (0.80–1.49), 1.29 (0.90–1.84), 1.42 (1.04–1.94)	Serious
			Women: 4,680			1,573		
					1,672			
Cesari M et al.2002 [42]	Italy	Community-dwelling adults admitted to national home care program	Mean age: 77.2	5,570	At least one falls in 90 days	Self- reported UI	Adjusted ^t^ OR: 1.06 (0.93–1.20),	Serious
					1,997	1,744		
				Men: 2,290				
				Women: 3,280				
Hedman AM et al. 2013 [43]	Sweden	Community-dwelling adults aged 75 years or over	Median age: 81	1,243	At least one falls in 1 year	Self- reported UI 1,139	Adjusted ^u^ OR: 1.53 (1.23–1.91),	Serious
			Men: 471		434	425(men)		
			Women: 772			714(women)	- Men: 1.67 (1.13–2.47),	
							- Women: 1.53 (1.16–2.00)	
Moreira MD et al. 2007 [46]	Brazil	Community-dwelling adults aged 60 years or over	Mean age: 79	490	At least one falls in 1 year	Self- reported UI 86	p <0,025	Critical
			Men: 116					
					137			
			Women: 374					
Stenhagen M et al. 2013 [44]	Sweden	Community-dwelling adults aged 60 years or over	Men: 264	1,736	At least one falls in 6 months	Self- reported UI	Crude OR: 1.89 (1.38–2.58)	Serious
			Women: 394 (3-year follow up)	555 (3-year follow up)		267		
					106(3-year follow up)			
				1,542(6-year follow up)			With UI: 267 (67 with falls)	
			Men: 784		205(6-year follow up)			
			Women: 963 (6-year follow up)					
							Without UI: 1453 (219 with falls)	
							Adjusted ^a^ OR: 1.31 (0.94–1.82)	
van Helden S et al. 2007 [45]	Netherland	Patients older than 50 who visited the geriatric clinic	Mean age: 67.1	277	At least one falls in 3 months	Self- reported UI	Crude OR: 2.07 (0.98–4.41)	Critical
			Men: 77		42	50		
			Women: 200					

OR, odds ratios; HR, hazard ratios; UI, urinary incontinence; CI, confidence intervals; ALSWH, The Australian Longitudinal Study on Women’s Health; LASA, The Longitudinal Ageing Study Amsterdam; NSHD, The MRC National Survey of Health and Development; TILDA, The Irish Longitudinal Study on Ageing.

^a^ adjusted for age and sex,

^b^ adjusted for age, living situation, overall frailty, number of falls in the previous year, whether she walked for exercise, alcohol and caffeine consumption, medical history, medication use, grip strength, gait speed, whether she used her arms to stand from chair, and performance of 10-second tandem balance.

^c^ adjusted for age, gender, educational level, urbanization level, chronic diseases, physical function, level of activity and mobility, previous falls, fear of falling

^d^ adjusted for age, race, study site, and body mass index.

^e^ adjusted for age, central nervous system drug and cardiovascular system drugs.

^f^ adjusted for age, gender, physical function, behavioral symptom, and medication use.

^g^ adjusted for gender, depressive mood, and activities involving lower limb.

^h^ adjusted for: age, ethnicity, marital status, living arrangements, body mass index, cognitive performance, dementia, congestive heart failure, Chronic obstructive pulmonary disease, depression, diabetes mellitus, alcohol consumption, smoking status, hearing status, vision status, fatigue, mobility, stability, dizziness, wandering, season, bisphosphonates, vitamin D, and calcium.

^i^ adjusted for sociodemographic, medical and functional covariables.

^j^ adjusted for age, sex, educational level and ethnicity.

^k^ adjusted for age at prostate cancer diagnosis, time since cancer diagnosis, history of falls, marital status, physical summary score of Veterans RAND 12-Item Health Survey.

^l^ adjusted for age, sex, race, stroke, Activities of Daily Living (ADL) impairment, use of an assistive device., marijuana use, opioid use, history of physical assault, any nights spent in unsheltered settings.

^m^ adjusted for age, sex, chronic conditions (coronary heart disease, diabetes, Stroke, Arthritis, Osteoporosis, Parkinson’s Disease), BMI, Smoking status, Alcohol consumption, The Short Physical Performance Battery (SPPB), and history of severe fall.

^n^ adjusted for age, gender, geriatric depression score (GDS), chronic kidney disease (CKD), instrumental activities of daily living (IADL) score and Euro-Qol 5D Score.

^o^ adjusted for age, education level, and living environment.

^p^ adjusted for age, sex, race/ethnicity, and spouse/partner status.

^q^ adjusted for age, multimorbidity, chronic diseases (cardiac heart failure, peripheral arterial disease, history of stroke/ transient ischemic attack, Parkinson’s disease, and chronic osteoarthritis, Performance Oriented Mobility Assessment, Barthel Index, IADL score, gait speed, Clinical Frailty Scale, Mini Nutritional Assessment Short Form, albumin value, vitamin B12 level and taking certain medications (quetiapine, vitamin D, diuretics, benzodiazepines and selective serotonin reuptake inhibitor).

^r^ adjusted for age, smoking status, alcohol consumption, body mass index, hypertension, dyslipidemia, and diabetes mellitus, cognitive impairment, ADL and IADL disability, visual and hearing impairment, and lower limb weakness.

^s^ adjusted for age, sex, developing difficulty in performing ADLs or IADLs, use of sleeping pills, vision, comorbidities, depressive symptoms, and frequency of exercise.

^t^ adjusted for age, gender, activities of daily living impairment, foot problems, gait problems, fear of falling, visual impairment, wandering, depression, parkinsonism, and environmental hazards.

^u^ Poor self-rated health, Pain in neck and shoulders, Back pain, sciatica or hip pain, Pain in hands, elbows, legs or knees, Headache or migraine, Anxiety, Tiredness, Sleeping disorders, Tinnitus, Recurring stomach problems, Overweight/Underweight.

**Fig 2:**
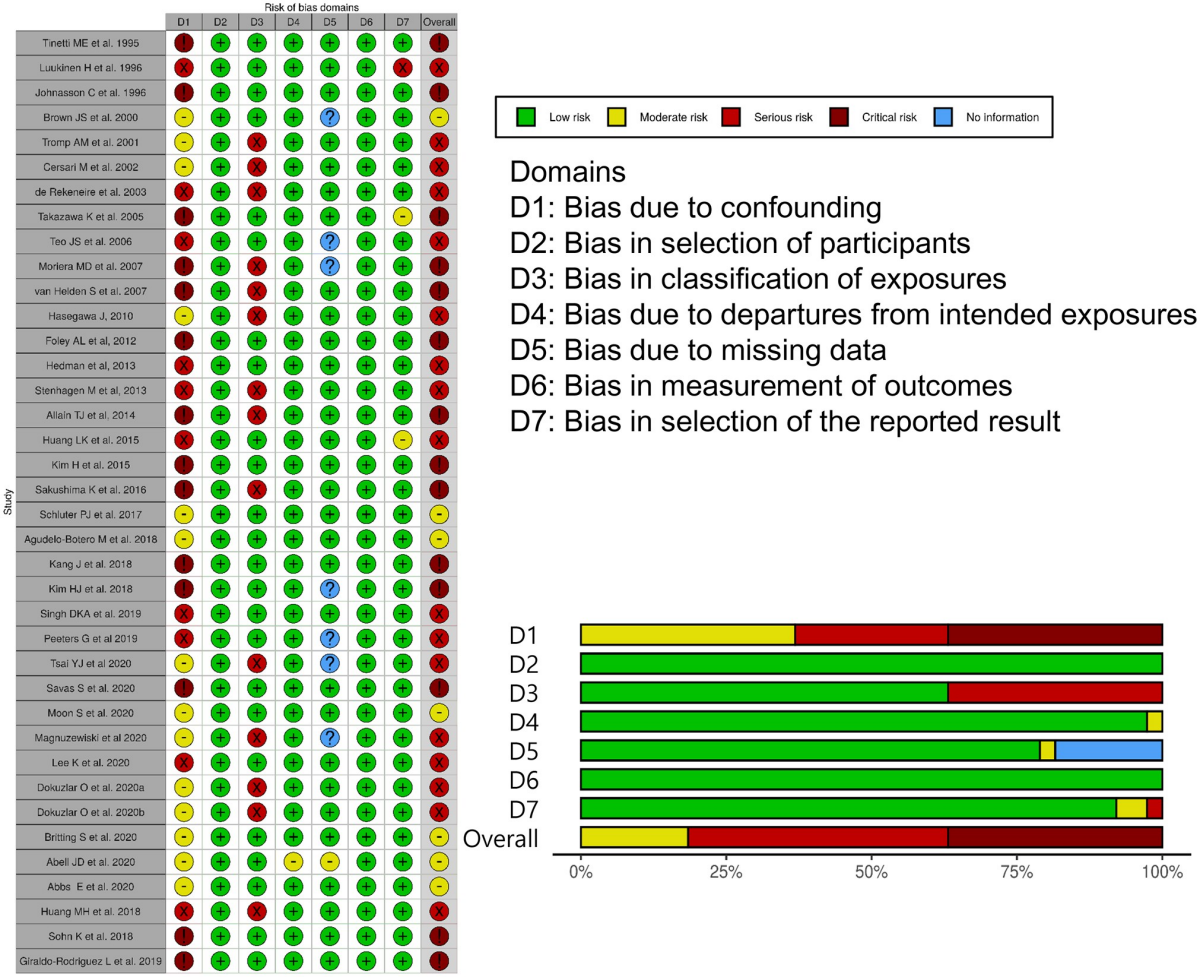
Quality assessment of the risk of bias in the 33 studies included in this meta-analysis.

Fig 2 summarizes the quality assessment results of the studies and shows that the major source of bias in the studies bias was the lack of adjustment for potential confounders. Among the 38 studies, 14 studies did not adjust for confounding factors and were classified as studies with a critical risk of bias [8–11, 14, 20, 22, 25–27, 30, 39, 45, 46]. Seventeen studies had a serious risk of bias since more than one critically important confounding factor, namely age, sex, and physical function, was not appropriately adjusted or UI was not properly defined [15–19, 21, 28, 29, 31, 35–38, 40, 42–44]. Seven studies, which were appropriately adjusted for confounding factors, had a moderate risk of bias [2, 7, 23, 24, 32–34].

### Impact of UI on falls

**Fig 3:**
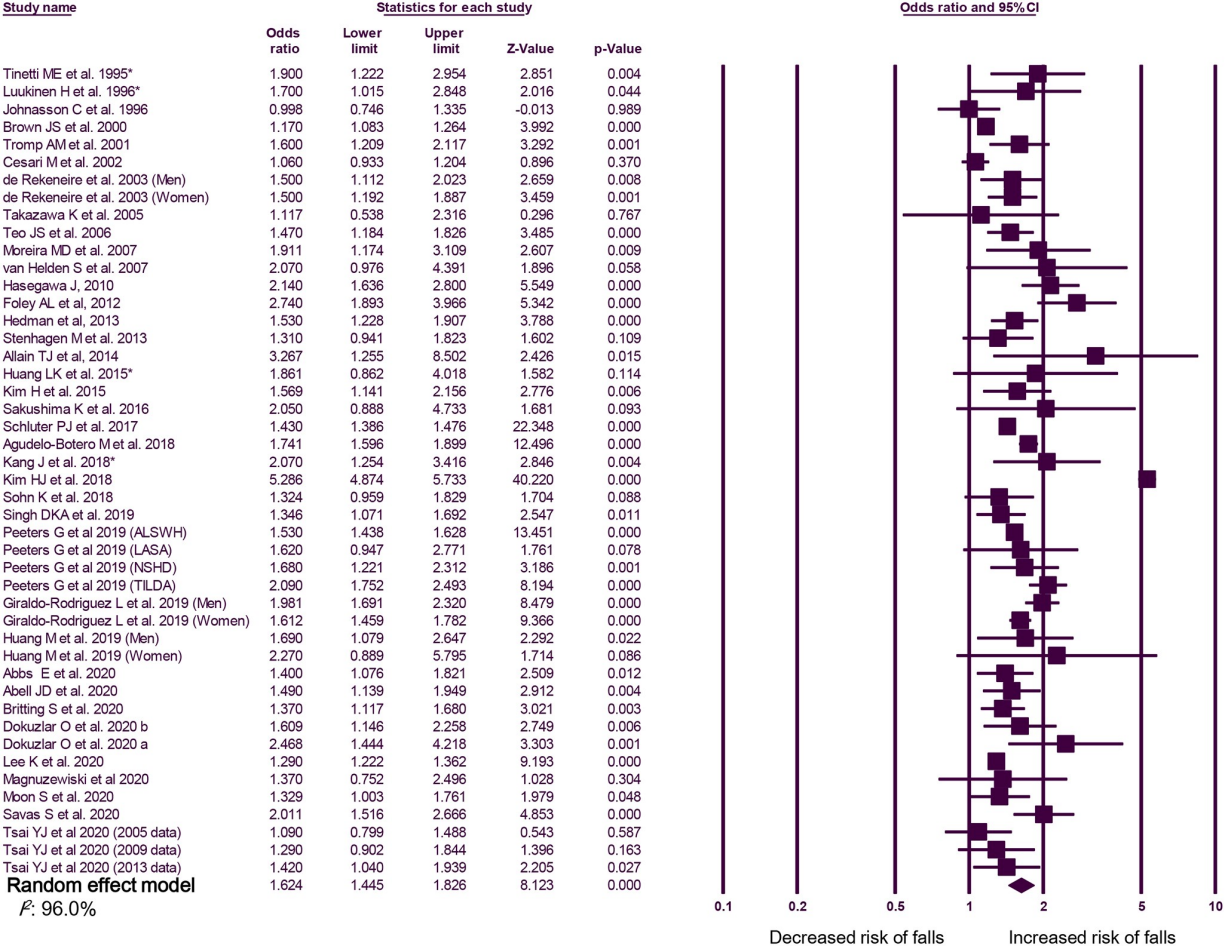
Forest plots of the risk ratio of the association between urinary incontinence and falls. OR, odds ratio; CI, confidence interval. *Study that defined falls as at least two falls within 1 year.

**Fig 4:**
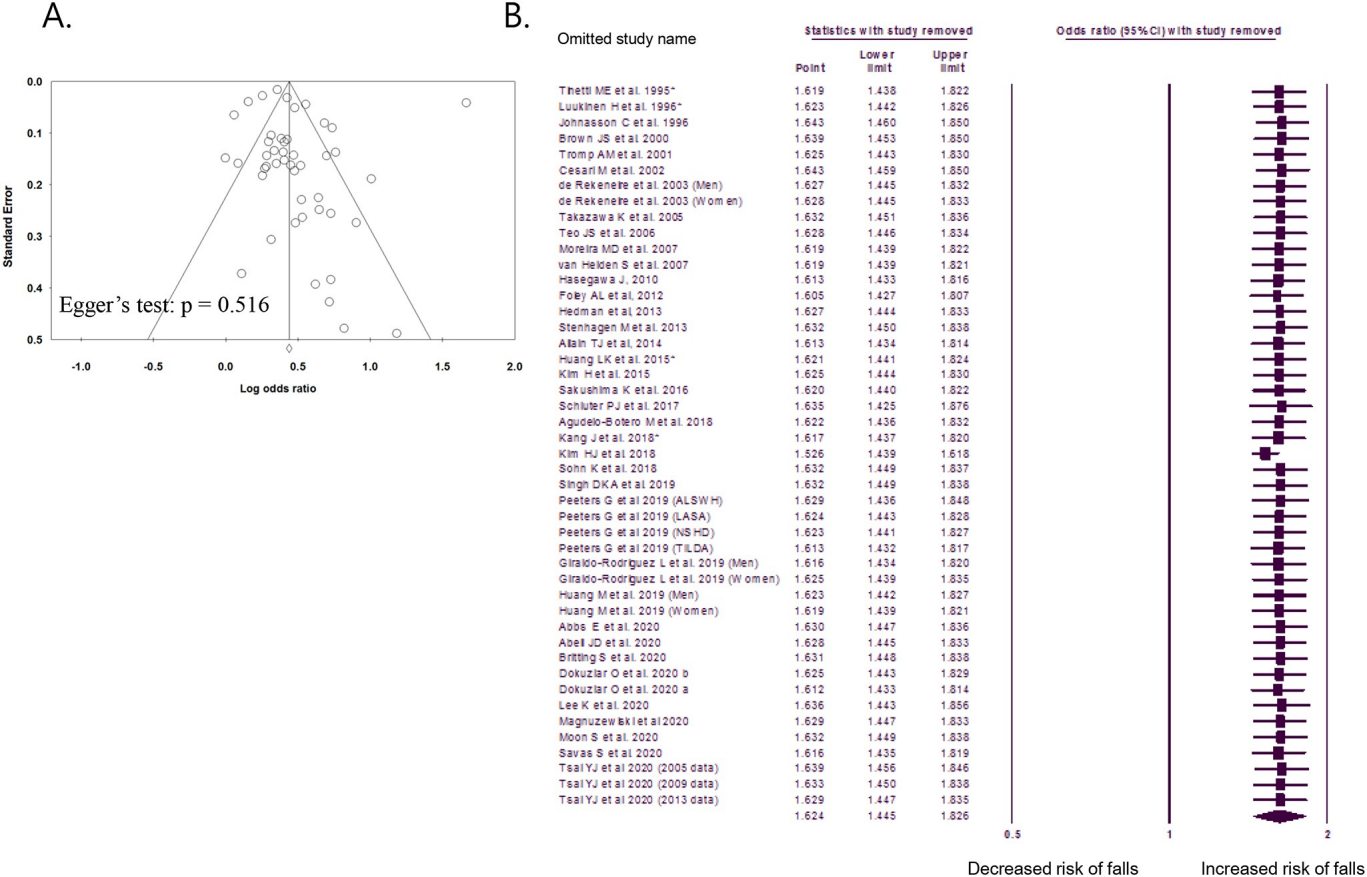
Funnel plot and sensitivity analysis. A. Funnel plot of publication bias in studies comparing the odds ratios of urinary incontinence for falls. B. Sensitivity analysis of the meta-analysis of studies comparing the odds ratios of urinary incontinence for falls. *Study that defined falls as at least two falls within 1 year.

According to the random-effects model, the overall OR for falls was 1.62 (95% CI, 1.45–1.83). An overall I^2^ of 96.0% indicated heterogeneity among the studies (Fig 3). The funnel plot and Egger’s test did not reveal any publication bias (p = 0.477, Fig 4A). The sensitivity analysis revealed consistently significant ORs between 1.55 and 1.67, even after excluding the results of each included study (Fig 4B). After excluding 14 studies with a critical risk of bias, the OR was 1.46 (95% CI, 1.38–1.56; I^2^, 76.5%).

### Analyses of subgroups stratified by age, sex, the definition of falls, and type of UI

Subgroup analyses were performed according to the age and sex of the participants (Table 2). A significant association between UI and falls was observed in older adults (≥65 years; OR, 1.59; 95% CI, 1.31–1.93) [2, 7–10, 14–17, 21–23, 25–27, 31, 34–37, 40, 43], and in both men (OR, 1.88; 95% CI, 1.57–2.25) [17, 23, 29–31, 36] and women (OR, 1.41; 95% CI, 1.29–1.54) [2, 7, 9, 10, 17, 18, 22, 23, 27, 29–31, 35, 45]. In a subgroup analysis of 34 studies that defined falls as ≥1 fall event, the OR for the association between UI and falls was 1.61 (95% CI, 1.42–1.82; I^2^, 96.3%; Table 2) [2, 7–11, 16–20, 22–24, 26–40, 42–46]. In a subgroup analysis of nine studies that defined recurrent falls as ≥2 fall events, the OR for the association between UI and falls was 1.63 (95% CI, 1.49–1.78; I^2^, 40.6%; Table 2) [2, 10, 14–16, 19, 21, 24, 25]. In a subgroup analysis according to the type of UI, a significant association between UI and falls was observed in patients with urgency UI (OR, 1.76; 95% CI, 1.15–1.70) [7, 8, 10, 18, 30] and in those with stress UI (OR, 1.73; 95% CI, 1.39–2.15) [7, 8, 10, 18, 30].

**Table 2:** Subgroup analysis of the association between urinary incontinence and falls.

Subgroup	No. of studies [Reference]	OR (95% CI)	Heterogeneity (I^2^), %
Age, ≥ 65 years	22 [2, 7–10, 14–17, 21–23, 25–27, 31, 34–37, 40, 43]	1.59 (1.31–1.93)	97.6%
Sex			
Men	6 [17, 23, 29–31, 36]	1.88 (1.57–2.25)	75.2%
Women	14 [2, 7, 9, 10, 17, 18, 22, 23, 27, 29–31, 35, 45]	1.41 (1.29–1.54)	79.5%
Definition of falls			
Falls ≥ 1	34 [2, 7–11, 16–20, 22–24, 26–40, 42–46]	1.61 (1.42–1.82)	96.3%
Falls ≥ 2	9 [2, 10, 14–16, 19, 21, 24, 25]	1.63 (1.49–1.78)	40.6%
Type of urinary incontinence			
Urgency incontinence	5 [7, 8, 10, 18, 30]	1.76 (1.15–1.70)	97.1%
Stress incontinence	5 [7, 8, 10, 18, 30]	1.73 (1.39–2.15)	90.2%

## Discussion

Although UI is a known risk factor for falls, the strength of the association between these conditions remains unclear because of variability in the study designs and populations used in previous risk estimations. This systematic review and meta-analysis conducted to evaluate the association between falls and UI revealed that UI was associated with overall falls. Our analysis identified a probable excess OR of 65% for at least one fall among people with UI relative to those without UI. An analysis of participants with recurrent falls yielded a similar trend and a higher risk magnitude. The overall OR for recurrent falls was 63% among people with UI relative to those without UI.

In a subgroup analysis, we determined that the OR for falls increased by 59% in older adults (≥65 years) with UI relative to those without UI. These findings exceed those of older systematic reviews that considered a more limited range of fall-related outcomes and consistently reported an increased risk of falls and fractures among participants with UI [47]. UI is of significant concern to older adults and can lead to isolation and reduced self-worth. Previous studies have identified various risk factors for falls, such as old age, female sex, visual disturbances, cognitive disorders, low body mass index, and UI.

We conducted another subgroup analysis according to the type of UI. A previous review highlighted a predominant association of falls with urgency UI, rather than with other types of UI [47]. This association is attributed to the urgent need to use the toilet and the anxiety associated with a failure to reach the toilet. Several studies have shown that behavioral changes induced by UI can affect the likelihood of falls [48, 49]. Our analysis also showed a higher risk of falls in patients with urgency UI than in those with stress UI. Falls related to this condition have been generally reported to occur in the toilet [7, 47]. Despite this relationship, however, the commonly held assumption that urgency leads to falls while rushing to the toilet has not been confirmed yet [6].

Few studies have investigated the relationship between UI and falls [47], and the causality between UI and falls remains unexplained [6]. However, one hypothesis is that a strong desire to void could change gait parameters and thus, increase the risk of falls [50]. The reduced velocity and stride width during strong desire to void conditions (i.e., urgency) in the UI group could explain their high fall rate [50]. The other hypothesis is that women with impaired mobility probably take a longer time to reach the toilet; hence, if there is a high degree of urgency, then impaired mobility can increase the risk of UI [51]. Therefore, the causality between UI and falls could probably be explained by a strong desire to void and physical impairments in mobility and balance [50, 51]. However, although these hypotheses could explain the relationship between the urgency-type UI and falls, they are rather insufficient to explain the association between stress-type UI and falls. Since the symptoms of urgency UI and stress UI are clinically different, the association between stress UI and falls may indicate a general alteration in the striated muscle physiology in the aging population [8]. In addition, restricted mobility in older women may limit their ability to change positions to prevent stress UI [22].

There is a well-recognized association between falls and lower urinary tract symptoms (LUTS) in older adults [7, 8, 47, 52, 53]. Older people with urgency or urgency UI are significantly more likely to fall than age-matched controls, with ORs for falls ranging between 1.5 and 2.3 [6, 47, 54, 55]. However, the reason for this association is not understood and has not been thoroughly studied [6].

In a recent systemic review on the association between falls and LUTS conducted by Noguchi et al., none of the identified studies had investigated the potential causes of these associations. In addition, the categorization of UI and degree of accounting for confounding variables were inconsistent across the studies [56]. Although the data identified were suitable only for qualitative synthesis, UI and storage symptoms among LUTS have been consistently reported to have a weak to moderate association with falls [6, 56].

As our findings suggest that this association is significant, the identification and treatment of UI may be an effective intervention for reducing the risk of falls, especially in older adults. Bladder training, timed or prompt voiding, and environmental modifications (e.g., a bedside commode) may decrease the incidence of falls [7].

Concerning the impact of UI on the risk of falling, many falls are related to a person’s physical condition or medical problems, such as multimorbidities, polypharmacy, neurological diseases, and sarcopenia, as well as urological comorbidities [57]. Especially, multiple medications, such as blood pressure-lowering drugs causing orthostatic hypotension, psychotropics, anticonvulsants, and sedatives, can contribute to falls [57]. In addition, the geriatric syndrome has a multifactorial etiology, with the factors being closely related to each other [1]. Among them, UI and falls are very important for the older population, and both are associated with sarcopenia [8, 58, 59]. Therefore, an appropriate statistical approach to decrease the impact of such confounding variables is necessary for correct analysis of the association between UI and falls.

The strengths of this study include the collection of evidence through a rigorous systematic review and meta-analysis. This study also included a comprehensive search of both published and unpublished studies. Multiple measurements of falls were considered, consistent with multiple types of risk estimates. Although many studies have included UI as a risk factor for falls, only a few studies have identified UI as an individual risk factor [47]. Therefore, this is the first systematic review and meta-analysis to evaluate UI as an individual risk factor for falls.

Despite these strengths, our study was limited largely by the included studies, particularly the significant heterogeneity, quality of the study designs, and reporting scope of the original articles. However, when studies with a critical risk of bias were excluded, significant results were observed. In addition, no publication bias was observed, and the results were not changed by specific studies in the sensitivity analysis. Furthermore, although we conducted subgroup analyses based on age, sex, and type of UI, we did not perform analyses according to the severity of UI. Finally, the paucity of evidence regarding the severity of UI limits the applicability of our current findings with regard to an accurate correlation between UI and falls.

In conclusion, the continued increase in the proportion of older adults globally will lead to continued increases in the clinical and economic impacts of serious falls. Based on evidence from the published literature and a meta-analysis, we demonstrate here that UI is a predictor of more frequent falls in both general and older adults. Clinicians should, therefore, be aware that UI predicts an increased risk of falls that could lead to fractures and should, therefore, provide appropriate precautions and care. Future studies are needed to address the impact of UI treatment on the incidence of falls.

## Supporting information

S1 Table PRISMA checklist. (DOCX)

S2 Table Electronic search strategy. (DOCX)

S1 Data PubMed: 286 studies. (DOCX)

## Abbreviations

CI: confidence interval
LUTS: lower urinary tract symptoms
OR: odds ratio
UI: urinary incontinence
WHO: World Health Organization
