# Supplementary material for: The impact of urinary incontinence on falls: A systematic review and meta-analysis
Source: PLoS One. 2021 May 19;16(5):e0251711. doi: 10.1371/journal.pone.0251711 (PMC8133449; doi:10.1371/journal.pone.0251711)
Supplement: S2 Table — (DOCX) [file pone.0251711.s002.docx]

**S2 Table.** Electronic search strategy

| **Database** | **Search term (inception to December 13, 2020)** | **Number of studies** |
| --- | --- | --- |
| **PubMed**  **No filters activated** | **(Urinary incontinence) AND (fall)** | **286** |
| **EMBASE**  **No filters activated** | **‘Urinary incontinence’ AND ‘fall’** | **439** |
| **Web of Science**  **Core Collection** | **(Urinary incontinence) AND (fall)** | **702** |
