# Supplementary material for: The impact of urinary incontinence on falls: A systematic review and meta-analysis
Source: PLoS One. 2021 May 19;16(5):e0251711. doi: 10.1371/journal.pone.0251711 (PMC8133449; doi:10.1371/journal.pone.0251711)
Supplement: S1 Data — (DOCX) [file pone.0251711.s003.docx]

**PubMed: 286 studies**

1. Omae K, Kurita N, Takeshima T, Naganuma T, Takahashi S, Yoshioka T, et al. Significance of Overactive Bladder as a Predictor of Falls in Community Dwelling Older Adults: 1-Year Followup of the Sukagawa Study. The Journal of urology. 2021;205(1):219-25.

2. Zullo AR, Sorial MN, Lee Y, Lary CW, Kiel DP, Berry SD. Predictors of Hip Fracture Despite Treatment with Bisphosphonates among Frail Older Adults. Journal of the American Geriatrics Society. 2020;68(2):256-60.

3. Yalcintas E, Demirci H, Aykurt Karlibel I, Turkoglu AR, Kasapoglu Aksoy M, Coban S. Geriatric giants in women over 65 years living in a rural area in Turkey. Journal of women & aging. 2020:1-7.

4. Tsai YJ, Yang PY, Yang YC, Lin MR, Wang YW. Prevalence and risk factors of falls among community-dwelling older people: results from three consecutive waves of the national health interview survey in Taiwan. BMC geriatrics. 2020;20(1):529.

5. Siegal AR, Huang Z, Gross MD, Mehraban-Far S, Weissbart SJ, Kim JM. Trends of Mesh Utilization for Stress Urinary Incontinence Before and After the 2011 Food and Drug Administration Notification Between FPMRS-Certified and Non-FPMRS-Certified Physicians: A Statewide All-Payer Database Analysis. Urology. 2020.

6. Shin J, Han SH, Choi J, Kim YS, Lee J. Importance of Geriatric Syndrome Screening within 48 Hours of Hospitalization for Identifying Readmission Risk: A Retrospective Study in an Acute-Care Hospital. Annals of geriatric medicine and research. 2020;24(2):83-90.

7. Roggeman S, Weiss JP, Van Laecke E, Vande Walle J, Everaert K, Bower WF. The role of lower urinary tract symptoms in fall risk assessment tools in hospitals: a review. F1000Research. 2020;9.

8. Paquin MH, Duclos C, Lapierre N, Dubreucq L, Morin M, Meunier J, et al. The effects of a strong desire to void on gait for incontinent and continent older community-dwelling women at risk of falls. Neurourology and urodynamics. 2020;39(2):642-9.

9. Moon S, Chung HS, Yu JM, Na HR, Kim SJ, Ko KJ, et al. Impact of urinary incontinence on falls in the older population: 2017 national survey of older Koreans. Archives of gerontology and geriatrics. 2020;90:104158.

10. Magnuszewski L, Swietek M, Kasiukiewicz A, Kuprjanowicz B, Baczek J, Beata Wojszel Z. Health, Functional and Nutritional Determinants of Falls Experienced in the Previous Year-A Cross-Sectional Study in a Geriatric Ward. International journal of environmental research and public health. 2020;17(13).

11. Lee K, Davis MA, Marcotte JE, Pressler SJ, Liang J, Gallagher NA, et al. Falls in community-dwelling older adults with heart failure: A retrospective cohort study. Heart & lung : the journal of critical care. 2020;49(3):238-50.

12. Konishi S, Hatakeyama S, Imai A, Kumagai M, Okita K, Togashi K, et al. Overactive bladder and sleep disturbance have a significant effect on indoor falls: Results from the community health survey in Japan. Lower urinary tract symptoms. 2020.

13. Hentzen C, Villaumé A, Turmel N, Chesnel C, Le Breton F, Haddad R, et al. Are falls in people with multiple sclerosis related to the severity of urinary disorders? Annals of physical and rehabilitation medicine. 2020:101452.

14. Dokuzlar O, Koc Okudur S, Soysal P, Kocyigit SE, Yavuz I, Smith L, et al. Factors that Increase Risk of Falling in Older Men according to Four Different Clinical Methods. Experimental aging research. 2020;46(1):83-92.

15. Dokuzlar O, Koc Okudur S, Smith L, Soysal P, Yavuz I, Aydin AE, et al. Assessment of factors that increase risk of falling in older women by four different clinical methods. Aging clinical and experimental research. 2020;32(3):483-90.

16. Córcoles-Jiménez MP, Candel-Parra E, Del Egido-Fernández M, Villada-Munera A, Moreno-Moreno M, Piña-Martínez AJ, et al. Preventing Functional Urinary Incontinence in Hip-Fractured Older Adults Through Patient Education: A Randomized Controlled Trial. Journal of applied gerontology : the official journal of the Southern Gerontological Society. 2020:733464820952608.

17. Chen PL, Lin HY, Ong JR, Ma HP. Development of a fall-risk assessment profile for community-dwelling older adults by using the National Health Interview Survey in Taiwan. BMC public health. 2020;20(1):234.

18. Britting S, Artzi-Medvedik R, Fabbietti P, Tap L, Mattace-Raso F, Corsonello A, et al. Kidney function and other factors and their association with falls : The screening for CKD among older people across Europe (SCOPE) study. BMC geriatrics. 2020;20(Suppl 1):320.

19. Barr PJ, Berry SA, Gozansky WS, McQuillan DB, Ross C, Carmichael D, et al. No date for the PROM: the association between patient-reported health events and clinical coding in primary care. Journal of patient-reported outcomes. 2020;4(1):17.

20. Aranyavalai T, Jalayondeja C, Jalayondeja W, Pichaiyongwongdee S, Kaewkungwal J, Laskin JJ. Association between walking 5000 step/day and fall incidence over six months in urban community-dwelling older people. BMC geriatrics. 2020;20(1):194.

21. Alex D, Khor HM, Chin AV, Hairi NN, Cumming RG, Othman S, et al. Factors Associated With Falls Among Urban-Dwellers Aged 55 Years and Over in the Malaysian Elders Longitudinal Research (MELoR) Study. Frontiers in public health. 2020;8:506238.

22. Abell JG, Lassale C, Batty GD, Zaninotto P. Risk factors for hospital admission after a fall: a prospective cohort study of community-dwelling older people. The journals of gerontology Series A, Biological sciences and medical sciences. 2020.

23. Abbs E, Brown R, Guzman D, Kaplan L, Kushel M. Risk Factors for Falls in Older Adults Experiencing Homelessness: Results from the HOPE HOME Cohort Study. Journal of general internal medicine. 2020;35(6):1813-20.

24. Winoker JS, Say RK, Mehrazin R, Stock RG, Stone NN. Permanent prostate brachytherapy is safe in men with severe baseline lower urinary tract symptoms. Brachytherapy. 2019;18(3):332-7.

25. Thapa S, Shmerling RH, Bean JF, Cai Y, Leveille SG. Chronic multisite pain: evaluation of a new geriatric syndrome. Aging clinical and experimental research. 2019;31(8):1129-37.

26. Takeuchi T, Yajima K. Long-term 4 Years Follow-up Study of 482 Patients Who Underwent Shunting for Idiopathic Normal Pressure Hydrocephalus -Course of Symptoms and Shunt Efficacy Rates Compared by Age Group. Neurologia medico-chirurgica. 2019;59(7):281-6.

27. Shin JH. Nursing Staff Characteristics on Resident Outcomes in Nursing Homes. The journal of nursing research : JNR. 2019;27(1):1-9.

28. Peeters G, Cooper R, Tooth L, van Schoor NM, Kenny RA. A comprehensive assessment of risk factors for falls in middle-aged adults: co-ordinated analyses of cohort studies in four countries. Osteoporosis international : a journal established as result of cooperation between the European Foundation for Osteoporosis and the National Osteoporosis Foundation of the USA. 2019;30(10):2099-117.

29. Oshiro CES, Frankland TB, Rosales AG, Perrin NA, Bell CL, Lo SHY, et al. Fall Ascertainment and Development of a Risk Prediction Model Using Electronic Medical Records. Journal of the American Geriatrics Society. 2019;67(7):1417-22.

30. Najafpour Z, Godarzi Z, Arab M, Yaseri M. Risk Factors for Falls in Hospital In-Patients: A Prospective Nested Case Control Study. International journal of health policy and management. 2019;8(5):300-6.

31. Mallol D, Taveras R, Hartman J, Granville M, Jacobson RE. Cauda Equina Syndrome in a Patient with Intradural Schwannoma at the Same Level as an Acute L2 Compression Fracture. Cureus. 2019;11(8):e5492.

32. Korall AMB, Feldman F, Yang Y, Cameron ID, Leung PM, Sims-Gould J, et al. Effectiveness of Hip Protectors to Reduce Risk for Hip Fracture from Falls in Long-Term Care. Journal of the American Medical Directors Association. 2019;20(11):1397-403.e1.

33. Juliato CRT, Santos-Junior LC, de Castro EB, Dertkigil SS, Brito LGO. Vaginal axis after abdominal sacrocolpopexy versus vaginal sacrospinous fixation-a randomized trial. Neurourology and urodynamics. 2019;38(4):1142-51.

34. Huang MH, Blackwood J, Godoshian M, Pfalzer L. Predictors of falls in older survivors of breast and prostate cancer: A retrospective cohort study of surveillance, epidemiology and end results-Medicare health outcomes survey linkage. Journal of geriatric oncology. 2019;10(1):89-97.

35. Falcão RMM, Costa K, Fernandes M, Pontes MLF, Vasconcelos JMB, Oliveira JDS. Risk of falls in hospitalized elderly people. Revista gaucha de enfermagem. 2019;40(spe):e20180266.

36. Escobar-Aguilar G, Moreno-Casbas MT, González-María E, Martínez-Gimeno ML, Sánchez-Pablo C, Orts-Cortés I. The SUMAMOS EXCELENCIA Project. Journal of advanced nursing. 2019;75(7):1575-84.

37. Eglseer D, Hödl M, Lohrmann C. Six Nursing Care Problems in Hospitals: A Cross-Sectional Study of Quality of Care. Journal of nursing care quality. 2019;34(1):E8-e14.

38. Chu CM, Schmitz KH, Khanijow K, Stambakio H, Newman DK, Arya LA, et al. Feasibility and outcomes: Pilot Randomized Controlled Trial of a home-based integrated physical exercise and bladder-training program vs usual care for community-dwelling older women with urinary incontinence. Neurourology and urodynamics. 2019;38(5):1399-408.

39. Trabuco EC, Montori VM. The Rise and Fall of Mesh in Pelvic Surgery and the Shortcomings of Medical Device Regulation. Obstetrics and gynecology. 2018;132(3):736-40.

40. Tatum Iii PE, Talebreza S, Ross JS. Geriatric Assessment: An Office-Based Approach. American family physician. 2018;97(12):776-84.

41. Sohn K, Lee CK, Shin J, Lee J. Association between Female Urinary Incontinence and Geriatric Health Problems: Results from Korean Longitudinal Study of Ageing (2006). Korean journal of family medicine. 2018;39(1):10-4.

42. Smith EM, Shah AA. Screening for Geriatric Syndromes: Falls, Urinary/Fecal Incontinence, and Osteoporosis. Clinics in geriatric medicine. 2018;34(1):55-67.

43. Onder G, Giovannini S, Sganga F, Manes-Gravina E, Topinkova E, Finne-Soveri H, et al. Interactions between drugs and geriatric syndromes in nursing home and home care: results from Shelter and IBenC projects. Aging clinical and experimental research. 2018;30(9):1015-21.

44. Liang Y, Rausch C, Laflamme L, Möller J. Prevalence, trend and contributing factors of geriatric syndromes among older Swedes: results from the Stockholm County Council Public Health Surveys. BMC geriatrics. 2018;18(1):322.

45. Kim HJ, Kim JW, Jang SN, Kim KD, Yoo JI, Ha YC. Urinary Incontinences Are Related with Fall and Fragility Fractures in Elderly Population: Nationwide Cohort Study. Journal of bone metabolism. 2018;25(4):267-74.

46. Elghblawi E. Platelet-rich plasma, the ultimate secret for youthful skin elixir and hair growth triggering. Journal of cosmetic dermatology. 2018;17(3):423-30.

47. Brittain C, Ambegaonkar G. Post-traumatic seizure with an unexpected finding. Archives of disease in childhood Education and practice edition. 2018;103(6):302-3.

48. Agudelo-Botero M, Giraldo-Rodríguez L, Murillo-González JC, Mino-León D, Cruz-Arenas E. Factors associated with occasional and recurrent falls in Mexican community-dwelling older people. PloS one. 2018;13(2):e0192926.

49. Zelaya JE, Murchison C, Cameron M. Associations Between Bladder Dysfunction and Falls in People with Relapsing-Remitting Multiple Sclerosis. International journal of MS care. 2017;19(4):184-90.

50. Yu WC, Chou MY, Peng LN, Lin YT, Liang CK, Chen LK. Synergistic effects of cognitive impairment on physical disability in all-cause mortality among men aged 80 years and over: Results from longitudinal older veterans study. PloS one. 2017;12(7):e0181741.

51. Talarska D, Strugała M, Szewczyczak M, Tobis S, Michalak M, Wróblewska I, et al. Is independence of older adults safe considering the risk of falls? BMC geriatrics. 2017;17(1):66.

52. Solis GR, Champion JD. Examining Fall Recurrence Risk of Homebound Hispanic Older Adults Receiving Home Care Services. Hispanic health care international : the official journal of the National Association of Hispanic Nurses. 2017;15(1):20-6.

53. Mooventhan A, Nivethitha L. Evidence based effects of yoga practice on various health related problems of elderly people: A review. Journal of bodywork and movement therapies. 2017;21(4):1028-32.

54. Moga DC, Wu Q, Doshi P, Goodin AJ. An investigation of factors predicting the type of bladder antimuscarinics initiated in Medicare nursing homes residents. BMC geriatrics. 2017;17(1):295.

55. Locatelli I, Monod S, Cornuz J, Büla CJ, Senn N. A prospective study assessing agreement and reliability of a geriatric evaluation. BMC geriatrics. 2017;17(1):153.

56. Liang CK, Chou MY, Chen LY, Wang KY, Lin SY, Chen LK, et al. Delaying cognitive and physical decline through multidomain interventions for residents with mild-to-moderate dementia in dementia care units in Taiwan: A prospective cohort study. Geriatrics & gerontology international. 2017;17 Suppl 1:36-43.

57. Kaur H, Bala R, Nagpal M. Role of Vitamin D in urogenital health of geriatric participants. Journal of mid-life health. 2017;8(1):28-35.

58. Hung CH, Wang CJ, Tang TC, Chen LY, Peng LN, Hsiao FY, et al. Recurrent falls and its risk factors among older men living in the veterans retirement communities: A cross-sectional study. Archives of gerontology and geriatrics. 2017;70:214-8.

59. Dahodwala N, Nwadiogbu C, Fitts W, Partridge H, Karlawish J. Parkinsonian signs are a risk factor for falls. Gait & posture. 2017;55:1-5.

60. Balachandran A, Duckett J. Cystodistension: Is there evidence to support its use in current practice for patients with overactive bladder? Journal of obstetrics and gynaecology : the journal of the Institute of Obstetrics and Gynaecology. 2017;37(6):700-3.

61. Allen C, Zarowitz BJ, O'Shea T, Datto C, Olufade T. Clinical and Functional Characteristics of Nursing Facility Residents with Opioid-Induced Constipation. The Consultant pharmacist : the journal of the American Society of Consultant Pharmacists. 2017;32(5):285-98.

62. Zhang D, He Y, Liu M, Yang HB, Wu L, Wang JH, et al. [Study on incidence and risk factors of fall in the elderly in a rural community in Beijing]. Zhonghua liu xing bing xue za zhi = Zhonghua liuxingbingxue zazhi. 2016;37(5):624-8.

63. Sakushima K, Yamazaki S, Fukuma S, Hayashino Y, Yabe I, Fukuhara S, et al. Influence of urinary urgency and other urinary disturbances on falls in Parkinson's disease. Journal of the neurological sciences. 2016;360:153-7.

64. Pinkas J, Gujski M, Humeniuk E, Raczkiewicz D, Bejga P, Owoc A, et al. State of Health and Quality of Life of Women at Advanced Age. Medical science monitor : international medical journal of experimental and clinical research. 2016;22:3095-105.

65. Pahwa AK, Andy UU, Newman DK, Stambakio H, Schmitz KH, Arya LA. Noctural Enuresis as a Risk Factor for Falls in Older Community Dwelling Women with Urinary Incontinence. The Journal of urology. 2016;195(5):1512-6.

66. Noguchi N, Chan L, Cumming RG, Blyth FM, Handelsman DJ, Seibel MJ, et al. Lower Urinary Tract Symptoms and Incident Falls in Community Dwelling Older Men: The Concord Health and Ageing in Men Project. The Journal of urology. 2016;196(6):1694-9.

67. Minasian T, Claus C, Hariri OR, Piao Z, Quadri SA, Yuhan R, et al. Chondromyxoid fibroma of the sacrum: A case report and literature review. Surgical neurology international. 2016;7(Suppl 13):S370-4.

68. Lukaszyk C, Harvey L, Sherrington C, Keay L, Tiedemann A, Coombes J, et al. Risk factors, incidence, consequences and prevention strategies for falls and fall-injury within older indigenous populations: a systematic review. Australian and New Zealand journal of public health. 2016;40(6):564-8.

69. Gibson W, Wagg A. Are older women more likely to receive surgical treatment for stress urinary incontinence since the introduction of the mid-urethral sling? An examination of Hospital Episode Statistics data. BJOG : an international journal of obstetrics and gynaecology. 2016;123(8):1386-92.

70. Freemantle N, Khalaf K, Loveman C, Stanisic S, Gultyaev D, Lister J, et al. OnabotulinumtoxinA in the treatment of overactive bladder: a cost-effectiveness analysis versus best supportive care in England and Wales. The European journal of health economics : HEPAC : health economics in prevention and care. 2016;17(7):911-21.

71. Danzer E, Thomas NH, Thomas A, Friedman KB, Gerdes M, Koh J, et al. Long-term neurofunctional outcome, executive functioning, and behavioral adaptive skills following fetal myelomeningocele surgery. American journal of obstetrics and gynecology. 2016;214(2):269.e1-.e8.

72. Brown J, King J. Age-stratified trends in 20 years of stress incontinence surgery in Australia. The Australian & New Zealand journal of obstetrics & gynaecology. 2016;56(2):192-8.

73. Bradley D, Hsueh W. Type 2 Diabetes in the Elderly: Challenges in a Unique Patient Population. Journal of geriatric medicine and gerontology. 2016;2(2).

74. Boronat-Garrido X, Kottner J, Schmitz G, Lahmann N. Incontinence-Associated Dermatitis in Nursing Homes: Prevalence, Severity, and Risk Factors in Residents With Urinary and/or Fecal Incontinence. Journal of wound, ostomy, and continence nursing : official publication of The Wound, Ostomy and Continence Nurses Society. 2016;43(6):630-5.

75. van Houten P. [Urinary incontinence in the elderly can be treated]. Nederlands tijdschrift voor geneeskunde. 2015;159:A9692.

76. Tannenbaum C, van den Heuvel E, Fritel X, Southall K, Jutai J, Rajabali S, et al. Continence Across Continents To Upend Stigma and Dependency (CACTUS-D): study protocol for a cluster randomized controlled trial. Trials. 2015;16:565.

77. Shin JH, Hyun TK. Nurse Staffing and Quality of Care of Nursing Home Residents in Korea. Journal of nursing scholarship : an official publication of Sigma Theta Tau International Honor Society of Nursing. 2015;47(6):555-64.

78. Lima CA, Soares WJ, Bilton TL, Dias RC, Ferrioll E, Perracini MR. Correlates of excessive daytime sleepiness in community-dwelling older adults: an exploratory study. Revista brasileira de epidemiologia = Brazilian journal of epidemiology. 2015;18(3):607-17.

79. Edwards R, Hunter K, Wagg A. Lower urinary tract symptoms and falls in older women: a case control study. Maturitas. 2015;80(3):308-11.

80. Beegan L, Messinger-Rapport BJ. Stand by me! Reducing the risk of injurious falls in older adults. Cleveland Clinic journal of medicine. 2015;82(5):301-7.

81. Ajmera M, Raval A, Zhou S, Wei W, Bhattacharya R, Pan C, et al. A Real-World Observational Study of Time to Treatment Intensification Among Elderly Patients with Inadequately Controlled Type 2 Diabetes Mellitus. Journal of managed care & specialty pharmacy. 2015;21(12):1184-93.

82. Abreu HC, Reiners AA, Azevedo RC, Silva AM, Abreu D, Oliveira A. Incidence and predicting factors of falls of older inpatients. Revista de saude publica. 2015;49:37.

83. Sammels M, Vandesande J, Vlaeyen E, Peerlinck K, Milisen K. Falling and fall risk factors in adults with haemophilia: an exploratory study. Haemophilia : the official journal of the World Federation of Hemophilia. 2014;20(6):836-45.

84. Rafiq M, McGovern A, Jones S, Harris K, Tomson C, Gallagher H, et al. Falls in the elderly were predicted opportunistically using a decision tree and systematically using a database-driven screening tool. Journal of clinical epidemiology. 2014;67(8):877-86.

85. Orces CH. Prevalence and Determinants of Fall-Related Injuries among Older Adults in Ecuador. Current gerontology and geriatrics research. 2014;2014:863473.

86. Lino VT, Portela MC, Camacho LA, Rodrigues NC. Reliability of screening tests for health-related problems among low-income elderly. Cadernos de saude publica. 2014;30(12):2691-6.

87. Lim SC, Mamun K, Lim JK. Comparison between elderly inpatient fallers with and without dementia. Singapore medical journal. 2014;55(2):67-71.

88. Lahmann NA, Heinze C, Rommel A. [Falls in German hospitals and nursing homes 2006-2013. Frequencies, injuries, risk assessment, and preventive measures]. Bundesgesundheitsblatt, Gesundheitsforschung, Gesundheitsschutz. 2014;57(6):650-9.

89. Jha S. The rise and fall of the vaginal mesh. BJOG : an international journal of obstetrics and gynaecology. 2014;121(11):1438.

90. Hofmann H, Hahn S. Characteristics of nursing home residents and physical restraint: a systematic literature review. Journal of clinical nursing. 2014;23(21-22):3012-24.

91. Fraser SA, Elliott V, de Bruin ED, Bherer L, Dumoulin C. The Effects of Combining Videogame Dancing and Pelvic Floor Training to Improve Dual-Task Gait and Cognition in Women with Mixed-Urinary Incontinence. Games for health journal. 2014;3(3):172-8.

92. Dellaroza MS, Pimenta CA, Lebrão ML, Duarte YA, Braga PE. [Association between chronic pain and self-reported falls in the SABE study population]. Cadernos de saude publica. 2014;30(3):522-32.

93. Bresee C, Dubina ED, Khan AA, Sevilla C, Grant D, Eilber KS, et al. Prevalence and correlates of urinary incontinence among older community-dwelling women. Female pelvic medicine & reconstructive surgery. 2014;20(6):328-33.

94. Andreasson A, Fall M, Persson E, Stranne J, Peeker R. High revision rate following artificial urethral sphincter implantation. Scandinavian journal of urology. 2014;48(6):544-8.

95. Abreu HC, Reiners AA, Azevedo RC, Silva AM, Abreu DR. [Urinary incontinence in the prediction of falls in hospitalized elderly]. Revista da Escola de Enfermagem da U S P. 2014;48(5):851-6.

96. Pierorazio PM, Mullins JK, Ross AE, Hyams ES, Partin AW, Han M, et al. Trends in immediate perioperative morbidity and delay in discharge after open and minimally invasive radical prostatectomy (RP): a 20-year institutional experience. BJU international. 2013;112(1):45-53.

97. Orces CH. Prevalence and Determinants of Falls among Older Adults in Ecuador: An Analysis of the SABE I Survey. Current gerontology and geriatrics research. 2013;2013:495468.

98. Kim H, Suzuki T, Yoshida H, Shimada H, Yamashiro Y, Sudo M, et al. [Are gait parameters related to knee pain, urinary incontinence and a history of falls in community-dwelling elderly women?]. Nihon Ronen Igakkai zasshi Japanese journal of geriatrics. 2013;50(4):528-35.

99. Jojima T, Aso Y. [Attention to the use of oral anti-diabetic medication in older adults with type 2 diabetes]. Nihon rinsho Japanese journal of clinical medicine. 2013;71(11):1987-92.

100. Hussain A, Pansota MS, Rasool M, Tabassum SA, Ahmad I, Saleem MS. Outcome of end-to-end urethroplasty in post-traumatic stricture of posterior urethra. Journal of the College of Physicians and Surgeons--Pakistan : JCPSP. 2013;23(4):272-5.

101. Hersh L, Salzman B. Clinical management of urinary incontinence in women. American family physician. 2013;87(9):634-40.

102. Damián J, Pastor-Barriuso R, Valderrama-Gama E, de Pedro-Cuesta J. Factors associated with falls among older adults living in institutions. BMC geriatrics. 2013;13:6.

103. Batchelor FA, Dow B, Low MA. Do continence management strategies reduce falls? a systematic review. Australasian journal on ageing. 2013;32(4):211-6.

104. Arnold PM, Teuber J. Marfan syndrome and symptomatic sacral cyst: report of two cases. The journal of spinal cord medicine. 2013;36(5):499-503.

105. Toba K. [Locomotive syndrome and frailty. Frail elderly]. Clinical calcium. 2012;22(4):13-9.

106. Miller KL, Baraldi CA. Geriatric gynecology: promoting health and avoiding harm. American journal of obstetrics and gynecology. 2012;207(5):355-67.

107. Lamartina C. Expert's comment concerning Grand Rounds case entitled "Limited access surgery for 360 degrees in situ fusion in a dysraphic patient with high grade spondylolisthesis" (by M. A. König and B. M. Boszczyk). European spine journal : official publication of the European Spine Society, the European Spinal Deformity Society, and the European Section of the Cervical Spine Research Society. 2012;21(3):396-9.

108. Daviet JC, Bonan I, Caire JM, Colle F, Damamme L, Froger J, et al. Therapeutic patient education for stroke survivors: Non-pharmacological management. A literature review. Annals of physical and rehabilitation medicine. 2012;55(9-10):641-56.

109. Casazza BA. Diagnosis and treatment of acute low back pain. American family physician. 2012;85(4):343-50.

110. Alemdaroğlu E, Uçan H, Topçuoğlu AM, Sivas F. In-hospital predictors of falls in community-dwelling individuals after stroke in the first 6 months after a baseline evaluation: a prospective cohort study. Archives of physical medicine and rehabilitation. 2012;93(12):2244-50.

111. Wennberg AL, Altman D, Lundholm C, Klint A, Iliadou A, Peeker R, et al. Genetic influences are important for most but not all lower urinary tract symptoms: a population-based survey in a cohort of adult Swedish twins. European urology. 2011;59(6):1032-8.

112. Tubaro A, Koelbl H, Laterza R, Khullar V, de Nunzio C. Ultrasound imaging of the pelvic floor: where are we going? Neurourology and urodynamics. 2011;30(5):729-34.

113. Torricelli FC, Lucon M, Vicentini F, Gomes CM, Srougi M, Bruschini H. PSA levels in men with spinal cord injury and under intermittent catheterization. Neurourology and urodynamics. 2011;30(8):1522-4.

114. Teleman P, Persson J. [Removal of TVT sling from urinary bladder. Endoscopy in simpler cases, robot-assisted laparoscopy in more complicated cases]. Lakartidningen. 2011;108(41):2030-2.

115. Petros P. The integral system. Central European journal of urology. 2011;64(3):110-9.

116. Lucchetti G, Granero AL. Use of comprehensive geriatric assessment in general practice: results from the 'Senta Pua' project in Brazil. The European journal of general practice. 2011;17(1):20-7.

117. Lee CY, Chen LK, Lo YK, Liang CK, Chou MY, Lo CC, et al. Urinary incontinence: an under-recognized risk factor for falls among elderly dementia patients. Neurourology and urodynamics. 2011;30(7):1286-90.

118. Härlein J, Halfens RJ, Dassen T, Lahmann NA. Falls in older hospital inpatients and the effect of cognitive impairment: a secondary analysis of prevalence studies. Journal of clinical nursing. 2011;20(1-2):175-83.

119. Gomes T, Juurlink DN, Ho JM, Schneeweiss S, Mamdani MM. Risk of serious falls associated with oxybutynin and tolterodine: a population based study. The Journal of urology. 2011;186(4):1340-4.

120. Edwards R, Martin FC, Grant R, Lowe D, Potter J, Husk J, et al. Is urinary continence considered in the assessment of older people after a fall in England and Wales? Cross-sectional clinical audit results. Maturitas. 2011;69(2):179-83.

121. Divani AA, Majidi S, Barrett AM, Noorbaloochi S, Luft AR. Consequences of stroke in community-dwelling elderly: the health and retirement study, 1998 to 2008. Stroke. 2011;42(7):1821-5.

122. Blanco Lago R, Málaga Diéguez I, Álvarez Caro F. [Lumbar spinal cord injury without radiological abnormality in a child: an exceptional form of presentation]. Archivos argentinos de pediatria. 2011;109(3):e47-51.

123. Arlandis S, Castro D, Errando C, Fernández E, Jiménez M, González P, et al. Cost-effectiveness of sacral neuromodulation compared to botulinum neurotoxin a or continued medical management in refractory overactive bladder. Value in health : the journal of the International Society for Pharmacoeconomics and Outcomes Research. 2011;14(2):219-28.

124. Zhang AJ, Yu XJ, Wang M. The clinical manifestations and pathophysiology of cerebral small vessel disease. Neuroscience bulletin. 2010;26(3):257-64.

125. Yalcin I, Peng G, Viktrup L, Bump RC. Reductions in stress urinary incontinence episodes: what is clinically important for women? Neurourology and urodynamics. 2010;29(3):344-7.

126. Wang J, Chang LH, Eberly LE, Virnig BA, Kane RL. Cognition moderates the relationship between facility characteristics, personal impairments, and nursing home residents' activities of daily living. Journal of the American Geriatrics Society. 2010;58(12):2275-83.

127. van Nieuwenhuizen RC, van Dijk N, van Breda FG, Scheffer AC, Korevaar JC, van der Cammen TJ, et al. Assessing the prevalence of modifiable risk factors in older patients visiting an ED due to a fall using the CAREFALL Triage Instrument. The American journal of emergency medicine. 2010;28(9):994-1001.

128. Spoelstra S, Given B, von Eye A, Given C. Falls in the community-dwelling elderly with a history of cancer. Cancer nursing. 2010;33(2):149-55.

129. Oliver D, Healey F, Haines TP. Preventing falls and fall-related injuries in hospitals. Clinics in geriatric medicine. 2010;26(4):645-92.

130. Messer M. [From case to case: proper management of urinary and fecal incontinence]. Pflege Zeitschrift. 2010;63(10):630-1.

131. Jacklin P, Duckett J, Renganathan A. Analytic model comparing the cost utility of TVT versus duloxetine in women with urinary stress incontinence. International urogynecology journal. 2010;21(8):977-84.

132. Hong GR, Cho SH, Tak Y. Falls among Koreans 45 years of age and older: incidence and risk factors. Journal of advanced nursing. 2010;66(9):2014-24.

133. Hasegawa J, Kuzuya M, Iguchi A. Urinary incontinence and behavioral symptoms are independent risk factors for recurrent and injurious falls, respectively, among residents in long-term care facilities. Archives of gerontology and geriatrics. 2010;50(1):77-81.

134. Gobierno Hernández J, Pérez de Las Casas MO, Madan Pérez MT, Baute Díaz D, Manzaneque Lara C, Domínguez Coello S. [Can we prevent falls in the elderly from primary care?]. Atencion primaria. 2010;42(5):284-91.

135. Finlayson J, Morrison J, Jackson A, Mantry D, Cooper SA. Injuries, falls and accidents among adults with intellectual disabilities. Prospective cohort study. Journal of intellectual disability research : JIDR. 2010;54(11):966-80.

136. Duckett J, Basu M, Papanikolaou N. Transperineal ultrasound to assess the effect of tension-free vaginal tape position on flow rates. Ultrasound in obstetrics & gynecology : the official journal of the International Society of Ultrasound in Obstetrics and Gynecology. 2010;36(3):379-83.

137. Wennberg AL, Molander U, Fall M, Edlund C, Peeker R, Milsom I. A longitudinal population-based survey of urinary incontinence, overactive bladder, and other lower urinary tract symptoms in women. European urology. 2009;55(4):783-91.

138. Wennberg AL, Molander U, Fall M, Edlund C, Peeker R, Milsom I. Lower urinary tract symptoms: lack of change in prevalence and help-seeking behaviour in two population-based surveys of women in 1991 and 2007. BJU international. 2009;104(7):954-9.

139. Stranne J, Damber JE, Fall M, Hammarsten J, Knutson T, Peeker R. One-third of the Swedish male population over 50 years of age suffers from lower urinary tract symptoms. Scandinavian journal of urology and nephrology. 2009;43(3):199-205.

140. Sran MM. Prevalence of urinary incontinence in women with osteoporosis. Journal of obstetrics and gynaecology Canada : JOGC = Journal d'obstetrique et gynecologie du Canada : JOGC. 2009;31(5):434-9.

141. Lucioni A, Kobashi KC. Bone-anchored suburethral sling: surgical technique and outcomes. Current urology reports. 2009;10(5):384-9.

142. Lowenstein L, Pham T, Abbasy S, Kenton K, Brubaker L, Mueller ER, et al. Observations relating to urinary sensation during detrusor overactivity. Neurourology and urodynamics. 2009;28(6):497-500.

143. Lee MJ, Chang CP, Lee YH, Wu YC, Tseng HW, Tung YY, et al. Longitudinal evaluation of an N-ethyl-N-nitrosourea-created murine model with normal pressure hydrocephalus. PloS one. 2009;4(11):e7868.

144. Divani AA, Vazquez G, Barrett AM, Asadollahi M, Luft AR. Risk factors associated with injury attributable to falling among elderly population with history of stroke. Stroke. 2009;40(10):3286-92.

145. Chen YM, Chen LK, Lan JL, Chen DY. Geriatric syndromes in elderly patients with rheumatoid arthritis. Rheumatology (Oxford, England). 2009;48(10):1261-4.

146. Boele van Hensbroek P, van Dijk N, van Breda GF, Scheffer AC, van der Cammen TJ, Lips P, et al. The CAREFALL Triage instrument identifying risk factors for recurrent falls in elderly patients. The American journal of emergency medicine. 2009;27(1):23-36.

147. Araki A, Ito H. Diabetes mellitus and geriatric syndromes. Geriatrics & gerontology international. 2009;9(2):105-14.

148. Safaz I, Alaca R, Yasar E, Tok F, Yilmaz B. Medical complications, physical function and communication skills in patients with traumatic brain injury: a single centre 5-year experience. Brain injury. 2008;22(10):733-9.

149. Rapp K, Lamb SE, Büchele G, Lall R, Lindemann U, Becker C. Prevention of falls in nursing homes: subgroup analyses of a randomized fall prevention trial. Journal of the American Geriatrics Society. 2008;56(6):1092-7.

150. Nelson A, Collins J, Siddharthan K, Matz M, Waters T. Link between safe patient handling and patient outcomes in long-term care. Rehabilitation nursing : the official journal of the Association of Rehabilitation Nurses. 2008;33(1):33-43.

151. Nakagawa Y, Sannomiya K, Kinoshita M, Shiomi T, Okada K, Yokoyama H, et al. Development of an assessment sheet for fall prediction in stroke inpatients in convalescent rehabilitation wards in Japan. Environmental health and preventive medicine. 2008;13(3):138-47.

152. Harvey MA, Johnston SL, Davies GA. Mid-trimester serum relaxin concentrations and post-partum pelvic floor dysfunction. Acta obstetricia et gynecologica Scandinavica. 2008;87(12):1315-21.

153. Groenendijk PM, Lycklama à Nyeholt AA, Heesakkers JP, van Kerrebroeck PE, Hassouna MM, Gajewski JB, et al. Urodynamic evaluation of sacral neuromodulation for urge urinary incontinence. BJU international. 2008;101(3):325-9.

154. Duckett RA, Grapsas P, Eaton M, Basu M. The effect of spinal anaesthesia on urethral function. International urogynecology journal and pelvic floor dysfunction. 2008;19(2):257-60.

155. Dingwall L. Promoting social continence using incontinence management products. British journal of nursing (Mark Allen Publishing). 2008;17(9):s12-9.

156. Delbaere K, Close JC, Menz HB, Cumming RG, Cameron ID, Sambrook PN, et al. Development and validation of fall risk screening tools for use in residential aged care facilities. The Medical journal of Australia. 2008;189(4):193-6.

157. Candel-Parra E, Córcoles-Jiménez MP, Del Egido-Fernández MA, Villada-Munera A, Jiménez-Sánchez MD, Moreno-Moreno M, et al. [Independence in activities of daily living 6 months after surgery in previously independent elderly patients with hip fracture caused by a fall]. Enfermeria clinica. 2008;18(6):309-16.

158. Prevention of falls and fall-related injuries in community-dwelling seniors: an evidence-based analysis. Ontario health technology assessment series. 2008;8(2):1-78.

159. Behavioural interventions for urinary incontinence in community-dwelling seniors: an evidence-based analysis. Ontario health technology assessment series. 2008;8(3):1-52.

160. Caregiver- and patient-directed interventions for dementia: an evidence-based analysis. Ontario health technology assessment series. 2008;8(4):1-98.

161. Social isolation in community-dwelling seniors: an evidence-based analysis. Ontario health technology assessment series. 2008;8(5):1-49.

162. van Kerrebroeck PE, van Voskuilen AC, Heesakkers JP, Lycklama á Nijholt AA, Siegel S, Jonas U, et al. Results of sacral neuromodulation therapy for urinary voiding dysfunction: outcomes of a prospective, worldwide clinical study. The Journal of urology. 2007;178(5):2029-34.

163. Ljungqvist L, Peeker R, Fall M. Female urethral diverticulum: 26-year followup of a large series. The Journal of urology. 2007;177(1):219-24; discussion 24.

164. Falconer C. [Female urinary incontinence is investigated and treated in primary health care. Specialist care in complicated cases--without long waiting time]. Lakartidningen. 2007;104(46):3455-9.

165. Zhang Q, Pang Q, Ge Z. [Internal fixation for pelvic posterior ring lesions]. Zhongguo xiu fu chong jian wai ke za zhi = Zhongguo xiufu chongjian waike zazhi = Chinese journal of reparative and reconstructive surgery. 2006;20(12):1214-6.

166. Swarztrauber K, Graf E, Cheng E. The quality of care delivered to Parkinson's disease patients in the U.S. Pacific Northwest Veterans Health System. BMC neurology. 2006;6:26.

167. Starkman JS, Scarpero H, Dmochowski RR. Methods and results of urethrolysis. Current urology reports. 2006;7(5):384-94.

168. Robinson J. Continence: sizing and fitting a penile sheath. British journal of community nursing. 2006;11(10):420-7.

169. Reeves P, Irwin D, Kelleher C, Milsom I, Kopp Z, Calvert N, et al. The current and future burden and cost of overactive bladder in five European countries. European urology. 2006;50(5):1050-7.

170. Miller AH, Mangione KK. Does delirium need immediate medical referral in a frail, homebound elder? Journal of geriatric physical therapy (2001). 2006;29(2):57-63.

171. Messer M. [From case to case: finding the proper tone--and the best solution]. Pflege Zeitschrift. 2006;59(10):654-5.

172. Mattiasson A, Teleman P. Abnormal urethral motor function is common in female stress, mixed, and urge incontinence. Neurourology and urodynamics. 2006;25(7):703-8.

173. Weber AM, Richter HE. Pelvic organ prolapse. Obstetrics and gynecology. 2005;106(3):615-34.

174. Tannenbaum C, Mayo N, Ducharme F. Older women's health priorities and perceptions of care delivery: results of the WOW health survey. CMAJ : Canadian Medical Association journal = journal de l'Association medicale canadienne. 2005;173(2):153-9.

175. Takazawa K, Arisawa K. Relationship between the type of urinary incontinence and falls among frail elderly women in Japan. The journal of medical investigation : JMI. 2005;52(3-4):165-71.

176. Storch JS. Lumbar burst fracture associated with bowel, bladder, and sexual dysfunction: case study. The Journal of neuroscience nursing : journal of the American Association of Neuroscience Nurses. 2005;37(2):68-71.

177. Mecocci P, von Strauss E, Cherubini A, Ercolani S, Mariani E, Senin U, et al. Cognitive impairment is the major risk factor for development of geriatric syndromes during hospitalization: results from the GIFA study. Dementia and geriatric cognitive disorders. 2005;20(4):262-9.

178. Mancini C, Williamson D, Binkin N, Michieletto F, De Giacomi GV. [Epidemiology of falls among the elderly]. Igiene e sanita pubblica. 2005;61(2):117-32.

179. Krauss MJ, Evanoff B, Hitcho E, Ngugi KE, Dunagan WC, Fischer I, et al. A case-control study of patient, medication, and care-related risk factors for inpatient falls. Journal of general internal medicine. 2005;20(2):116-22.

180. Klay M, Marfyak K. Use of a continence nurse specialist in an extended care facility. Urologic nursing. 2005;25(2):101-2, 7-8.

181. Hu TW, Wagner TH. Health-related consequences of overactive bladder: an economic perspective. BJU international. 2005;96 Suppl 1:43-5.

182. Higashi T, Hays RD, Brown JA, Kamberg CJ, Pham C, Reuben DB, et al. Do proxies reflect patients' health concerns about urinary incontinence and gait problems? Health and quality of life outcomes. 2005;3:75.

183. Chen JS, March LM, Schwarz J, Zochling J, Makaroff J, Sitoh YY, et al. A multivariate regression model predicted falls in residents living in intermediate hostel care. Journal of clinical epidemiology. 2005;58(5):503-8.

184. Britnell SJ, Cole JV, Isherwood L, Sran MM, Britnell N, Burgi S, et al. Postural health in women: the role of physiotherapy. Journal of obstetrics and gynaecology Canada : JOGC = Journal d'obstetrique et gynecologie du Canada : JOGC. 2005;27(5):493-510.

185. Becker C, Loy S, Sander S, Nikolaus T, Rissmann U, Kron M. An algorithm to screen long-term care residents at risk for accidental falls. Aging clinical and experimental research. 2005;17(3):186-92.

186. Balash Y, Peretz C, Leibovich G, Herman T, Hausdorff JM, Giladi N. Falls in outpatients with Parkinson's disease: frequency, impact and identifying factors. Journal of neurology. 2005;252(11):1310-5.

187. Warnke A, Meyer G, Bender R, Mühlhauser I. Predictors of adherence to the use of hip protectors in nursing home residents. Journal of the American Geriatrics Society. 2004;52(3):340-5.

188. Oliver D, Daly F, Martin FC, McMurdo ME. Risk factors and risk assessment tools for falls in hospital in-patients: a systematic review. Age and ageing. 2004;33(2):122-30.

189. Mann E, Koller M, Mann C, van der Cammen T, Steurer J. Comprehensive Geriatric Assessment (CGA) in general practice: results from a pilot study in Vorarlberg, Austria. BMC geriatrics. 2004;4:4.

190. Lindsay R, James EL, Kippen S. The Timed Up and Go Test: unable to predict falls on the acute medical ward. The Australian journal of physiotherapy. 2004;50(4):249-51.

191. Knight GE, Burnstock G. The effect of pregnancy and the oestrus cycle on purinergic and cholinergic responses of the rat urinary bladder. Neuropharmacology. 2004;46(7):1049-56.

192. Kela N, Kela P. [From case to case: silent pain]. Pflege Zeitschrift. 2004;57(10):740-1.

193. Huang HC. A checklist for assessing the risk of falls among the elderly. The journal of nursing research : JNR. 2004;12(2):131-42.

194. Edlund C, Dijkema HE, Hassouna MM, Van Kerrebroeck PE, Peeker R, Van den Hombergh U, et al. Sacral nerve stimulation for refractory urge symptoms in elderly patients. Scandinavian journal of urology and nephrology. 2004;38(2):131-5.

195. Brading A, Pessina F, Esposito L, Symes S. Effects of metabolic stress and ischaemia on the bladder, and the relationship with bladder overactivity. Scandinavian journal of urology and nephrology Supplementum. 2004(215):84-92.

196. Wilson TS, Lemack GE, Zimmern PE. Management of intrinsic sphincteric deficiency in women. The Journal of urology. 2003;169(5):1662-9.

197. Williams JG, Cheung WY, Cohen DR, Hutchings HA, Longo MF, Russell IT. Can randomised trials rely on existing electronic data? A feasibility study to explore the value of routine data in health technology assessment. Health technology assessment (Winchester, England). 2003;7(26):iii, v-x, 1-117.

198. Wennberg AL, Edlund C, Fall M, Peeker R. Stamey's abdominovaginal needle colposuspension for the correction of female genuine stress urinary incontinence--long-term results. Scandinavian journal of urology and nephrology. 2003;37(5):419-23.

199. Pushkar D, Rasner PI. [Radical prostatectomy: surgical techniques and preliminary results]. Urologiia (Moscow, Russia : 1999). 2003(2):12-7.

200. Pils K, Neumann F, Meisner W, Schano W, Vavrovsky G, Van der Cammen TJ. Predictors of falls in elderly people during rehabilitation after hip fracture--who is at risk of a second one? Zeitschrift fur Gerontologie und Geriatrie. 2003;36(1):16-22.

201. Kron M, Loy S, Sturm E, Nikolaus T, Becker C. Risk indicators for falls in institutionalized frail elderly. American journal of epidemiology. 2003;158(7):645-53.

202. Hader C, Welz-Barth A, Keller T. [Urinary incontinence--case report]. Deutsche medizinische Wochenschrift (1946). 2003;128(14):745.

203. Goepel M, Michel MC. [The overactive bladder--a case for the urologist]. Der Urologe Ausg A. 2003;42(6):775.

204. de Rekeneire N, Visser M, Peila R, Nevitt MC, Cauley JA, Tylavsky FA, et al. Is a fall just a fall: correlates of falling in healthy older persons. The Health, Aging and Body Composition Study. Journal of the American Geriatrics Society. 2003;51(6):841-6.

205. Abrams P, Cardozo L, Fall M, Griffiths D, Rosier P, Ulmsten U, et al. The standardisation of terminology in lower urinary tract function: report from the standardisation sub-committee of the International Continence Society. Urology. 2003;61(1):37-49.

206. Wagner TH, Hu TW, Bentkover J, LeBlanc K, Stewart W, Corey R, et al. Health-related consequences of overactive bladder. The American journal of managed care. 2002;8(19 Suppl):S598-607.

207. Tromp E. [Risk profiles and preventive measures of falls in elderly persons]. Tijdschrift voor gerontologie en geriatrie. 2002;33(1):21-5.

208. Peeker R, Edlund C, Wennberg AL, Fall M. The treatment of sphincter incontinence with periurethral silicone implants (macroplastique). Scandinavian journal of urology and nephrology. 2002;36(3):194-8.

209. Ippolito C, Spisani L, De Luca G, Romano A, Daniele C, Cartei F, et al. [Role of transrectal ultrasonography in prostatic brachytherapy]. Archivio italiano di urologia, andrologia : organo ufficiale [di] Societa italiana di ecografia urologica e nefrologica. 2002;74(4):295-8.

210. Tromp AM, Pluijm SM, Smit JH, Deeg DJ, Bouter LM, Lips P. Fall-risk screening test: a prospective study on predictors for falls in community-dwelling elderly. Journal of clinical epidemiology. 2001;54(8):837-44.

211. Sze KH, Wong E, Leung HY, Woo J. Falls among Chinese stroke patients during rehabilitation. Archives of physical medicine and rehabilitation. 2001;82(9):1219-25.

212. Radley SC, Chapple CR, Bryan NP, Clarke DE, Craig DA. Effect of methoxamine on maximum urethral pressure in women with genuine stress incontinence: a placebo-controlled, double-blind crossover study. Neurourology and urodynamics. 2001;20(1):43-52.

213. Knutson T, Edlund C, Fall M, Dahlstrand C. BPH with coexisting overactive bladder dysfunction--an everyday urological dilemma. Neurourology and urodynamics. 2001;20(3):237-47.

214. Janknegt RA, Hassouna MM, Siegel SW, Schmidt RA, Gajewski JB, Rivas DA, et al. Long-term effectiveness of sacral nerve stimulation for refractory urge incontinence. European urology. 2001;39(1):101-6.

215. Edlund C, Peeker R, Fall M. Lidocaine cystometry in the diagnosis of bladder overactivity. Neurourology and urodynamics. 2001;20(2):147-55.

216. Fall M. Discussion: reactivation of bladder inhibitory reflexes-an underestimated asset in the treatment of overactive bladder. Urology. 2000;55(5A Suppl):29-30; discussion 1-2.

217. Edlund C, Hellström M, Peeker R, Fall M. First Scandinavian experience of electrical sacral nerve stimulation in the treatment of the overactive bladder. Scandinavian journal of urology and nephrology. 2000;34(6):366-76.

218. Wilkins K. Medications and fall-related fractures in the elderly. Health reports. 1999;11(1):45-53(Eng); 49-58(Fre).

219. Wehrend A, Funk J. [Case report. Urinary incontinence in a newborn foal]. Tierarztliche Praxis Ausgabe G, Grosstiere/Nutztiere. 1999;27(1):8, 52-3.

220. Gregory SP, Holt PE, Parkinson TJ, Wathes CM. Vaginal position and length in the bitch: relationship to spaying and urinary incontinence. The Journal of small animal practice. 1999;40(4):180-4.

221. Fall M. [Urinary incontinence in men--a neglected problem? A quarter of all 80-year old men suffer of urinary leakage]. Lakartidningen. 1999;96(18):2227-31.

222. Rentzhog L, Stanton SL, Cardozo L, Nelson E, Fall M, Abrams P. Efficacy and safety of tolterodine in patients with detrusor instability: a dose-ranging study. British journal of urology. 1998;81(1):42-8.

223. Madersbacher H, Awad S, Fall M, Janknegt RA, Stöhrer M, Weisner B. Urge incontinence in the elderly--supraspinal reflex incontinence. World journal of urology. 1998;16 Suppl 1:S35-43.

224. Fall M. Advantages and pitfalls of functional electrical stimulation. Acta obstetricia et gynecologica Scandinavica Supplement. 1998;168:16-21.

225. Tutuarima JA, van der Meulen JH, de Haan RJ, van Straten A, Limburg M. Risk factors for falls of hospitalized stroke patients. Stroke. 1997;28(2):297-301.

226. Sylvester J, Blasko JC, Grimm P, Ragde H. Interstitial implantation techniques in prostate cancer. Journal of surgical oncology. 1997;66(1):65-75.

227. Nyberg L, Gustafson Y. Fall prediction index for patients in stroke rehabilitation. Stroke. 1997;28(4):716-21.

228. Méndez Rubio JI, Zunzunegui MV, Béland F. [The prevalence of and factors associated with falls in older persons living in the community]. Medicina clinica. 1997;108(4):128-32.

229. Geirsson G, Fall M. Maximal functional electrical stimulation in routine practice. Neurourology and urodynamics. 1997;16(6):559-65.

230. Brandeis GH, Baumann MM, Hossain M, Morris JN, Resnick NM. The prevalence of potentially remediable urinary incontinence in frail older people: a study using the Minimum Data Set. Journal of the American Geriatrics Society. 1997;45(2):179-84.

231. Meier C, Hess C, Meier-Abt PJ, Krähenbühl S. [A clinical-pharmacological case (1). Action of cisapride (Prepulsid) on bladder function]. Praxis. 1996;85(3):62-4.

232. Hahn I, Milsom I, Ohlsson BL, Ekelund P, Uhlemann C, Fall M. Comparative assessment of pelvic floor function using vaginal cones, vaginal digital palpation and vaginal pressure measurements. Gynecologic and obstetric investigation. 1996;41(4):269-74.

233. Ferro JM, Falcão I, Rodrigues G, Canhão P, Melo TP, Oliveira V, et al. Diagnosis of transient ischemic attack by the nonneurologist. A validation study. Stroke. 1996;27(12):2225-9.

234. Fall M, Geirsson G. Positive ice-water test: a predictor of neurological disease? World journal of urology. 1996;14 Suppl 1:S51-4.

235. Schwenzer T, Beck L. [Pre-vesicle ureteral obstruction as a sequela of Bruch colposuspension for urinary stress incontinence and partial prolapse. A case from the expert assessment files]. Der Gynakologe. 1995;28(4):276-7.

236. Fall M. Vaginal wall bipedicled flap and other techniques in complicated urethral diverticulum and urethrovaginal fistula. Journal of the American College of Surgeons. 1995;180(2):150-6.

237. Chin YK, Stanton SL. A follow up of silastic sling for genuine stress incontinence. British journal of obstetrics and gynaecology. 1995;102(2):143-7.

238. List J, Stendel R, Rudolph KH, Brock M. [A case of diastematomyelia (split cord malformation type I) with clinical manifestation in adulthood]. Zentralblatt fur Neurochirurgie. 1994;55(4):212-7.

239. Fowler CJ, Beck RO, Gerrard S, Betts CD, Fowler CG. Intravesical capsaicin for treatment of detrusor hyperreflexia. Journal of neurology, neurosurgery, and psychiatry. 1994;57(2):169-73.

240. Cervigni M, Perricone C, Panei M. [Echographic diagnosis and treatment of urethral instability]. Archivio italiano di urologia, andrologia : organo ufficiale [di] Societa italiana di ecografia urologica e nefrologica. 1994;66(4 Suppl):225-8.

241. Autret E, Jonville AP, Dutertre JP, Bertiere MC, Robert M, Averous M, et al. Plasma levels of oxybutynine chloride in children. European journal of clinical pharmacology. 1994;46(1):83-5.

242. Wise BG, Cardozo LD, Cutner A, Benness CJ, Burton G. Prevalence and significance of urethral instability in women with detrusor instability. British journal of urology. 1993;72(1):26-9.

243. Schnizer W. [Therapeutic muscle training]. Wiener klinische Wochenschrift. 1993;105(8):232-8.

244. Ramsay IN, Hilton P, Cox TF. Time-series analysis of urethral electrical conductance measurements in the assessment of unstable urethral pressure: results in normal patients and in those with genuine stress incontinence. Neurourology and urodynamics. 1993;12(1):23-31.

245. Petros PE, Ulmsten U. Bladder instability in women: a premature activation of the micturition reflex. Neurourology and urodynamics. 1993;12(3):235-9.

246. Petros PE, Ulmsten U. Natural volume handwashing urethrocystometry: a physiological technique for the objective diagnosis of the unstable detrusor. Gynecologic and obstetric investigation. 1993;36(1):42-6.

247. Hahn I, Milsom I, Fall M, Ekelund P. Long-term results of pelvic floor training in female stress urinary incontinence. British journal of urology. 1993;72(4):421-7.

248. Geirsson G, Fall M, Lindström S. Subtypes of overactive bladder in old age. Age and ageing. 1993;22(2):125-31.

249. Geirsson G, Fall M, Lindström S. The ice-water test--a simple and valuable supplement to routine cystometry. British journal of urology. 1993;71(6):681-5.

250. Boccafoschi C, Annoscia S, Lozzi C, Signorello D. [Vesico-urethral and entero-urethral anastomosis: anatomo-surgical considerations and technical note]. Archivio italiano di urologia, andrologia : organo ufficiale [di] Societa italiana di ecografia urologica e nefrologica. 1993;65(5):563-9.

251. Milsom I, Fall M, Ekelund P. [Urinary incontinence--an expensive national disease]. Lakartidningen. 1992;89(20):1772-4.

252. Versi E, Cardozo L, Anand D, Cooper D. Symptoms analysis for the diagnosis of genuine stress incontinence. British journal of obstetrics and gynaecology. 1991;98(8):815-9.

253. Simeonova Z, Bengtsson C, Ekelund P, Milsom I. [Urinary incontinence and other urogenital problems in women--cases for primary health care?]. Lakartidningen. 1991;88(50):4329-31.

254. Fall M, Lindström S. Electrical stimulation. A physiologic approach to the treatment of urinary incontinence. The Urologic clinics of North America. 1991;18(2):393-407.

255. DuBeau CE, Resnick NM. Evaluation of the causes and severity of geriatric incontinence. A critical appraisal. The Urologic clinics of North America. 1991;18(2):243-56.

256. Werbrouck P, Baert L, Binard JE, Chiou RK, Van Poppel H. Balloon dilatation of the external urethral sphincter: a case study. The Journal of the American Paraplegia Society. 1990;13(2):13-4.

257. Fernandes CE, Morita MH, Ferreira JA, da Silva EP, Wehba S. [Approach to urinary tract disorders in postmenopausal women]. Revista paulista de medicina. 1990;108(5):230-5.

258. Tapp A, Fall M, Norgaard J, Massey A, Choa R, Carr T, et al. Terodiline: a dose titrated, multicenter study of the treatment of idiopathic detrusor instability in women. The Journal of urology. 1989;142(4):1027-31.

259. Low JA, Armstrong JB, Mauger GM. The unstable urethra in the female. Obstetrics and gynecology. 1989;74(1):69-74.

260. Kohorn EI. The surgery of stress urinary incontinence. Obstetrics and gynecology clinics of North America. 1989;16(4):841-52.

261. Holt PE. Urethral pressure profilometry in the anaesthetised bitch: a comparison between double and single sensor recording. Research in veterinary science. 1989;47(3):346-9.

262. Holmes DM, Plevnik S, Stanton SL. Bladder neck electrical conductivity in the treatment of detrusor instability with biofeedback. British journal of obstetrics and gynaecology. 1989;96(7):821-6.

263. Anderström CR, Fall M, Johansson SL. Scanning electron microscopic findings in interstitial cystitis. British journal of urology. 1989;63(3):270-5.

264. Sannoh N, Kubokura T, Nishimura T, Koyama S, Tsubone K. [A case of spontaneous cervical epidural hematoma associated with cervical spondylosis]. No shinkei geka Neurological surgery. 1988;16(5 Suppl):539-43.

265. Richter K. [Surgical anatomy of the bladder neck sphincter and its significance for vaginal surgery of stress incontinence]. Geburtshilfe und Frauenheilkunde. 1988;48(8):541-50.

266. Kindermann G, Debus-Thiede G. Postoperative urological complications after radical surgery for cervical cancer. Bailliere's clinical obstetrics and gynaecology. 1988;2(4):933-41.

267. Ohlsson B, Lindström S, Erlandson BE, Fall M. Effects of some different pulse parameters on bladder inhibition and urethral closure during intravaginal electrical stimulation: an experimental study in the cat. Medical & biological engineering & computing. 1986;24(1):27-33.

268. Fall M, Ahlstrom K, Carlsson CA, Ek A, Erlandson BE, Frankenberg S, et al. Contelle: pelvic floor stimulator for female stress-urge incontinence. A multicenter study. Urology. 1986;27(3):282-7.

269. Lewis J. Bedside diagnosis of urinary incontinence. Canadian family physician Medecin de famille canadien. 1985;31:2319-22.

270. Fall M, Johansson SL, Vahlne A. A clinicopathological and virological study of interstitial cystitis. The Journal of urology. 1985;133(5):771-3.

271. Fall M, Frankenberg S, Frisén M, Larsson B, Petrén M. [456,000 Swedes may have urinary incontinence. Only every fourth person seeks help for the disorder]. Lakartidningen. 1985;82(22):2054-6.

272. Fall M, Erlandson BE, Pettersson S. Evaluation of history and simple supine cystometry as a preoperative test in stress urinary incontinence. Acta obstetricia et gynecologica Scandinavica. 1984;63(3):241-4.

273. Fall M. Does electrostimulation cure urinary incontinence? The Journal of urology. 1984;131(4):664-7.

274. Badr GG, Fall M, Carlsson CA, Lindström L, Friberg S, Ohlsson B. Cortical evoked potentials obtained after stimulation of the lower urinary tract. The Journal of urology. 1984;131(2):306-9.

275. Lindström S, Fall M, Carlsson CA, Erlandson BE. The neurophysiological basis of bladder inhibition in response to intravaginal electrical stimulation. The Journal of urology. 1983;129(2):405-10.

276. Fall M, Pettersson S. The simplified Lapides' operation for stress incontinence. Scandinavian journal of urology and nephrology. 1983;17(1):27-30.

277. Wild D, Nayak US, Isaacs B. Prognosis of falls in old people at home. Journal of epidemiology and community health. 1981;35(3):200-4.

278. Carlsson CA, Erlandson BE, Fall M. [Electrostimulation in disorders of the voiding mechanism of the bladder]. Lakartidningen. 1979;76(12):1085-7.

279. Fall M, Erlandson BE, Carlsson CA, Sundin T. Effects of electrical intravaginal stimulation on bladder volume: an experimental and clinical study. Urologia internationalis. 1978;33(6):440-2.

280. Low JA. Urethral behavior during the involuntary detrusor contraction. American journal of obstetrics and gynecology. 1977;128(1):32-42.

281. Fall M, Erlandson BE, Sundin T, Waagstein F. Intravaginal electrical stimulation. Clinical experiments on bladder inhibition. Scandinavian journal of urology and nephrology Supplementum. 1977(44):41-7.

282. Fall M, Erlandson BE, Nilson AE, Sundin T. Long-term intravaginal electrical stimulation in urge and stress incontinence. Scandinavian journal of urology and nephrology Supplementum. 1977(44):55-63.

283. Erlandson BE, Fall M, Sundin T. Intravaginal electrical stimulation. Clinical experiments of urethral closure. Scandinavian journal of urology and nephrology Supplementum. 1977(44):31-9.

284. Erlandson BE, Fall M, Carlsson CA, Linder LE. Mechanisms for closure of the human urethra during intravaginal electrical stimulation. Scandinavian journal of urology and nephrology Supplementum. 1977(44):49-54.

285. Erlandson BE, Fall M, Carlsson CA. The effect of intravaginal electrical stimulation on the feline urethra and urinary bladder. Electrical parameters. Scandinavian journal of urology and nephrology Supplementum. 1977(44):5-18.

286. Brekkan A. [A CASE OF SACRAL AGENESIS]. Fortschritte auf dem Gebiete der Rontgenstrahlen und der Nuklearmedizin. 1964;100:666-8.

**EMBASE: 439 studies**

1. Omae K, Kurita N, Takeshima T, Naganuma T, Takahashi S, Yoshioka T, et al. Significance of Overactive Bladder as a Predictor of Falls in Community Dwelling Older Adults: 1-Year Followup of the Sukagawa Study. The Journal of urology. 2021;205(1):219-25.

2. Zullo AR, Sorial MN, Lee Y, Lary CW, Kiel DP, Berry SD. Predictors of Hip Fracture Despite Treatment with Bisphosphonates among Frail Older Adults. Journal of the American Geriatrics Society. 2020;68(2):256-60.

3. Yura E, Staniorski C, Cohen J, Singal A, Nettey O, Hofer M. 021 Do Prior Anti-incontinence Procedures Influence the Success of Artificial Urinary Sphincter Placement? Journal of Sexual Medicine. 2020;17(1):S10.

4. Yalcintas E, Demirci H, Aykurt Karlibel I, Turkoglu AR, Kasapoglu Aksoy M, Coban S. Geriatric giants in women over 65 years living in a rural area in Turkey. Journal of women & aging. 2020:1-7.

5. Tsai YJ, Yang PY, Yang YC, Lin MR, Wang YW. Prevalence and risk factors of falls among community-dwelling older people: results from three consecutive waves of the national health interview survey in Taiwan. BMC geriatrics. 2020;20(1):529.

6. Towe M, El-Khatib FM, Osman MM, Huynh LM, Carrion R, Ward S, et al. 341 “Doc, if it were you, what would you do?”: A Survey of Men's Health Specialists' Personal Preferences Regarding Treatment Modalities. Journal of Sexual Medicine. 2020;17(1):S88.

7. Thammawongsa P, Laohasiriwong W. Physical, psychosocial determinants and quality of life of elderly in the northeast of Thailand. Indian Journal of Public Health Research and Development. 2020;11(7):634-8.

8. Siegal AR, Huang Z, Gross MD, Mehraban-Far S, Weissbart SJ, Kim JM. Trends of Mesh Utilization for Stress Urinary Incontinence Before and After the 2011 Food and Drug Administration Notification Between FPMRS-Certified and Non-FPMRS-Certified Physicians: A Statewide All-Payer Database Analysis. Urology. 2020.

9. Shin J, Han SH, Choi J, Kim YS, Lee J. Importance of geriatric syndrome screening within 48 hours of hospitalization for identifying readmission risk: A retrospective study in an acute-care hospital. Annals of Geriatric Medicine and Research. 2020;24(2):83-90.

10. Saavedra-Belaunde JA, Kannady C, Clavell-Hernandez J, Wang R. 328 Improvement of Minimal Stress Urinary Incontinence after Mini-Jupette Sling Placement, During Penile Prosthesis Surgery, for Management of Post-Prostatectomy Climacturia. Journal of Sexual Medicine. 2020;17(1):S82-S3.

11. Roggeman S, Weiss JP, Van Laecke E, Vande Walle J, Everaert K, Bower WF. The role of lower urinary tract symptoms in fall risk assessment tools in hospitals: A review. F1000Research. 2020;9.

12. Parnell JM, Akinjogbin T, Duggan M. Evaluating falls using the comprehensive geriatric assessment. Journal of the American Geriatrics Society. 2020;68(SUPPL 1):S23.

13. Paquin MH, Duclos C, Lapierre N, Dubreucq L, Morin M, Meunier J, et al. The effects of a strong desire to void on gait for incontinent and continent older community-dwelling women at risk of falls. Neurourology and Urodynamics. 2020;39(2):642-9.

14. Niznik JD, Li X, Gilliam MA, Hanson L, Thorpe C. Osteoporosis treatment patterns for nursing home residents with dementia. Journal of the American Geriatrics Society. 2020;68(SUPPL 1):S251-S2.

15. Moser D, Russo F, Henry G, Jani K, Macedo G. 136 Male Pre-surgical Incontinence Assessment: What Methods are Actually Being Used? Journal of Sexual Medicine. 2020;17(1):S38-S9.

16. Moser D, Russo F, Henry G, Jani K, Macedo G. 137 What Methods are Actually Being Used to Evaluate Male Incontinence Surgery Outcomes? Journal of Sexual Medicine. 2020;17(1):S39.

17. Moon S, Chung HS, Yu JM, Na HR, Kim SJ, Ko KJ, et al. Impact of urinary incontinence on falls in the older population: 2017 national survey of older Koreans. Archives of Gerontology and Geriatrics. 2020;90.

18. Min K, Park T. 199 Attitude of Women Nurses for Embarrassing Female Sexual and Urinary Symptoms in Korea. Journal of Sexual Medicine. 2020;17(1):S70.

19. Magnuszewski L, Swietek M, Kasiukiewicz A, Kuprjanowicz B, Baczek J, Wojszel ZB. Health, functional and nutritional determinants of falls experienced in the previous year—a cross-sectional study in a geriatric ward. International Journal of Environmental Research and Public Health. 2020;17(13):1-13.

20. Lee K, Davis MA, Marcotte JE, Pressler SJ, Liang J, Gallagher NA, et al. Falls in community-dwelling older adults with heart failure: A retrospective cohort study. Heart and Lung. 2020;49(3):238-50.

21. Kolterer A, Naumann G. 75/f—mixed urinary incontinence: Preparation for the specialist examination: case 21. Gynakologe. 2020;53:116-20.

22. Kolterer A, Naumann G. 41/f—urinary incontinence: Preparation for the specialist examination: case 15. Gynakologe. 2020;53:81-5.

23. Jimbo M, Gopalakrishna A, Ziegelmann M, Viers B, Kohler T. 134 Prevalence of Climacturia in Patients with History of Definitive Therapy for Prostate Cancer. Journal of Sexual Medicine. 2020;17(1):S38.

24. Janßen U, Baumann O, Friedrich M. 67-year-old female patient with urinary incontinence: Preparation for the medical specialist examination: part 3. Gynakologe. 2020;53:12-8.

25. Isbell T, Des Bordes J, Murdock C, Siddiqui G, Rianon N. Diuretic use is not associated with falls in older women with urinary incontinence: Preliminary data. Journal of the American Geriatrics Society. 2020;68(SUPPL 1):S289.

26. Hentzen C, Villaumé A, Turmel N, Chesnel C, Le Breton F, Haddad R, et al. Are falls in people with multiple sclerosis related to the severity of urinary disorders? Annals of Physical and Rehabilitation Medicine. 2020.

27. Gibbons C, Holt S, Braffett B, Agochukwu N, Sarma A, Wessells H. 198 Associations Between Domains of Urinary Symptoms and Female Sexual Dysfunction in Women with Type 1 Diabetes. Journal of Sexual Medicine. 2020;17(1):S69-S70.

28. Dokuzlar O, Koc Okudur S, Soysal P, Kocyigit SE, Yavuz I, Smith L, et al. Factors that Increase Risk of Falling in Older Men according to Four Different Clinical Methods. Experimental aging research. 2020;46(1):83-92.

29. Dokuzlar O, Koc Okudur S, Smith L, Soysal P, Yavuz I, Aydin AE, et al. Assessment of factors that increase risk of falling in older women by four different clinical methods. Aging Clinical and Experimental Research. 2020;32(3):483-90.

30. Córcoles-Jiménez MP, Candel-Parra E, del Egido-Fernández MÁ, Villada-Munera A, Moreno-Moreno M, Piña-Martínez AJ, et al. Preventing Functional Urinary Incontinence in Hip-Fractured Older Adults Through Patient Education: A Randomized Controlled Trial. Journal of Applied Gerontology. 2020.

31. Christine B, Graziano C, Barrett-Harlow B. 340 Telesurgery: A Modern Take on Historical Surgical Education. Journal of Sexual Medicine. 2020;17(1):S88.

32. Cho ST, Moon S, Kim SJ, Ko KJ, Choi DK, Kwon O, et al. The association between urinary incontinence and falls: A systematic review and meta-analysis. Journal of Urology. 2020;203:e154.

33. Chen PL, Lin HY, Ong JR, Ma HP. Development of a fall-risk assessment profile for community-dwelling older adults by using the National Health Interview Survey in Taiwan. BMC public health. 2020;20(1):234.

34. Carolan A, Zell M, Rose K, Wolter C. 330 Same-day Artificial Urinary Sphincter and Inflatable Penile Prosthesis Placement: Comparable Perioperative Outcomes. Journal of Sexual Medicine. 2020;17(1):S83-S4.

35. Britting S, Artzi-Medvedik R, Fabbietti P, Tap L, Mattace-Raso F, Corsonello A, et al. Kidney function and other factors and their association with falls : The screening for CKD among older people across Europe (SCOPE) study. BMC geriatrics. 2020;20:320.

36. Baumann O, Janßen U, Friedrich M. 48/f—urinary incontinence: Preparation for the specialist examination: part 8. Gynakologe. 2020;53:44-52.

37. Backman WD, Emonds E, Williams P, Joseph J, Orkaby A. Implementation of embedded geriatrics consultation in a heart failure clinic. Journal of the American Geriatrics Society. 2020;68(SUPPL 1):S112.

38. Aranyavalai T, Jalayondeja C, Jalayondeja W, Pichaiyongwongdee S, Kaewkungwal J, Laskin JJ. Association between walking 5000 step/day and fall incidence over six months in urban community-dwelling older people. BMC geriatrics. 2020;20(1):194.

39. Abell JG, Lassale C, Batty GD, Zaninotto P. Risk factors for hospital admission after a fall: a prospective cohort study of community-dwelling older people. The journals of gerontology Series A, Biological sciences and medical sciences. 2020.

40. Abbs E, Brown R, Guzman D, Kaplan L, Kushel M. Risk Factors for Falls in Older Adults Experiencing Homelessness: Results from the HOPE HOME Cohort Study. Journal of General Internal Medicine. 2020;35(6):1813-20.

41. Ziegelmann M, Farrell MR, Levine L. A Modified-approach for Placement of a “Mini-sling” at the Time of Inflatable Penile Prosthesis Implantation for Post-prostatectomy Climacturia and Mild Stress Urinary Incontinence. Journal of Sexual Medicine. 2019;16(4):S141.

42. Zhang N, Zhu W, Liu X, Chen W, Zhu M, Sun X, et al. Related factors of sarcopenia in hospitalized elderly patients with coronary heart disease. Chinese Journal of Cardiology. 2019;47(12):979-84.

43. Yuvarani G, Rajalaxmi V, Kamatchi K, Tharani G, Muthulakshmi K, Sumaiya Banu I. Combination of otago exercises and pelvic floor muscle strengthening exercise AID in avoiding urinary incontinence and fall risk among women. Research Journal of Pharmacy and Technology. 2019;12(3):1105-10.

44. Yi Y, Shakir N, Reddy R, Fuchs J, McKibben M, Morey A. Moderate Male Stress Incontinence: Discordance Between Patient Symptoms and Physical Findings. Journal of Sexual Medicine. 2019;16(4):S52-S3.

45. Thapa S, Shmerling RH, Bean JF, Cai Y, Leveille SG. Chronic multisite pain: evaluation of a new geriatric syndrome. Aging Clinical and Experimental Research. 2019;31(8):1129-37.

46. Tan MP, Mat S, Alex D, Kamaruzzaman SB. Falls in Malaysia: Magnitude, risk, disparities, and solutions. Age and Ageing. 2019;48(2):iv34.

47. Takeuchi T, Yajima K. Long-term 4 years follow-up study of 482 patients who underwent shunting for idiopathic normal pressure hydrocephalus -course of symptoms and shunt efficacy rates compared by age group. Neurologia Medico-Chirurgica. 2019;59(7):281-6.

48. Sun CY, Yang DC, Chang YT, Lin WH, Guo HR, Lin CY, et al. Frail phenotype versus EGFR in predicting longitudinal outcomes for elderly with chronic kidney disease. Aging Medicine and Healthcare. 2019;10:17-8.

49. Smith CJ, Kirkman BJ, Allred DB. Acute worsening hemiparesis in a patient with motor neuron disease, due to kernohan's notch phenomenon of evolving subdural hematoma: A case report. PM and R. 2019;11:S22.

50. Singal A, Staniorski C, Nettey O, Keeter MK, Hairston J, Hofer M. Comparison of Artificial Urethral Sphincter (AUS) Placement Through the Perineal and Trans-scrotal Approach. Journal of Sexual Medicine. 2019;16(4):S88-S9.

51. Schluter PJ, Arnold EP, Jamieson HA. Re: Falls and hip fractures associated with urinary incontinence among older men and women with complex needs: A national population study. Journal of Urology. 2019;202(1):9-10.

52. Rikard C, Bagga B, Dobish E. Catatonia: A unique manifestation of systemic lupus erythematosus. Journal of Investigative Medicine. 2019;67(2):475-6.

53. Raheem O. Comparison of Complication Rates Related to Penile Prosthesis and Advance™ Male Slings Versus Penile Prosthesis and Artificial Urinary Sphincters: National Multi-Institutional Analysis of NSQIP Database. Journal of Sexual Medicine. 2019;16(4):S118-S9.

54. Punjani N, Chan G, Chan E, Abed H, Campbell J, Brock G. Single Perineal Incision for Artificial Urinary Sphincter: An Analysis of Technique, Outcomes, and Experience. Journal of Sexual Medicine. 2019;16(4):S121-S2.

55. Peeters G, Cooper R, Tooth L, van Schoor NM, Kenny RA. A comprehensive assessment of risk factors for falls in middle-aged adults: co-ordinated analyses of cohort studies in four countries. Osteoporosis International. 2019;30(10):2099-117.

56. Oshiro CES, Frankland TB, Rosales AG, Perrin NA, Bell CL, Lo SHY, et al. Fall Ascertainment and Development of a Risk Prediction Model Using Electronic Medical Records. Journal of the American Geriatrics Society. 2019.

57. Nino Soseliya N, Lukina O, Bagmanova N, Babaeva L, Villevalde S, Kobalava Z. Manifestations of frailty in elderly patients with acute coronary syndrome. European Journal of Heart Failure. 2019;21:273.

58. Najafpour Z, Godarzi Z, Arab M, Yaseri M. Risk Factors for Falls in Hospital In-Patients: A Prospective Nested Case Control Study. International journal of health policy and management. 2019;8(5):300-6.

59. Lucas J, Patel A, Loh-Doyle J, Nikolavsky D, Angulo J, Martins F, et al. The Natural History of Patients with Artificial Urinary Sphincter Cuff Erosion. Journal of Sexual Medicine. 2019;16(4):S51.

60. Loh-Doyle J, Thompson E, Hartman N, Boyd S. Outcomes of Dual Inflatable Penile Prosthesis and Artificial Urinary Sphincter Implantation After Radical Cystectomy and Neobladder. Journal of Sexual Medicine. 2019;16(4):S99.

61. Lee I, Walker JB, Nozaki K, Willett LL. Weak and Winded. New England Journal of Medicine. 2019;381(1):76-82.

62. Kranz KA, Abeles R, Schwartz DA, Lane S, Ng P. With her-women's health education for internal medicine residents. Using the jigsaw teaching method for an innovative geriatric women's health curriculum. Journal of the American Geriatrics Society. 2019;67:S229.

63. Killian O, Power D. A case of evolving gerstmann's syndrome with aphasia. Irish Journal of Medical Science. 2019;188:S35-S6.

64. Khovasova N, Moroz V, Demenok D, Balaeva M, Naumov A. Screening of geriatric syndromes in patients with falls. European Geriatric Medicine. 2019;10:S90-S1.

65. Kent M, Say R, Bortnick E, Cavallo J, Valenzuela R. ‘Mini Male Urethral Sling’ and Inflatable Penile Prosthesis for the Treatment of Erectile Dysfunction with Climacturia. Journal of Sexual Medicine. 2019;16(4):S143.

66. Huang MH, Blackwood J, Godoshian M, Pfalzer L. Predictors of falls in older survivors of breast and prostate cancer: A retrospective cohort study of surveillance, epidemiology and end results—Medicare health outcomes survey linkage. Journal of Geriatric Oncology. 2019;10(1):89-97.

67. Griebling TL, Schluter PJ, Arnold EP, Jamieson HA. Re: Falls and hip fractures associated with urinary incontinence among older men and women with complex needs: A national population study. Journal of Urology. 2019;202(1):9-10.

68. Graziano C, McCraw C, Christine B. “But I have always left in a catheter!”: Implantation of the AMS 800 Artificial Urinary Sphincter without Post-op Catheterization. Journal of Sexual Medicine. 2019;16(4):S53.

69. Gogia B, Patel N, Hamouda D, Kovalev D, Shanina E. Elderly onset of neuromyelitis optica with atypical presentation. Annals of Neurology. 2019;86:S37.

70. Gimat R, Dai S, Faletto D, Clarac E, Davoine P, Chrispin A, et al. Fall risk after a stroke: Mean follow-up of 2 years. Neurophysiologie Clinique. 2019;49(6):424.

71. Gibson W, Makhani A, Wagg A, Hunter K. Do older adults with oab demonstrate impaired executive function compared to their peers without OAB? Neurourology and Urodynamics. 2019;38:S289-S91.

72. Escobar-Aguilar G, Moreno-Casbas MT, González-María E, Martínez-Gimeno ML, Sánchez-Pablo C, Orts-Cortés I. The SUMAMOS EXCELENCIA Project. Journal of advanced nursing. 2019;75(7):1575-84.

73. Eglseer D, Hödl M, Lohrmann C. Six Nursing Care Problems in Hospitals: A Cross-Sectional Study of Quality of Care. Journal of nursing care quality. 2019;34(1):E8-E14.

74. Dwyer L, Weaver E, Rajai A, Cox S, Reid FM. “voice your choice”: A multi-centre study of factors determining women's choice of surgery for primary stress urinary incontinence. Female Pelvic Medicine and Reconstructive Surgery. 2019;25(5):S94-S5.

75. Dietrich P, Guise A. Treatment of Stress Urinary Incontinence with Mini-Jupette Sling Placement at the Time of Inflatable Penile Prosthesis Placement: A Case Report. Journal of Sexual Medicine. 2019;16(4):S101.

76. Deseatnicova E, Soric G, Agachi S, Negara A, Groppa L. Quality of life and geriatric syndromes in elderly people in the republic of Moldova. Osteoporosis International. 2019;30(SUPPL 2):S737-S8.

77. Chu CM, Schmitz KH, Khanijow K, Stambakio H, Newman DK, Arya LA, et al. Feasibility and outcomes: Pilot Randomized Controlled Trial of a home-based integrated physical exercise and bladder-training program vs usual care for community-dwelling older women with urinary incontinence. Neurourology and Urodynamics. 2019;38(5):1399-408.

78. Carter A, Smith H, Shridharani A. Simultaneous Placement of Virtue Male Sling and Titan Inflatable Penile Prosthesis in Men Following Radical Prostatectomy. Journal of Sexual Medicine. 2019;16(4):S91.

79. Bugge C, Hay-Smith J, Grant A, Taylor A, Hagen S, McClurg D, et al. A 24 month longitudinal qualitative study of women's experience of electromyography biofeedback pelvic floor muscle training (PFMT) and pfmt alone for urinary incontinence: Adherence, outcome and context. Neurourology and Urodynamics. 2019;38:S347-S8.

80. Beilan J, Slongo J, Baumgarten A, Wiegand L. Single-Incision Urethral Closure and Penile Prosthesis Removal. Journal of Sexual Medicine. 2019;16(4):S120.

81. Baker R, VanDyke M, Yi Y, Davenport M, Bergeson R, Morey A. Long-Term Outcomes of Permanent Urethral Ligation (PUL) for Men with Recurrent Artificial Urinary Sphincter (AUS) Erosion. Journal of Sexual Medicine. 2019;16(4):S123-S4.

82. Abbs E, Brown R, Guzman D, Ponath C, Kaplan LM, Kushel M. Novel risk factors for falls in older adults experiencing homelessness: Results from the hope home cohort study. Journal of General Internal Medicine. 2019;34(2):S294.

83. Yafi FA, Andrianne R, Brady J, Chevalier D, DeLay KJ, Faix A, et al. “andrianne Mini-Jupette” graft at the time of inflatable penile prosthesis placement for the management of post-prostatectomy climacturia and minimal urinary i ncontinenc. Journal of Sexual Medicine. 2018;15(2):S3.

84. Viers BR, Pagliara TJ, VanDyke ME, Shakir NA, Scott JM, Morey AF. Refining male sling selectivity improves outcomes: Role of standing cough test in physical demonstration of mild male stress urinary incontinence. Journal of Sexual Medicine. 2018;15(2):S60.

85. Vallet A, Lokossou A, Lorthois S, Swider P, Assemat P, Risser L, et al. Biomechanical approach of brain aging, neurodegenerative diseases and frailty. Fluids and Barriers of the CNS. 2018;15.

86. Valderrama-Hinds LM, Snih SA, Chen NW, Rodriguez MA, Wong R. Falls in mexican older adults aged 60 years and older. Aging Clinical and Experimental Research. 2018;30(11):1345-51.

87. Tatum PE, Talebreza S, Ross JS. Geriatric assessment: An office-based approach. American Family Physician. 2018;97(12):776-84, 84A-84D.

88. Steinsiek N, Morgan BL, Sajadi KP, Marshall LM. Urinary incontinence and low bone mineral density among older u. S. adults. Neurourology and Urodynamics. 2018;37:S634-S5.

89. Steinsiek N, Morgan BL, Marshall LM, Sajadi KP. Urinary incontinence and low bone mineral density among older united states adults. Journal of Urology. 2018;199(4):e505.

90. Smith EM, Shah AA. Screening for Geriatric Syndromes: Falls, Urinary/Fecal Incontinence, and Osteoporosis. Clinics in Geriatric Medicine. 2018;34(1):55-67.

91. Simonson MG. Failing to consider a fall: Anchoring on recent procedures. Diagnosis. 2018;5(4):eA154.

92. Shapiro RE, Duenas OF, Phillips K, McCrory J, Mancinelli C. Assessing static and dynamic postural stability in women with stress urinary incontinence. Female Pelvic Medicine and Reconstructive Surgery. 2018;24(5):S120-S1.

93. Schluter PJ, Arnold EP, Jamieson HA. Falls and hip fractures associated with urinary incontinence among older men and women with complex needs: A national population study. Neurourology and Urodynamics. 2018;37(4):1336-43.

94. Saka B, Akin S, Kocatürk C, Tasci I, Savas S, Atli T, et al. Measurement of quality of care in elderly in Turkish Hospitals using LPZ tool. European Geriatric Medicine. 2018;9:S206-S7.

95. Sabourin JC, Cameron B, Sanaee MS, Koenig NA, Lee T, Geoffrion R. Pelvic floor hypertonicity in women with pelvic floor disorders: A case control and risk prediction study. Neurourology and Urodynamics. 2018;37:S224-S5.

96. Roberts R, Schlesinger P, Farrukh A. An audit of vaginal tape exposure rates following TVT-O sling procedures: A comparison of 3 tapes. BJOG: An International Journal of Obstetrics and Gynaecology. 2018;125:178.

97. Paquin M, Duclos C, Dubreucq L, Lapierre N, Rousseau J, Meunier J, et al. Best in category prize “geriatrics / gerontology”: Impact of a strong desire to void on gait in continent and incontinent community-dwelling older women who have experienced fall in the last year. Neurourology and Urodynamics. 2018;37:S321-S2.

98. Oura M, Komuro A, Konno D, Hosoi T, Ishii S, Shibasaki K, et al. Association between urinary incontinence and other geriatric syndromes. Journal of the American Geriatrics Society. 2018;66:S320-S1.

99. Ooi GK, De Silva S, Firdaus AM, Lim SW, Zuki Z. Diffuse large B-cell lymphoma mimicking as malignant nerve sheath tumour: A rare case report. Malaysian Orthopaedic Journal. 2018;12.

100. Onder G, Giovannini S, Sganga F, Manes-Gravina E, Topinkova E, Finne-Soveri H, et al. Interactions between drugs and geriatric syndromes in nursing home and home care: results from Shelter and IBenC projects. Aging Clinical and Experimental Research. 2018;30(9):1015-21.

101. Nieto-Riveiro L, Groba B, Miranda MC, Concheiro P, Pazos A, Pousada T, et al. Technologies for participatory medicine and health promotion in the elderly population. Medicine (United States). 2018;97(20).

102. Lin RJ, Elko TA, Hilden P, Dahi PB, Jakubowski AA, Perales MA, et al. Burden and impact of geriatric syndromes associated with allogeneic hematopoietic cell transplantation in older adults. Blood. 2018;132.

103. Liang Y, Rausch C, Laflamme L, Möller J. Prevalence, trend and contributing factors of geriatric syndromes among older Swedes: results from the Stockholm County Council Public Health Surveys. BMC geriatrics. 2018;18(1):322.

104. Lequin M. Imaging of anorectal malformations, with focus on MRI. Pediatric Radiology. 2018;48:S378-S9.

105. Lee K, Davis MA, Marcotte JE, Liang J, Pressler SJ, Gallagher NA, et al. Falls in community-dwelling older adults with heart failure. Journal of the American Geriatrics Society. 2018;66:S320.

106. Kim HJ, Kim JW, Jang SN, Kim KD, Yoo JI, Ha YC. Urinary incontinences are related with fall and fragility fractures in elderly population: Nationwide cohort study. Journal of Bone Metabolism. 2018;25(4):267-74.

107. Kaler MS. Gabapentin induced urinary incontinence-a rare side effect. Journal of the American Geriatrics Society. 2018;66:S179.

108. Jorn HKS. Urinary incontinence after a fall while anticoagulated: Effect of a hematoma in an unlikely location. Journal of General Internal Medicine. 2018;33(2):657-8.

109. Joice G, Srivastava A, Patel H, Manka M, Sopko N, Wright J. Artificial urinary sphincter revision for recurrent incontinence: Single component replacement is both feasible and efficacious. Journal of Sexual Medicine. 2018;15(2):S60.

110. Jiang CX, Zou M, Jiang LP. Characteristic analysis of 590 older adults with geriatric syndromes in Shanghai based on cluster analysis. Journal of Shanghai Jiaotong University (Medical Science). 2018;38(9):1072-8.

111. Jayadevappa R, Chhatre S, Newman DK, Schwartz JS, Wein AJ. Association between overactive bladder treatment and falls among older adults. Neurourology and Urodynamics. 2018;37(8):2688-94.

112. Hehemann M, Choe S, McVary K, Podlasek C. Cavernous nerve crush injury increases apoptosis in a time dependent manner in the entire pelvic plexus. Journal of Sexual Medicine. 2018;15(2):S48.

113. Hehemann M, Choe S, Dynda DA, Alanee S, Hu WY, McVary K, et al. Sonic hedgehog regulation of human rhabdosphinc muscle. Journal of Sexual Medicine. 2018;15(2):S48-S9.

114. Han B, Cotton B, Polydorou S, Blaum C, McNeely J, Sherman S. Geriatric conditions among middle-aged and older adults on methadone maintenance treatment. Journal of the American Geriatrics Society. 2018;66:S237.

115. Haghir E, Moraros J, Feng C, Bird Y. Risk factors associated with single and recurrent falls among community-dwelling elderly in Canada. European Geriatric Medicine. 2018;9:S172.

116. Gaudet D, Clohosey DG, Goldstein SW, Szell N, Komisaruk BR, Hannan JL, et al. Simulation of mid-urethral incontinence sling surgery using cadavers: Impact on innervation and glandular tissue. Journal of Sexual Medicine. 2018;15(2):S45-S6.

117. Frassineti J, Ciocchini ME, Nanni M, Mantovani P, Pirina A, Sturiale C, et al. Missed nursing care and hydrocephalus: Is a good operation enough? Fluids and Barriers of the CNS. 2018;15.

118. Elghblawi E. Platelet-rich plasma, the ultimate secret for youthful skin elixir and hair growth triggering. Journal of Cosmetic Dermatology. 2018;17(3):423-30.

119. Dunlap K, Gill B, Zampini A, Baker D. Preoperative group education and preparedness for prostatectomy among men with prostate cancer. Journal of Sexual Medicine. 2018;15(2):S51-S2.

120. Desjardins A, Randazzo D, Chandramohan V, Peters K, Johnson M, Thomas L, et al. Dose escalation trial of D2C7 immunotoxin (D2C7-IT) administered intratumorally via convection-enhanced delivery (CED) for recurrent malignant glioma (MG). Neuro-Oncology. 2018;20:vi9.

121. Bidair M. The water study sexual function results-a phase iii blinded randomized parallel group trial of aquablation vs. transurethral resection of the prostate with blinded outcome assessment for moderate-to-severe luts in men with benign prostatic hyperplasia. Journal of Sexual Medicine. 2018;15(2):S58-S9.

122. Aliperti L, Patil D, Filson C, Carney K, Sanda M, Mehta A. Nationwide utilization of reconstructive urological procedures to mitigate sexual and urinary complications following radical prostat ectomy. Journal of Sexual Medicine. 2018;15(2):S91.

123. Agudelo-Botero M, Giraldo-Rodríguez L, Murillo-González JC, Mino-León D, Cruz-Arenas E. Factors associated with occasional and recurrent falls in Mexican community-dwelling older people. PLoS ONE. 2018;13(2).

124. Yu WC, Chou MY, Peng LN, Lin YT, Liang CK, Chen LK. Synergistic effects of cognitive impairment on physical disability in all-cause mortality among men aged 80 years and over: Results from longitudinal older veterans study. PLoS ONE. 2017;12(7).

125. Yafi FA, DeLay KJ, Stewart C, Chiang J, Sangkum P, Hellstrom WJ. Device survival following primary implantation of the AMS 800 artificial urinary sphincter for male stress urinary incontinence. Journal of Sexual Medicine. 2017;14(2):e3-e4.

126. Wallen JJ, Madiraju SK, Tayon KG, Gross MS, Carrion RE, Perito PE. The efficacy of durasphere as a new agent for the treatment of hypermobile glans. Journal of Sexual Medicine. 2017;14(2):e24.

127. Toh Q, Kuo TL, Teo JS, Wong TH, Ng JC, Ng LG. A review of genitourinary injuries in a tertiary trauma centre in Singapore. BJU International. 2017;119:38.

128. Talarska D, Strugała M, Szewczyczak M, Tobis S, Michalak M, Wróblewska I, et al. Is independence of older adults safe considering the risk of falls? BMC geriatrics. 2017;17(1):66.

129. Szell N, Qu H, Shaw M, Goldstein SW, Komisaruk BR, Rubin RS, et al. Anterior vaginal wall periurethral tissue: An extensive literature review and metaanalysis of orgasmic and overall sexual function post mid-urethral sling surgery. Journal of Sexual Medicine. 2017;14(2):e93-e4.

130. Szell N, Komisaruk BR, Goldstein SW, Campbell TG, Goldstein I. Review of the evidence of the female prostate as a functional, sexually-relevant gland in some women. Journal of Sexual Medicine. 2017;14(2):e101-e2.

131. Sugimoto T, Yoshida M, Ono R, Murata S, Saji N, Niida S, et al. Frontal Lobe Function Correlates with One-Year Incidence of Urinary Incontinence in Elderly with Alzheimer Disease. Journal of Alzheimer's Disease. 2017;56(2):567-74.

132. Song DG, Tan GK, Coddington N, Brander C, Henry GD. Case report: Worsening male stress urinary incontinence and delayed urethral erosion of the bulking agent after intraurethral injection of macroplastique. Journal of Sexual Medicine. 2017;14(2):e48-e9.

133. Solis GR, Champion JD. Examining Fall Recurrence Risk of Homebound Hispanic Older Adults Receiving Home Care Services. Hispanic health care international : the official journal of the National Association of Hispanic Nurses. 2017;15(1):20-6.

134. Singh BP, Masrukin S, Jabul R, Phung ST. Pathological fracture of bilateral neck of femur with underlying fanconi syndrome: Red flags in management: A case report. Malaysian Orthopaedic Journal. 2017;11.

135. Reyes-Ortiz CA, Pacheco S, Davis AS, Ocampo Chaparro JM. Prevalence and risk factors for falls causing injury among elders in Latin America. Journal of the American Geriatrics Society. 2017;65:S50.

136. Park DS, Lee SR. Bladder training can be helpful for continence recovery in early postprostatectomy phase. Journal of Sexual Medicine. 2017;14(2):e47-e8.

137. Negredo E, Loste C, Saiz M, Puig J, Muñoz-Moreno JA, Lemos B, et al. Aging in HIV-infected and uninfected populations: A comprehensive geriatric assessment. Antiviral Therapy. 2017;22:A36.

138. Mooventhan A, Nivethitha L. Evidence based effects of yoga practice on various health related problems of elderly people: A review. Journal of Bodywork and Movement Therapies. 2017;21(4):1028-32.

139. Mizoguchi A, Sato K, Utsunomiya S, Mimata H, Mori K, Mizoguchi S. A study into the nursing care of institutionalized elderly women with dementia and urinary incontinence, the quality of their sleep, and daytime activeness. Neurourology and Urodynamics. 2017;36:S157-S8.

140. Mitsui K, Park HJ, Park NC, Kim JH. Clinical effectiveness of Xhold® for male urinary incontinence. Journal of Sexual Medicine. 2017;14(2):e48.

141. Locatelli I, Monod S, Cornuz J, Büla CJ, Senn N. A prospective study assessing agreement and reliability of a geriatric evaluation. BMC geriatrics. 2017;17(1):153.

142. Liang CK, Chou MY, Chen LY, Wang KY, Lin SY, Chen LK, et al. Delaying cognitive and physical decline through multidomain interventions for residents with mild-to-moderate dementia in dementia care units in Taiwan: A prospective cohort study. Geriatrics and Gerontology International. 2017;17:36-43.

143. Leshem A, Groutz A, Shimonov M. Effects of bariatric surgery on female pelvic floor disorders: Short versus medium term outcome results. Neurourology and Urodynamics. 2017;36:S205-S6.

144. Kucukdagli P, Ozturk GB, Kiliç C, Erdogan TO, Yilmaz O, Topcu Y, et al. Frailty and chronic pain: A novel association. European Geriatric Medicine. 2017;8:S102-S3.

145. Kotsani M, Soulis G, Achimastos A, Georgiopoulos I. Association of urinary incontinence with geriatric syndromes in community-dwelling highly functional people aged 65 and over. European Geriatric Medicine. 2017;8:S240.

146. Kiliç C, Demir R, Bahat G, Karan MA. Association between fall in the previous year with some comprehensive geriatric assessment components. European Geriatric Medicine. 2017;8:S97.

147. Kehoe S, Johansson T, Sheehy S, Doyle S. Falls and fracture characteristics of femoral fracture patients who present to an orthopaedic unit. Irish Journal of Medical Science. 2017;186(6):S273.

148. Hung CH, Wang CJ, Tang TC, Chen LY, Peng LN, Hsiao FY, et al. Recurrent falls and its risk factors among older men living in the veterans retirement communities: A cross-sectional study. Archives of Gerontology and Geriatrics. 2017;70:214-8.

149. Henderson EJ, Smith N, Gaunt DM, Lawrence AD, Brodie MA, Close JCT, et al. Does the anticholinergic burden of drugs predict outcomes in people with parkinson's disease with a history of a fall? Age and Ageing. 2017;46:i44.

150. Hamilton R. Incontinence and sexual functionality a problem? Journal of Sexual Medicine. 2017;14(5):e282.

151. Hamilton R. Sexual dysfunction: Primary, secondary or a by-product of secret issues? Sexually Transmitted Infections. 2017;93:A92-A3.

152. Gupta G, Kung J. Thyrotoxic periodic paralysis with urinary incontinence. Thyroid. 2017;27:A10.

153. Fournier M, Gratton M, Mouajou V, Richard PO, Tu L. Retrospective pilot study comparing treatment outcomes of patients with and without uninhibited bladder contraction following trigonal nerve ablation via radiofrequency energy. Neurourology and Urodynamics. 2017;36:S423-S4.

154. Elliott S, Zarowski C, Dayan M, Pollock P, Spillane M, Mahovlich S, et al. The utilization of a dedicated sexual health and pelvic floor physiotherapy service for urinary incontinence and erectile dysfunction after prostate cancer diagnosis. Journal of Sexual Medicine. 2017;14(2):e4-e5.

155. Dahodwala N, Nwadiogbu C, Fitts W, Partridge H, Karlawish J. Parkinsonian signs are a risk factor for falls. Gait and Posture. 2017;55:1-5.

156. Curtiss NL, Duckett J, Basu M. Posterior tibial nerve stimulation for overactive bladder in a non-trial situation. International Urogynecology Journal. 2017;28(1):S135.

157. Christine B, Bella A. Climacturia following radical prostatectomy: The time is now to query and treat. Journal of Sexual Medicine. 2017;14(2):e47.

158. Basilius J, Myers JB, Brant WO. Incontinence rates after pressure regulating balloon exchange in patients with artificial urinary sphincters: A single center review. Journal of Sexual Medicine. 2017;14(2):e100.

159. Balachandran A, Duckett J. Cystodistension: Is there evidence to support its use in current practice for patients with overactive bladder? Journal of Obstetrics and Gynaecology. 2017;37(6):700-3.

160. Amuthavalli Thiyagarajan J. Closing the evidence to practice gap: Who ICOPE guideline recommendations. Osteoporosis International. 2017;28(1):S84-S5.

161. Allen C, Zarowitz BJ, O'Shea T, Datto C, Olufade T. Clinical and functional characteristics of nursing facility residents with opioid-induced constipation. Consultant Pharmacist. 2017;32(5):285-98.

162. 2016 Annual Fall Scientific Meeting of SMSNA. Journal of Sexual Medicine. 2017;14(2).

163. Zhang D, He Y, Liu M, Yang H, Wu L, Wang J, et al. Study on incidence and risk factors of fall in the elderly in a rural community in Beijing. Chinese Journal of Endemiology. 2016;37(5):624-8.

164. Yafi FA, Peak T, Mitchell G, Sangkum P, Hellstrom WJG. Synchronous dual AUS/IPP insertion through a single penoscrotal incision. Journal of Sexual Medicine. 2016;13(5):S71.

165. Van Houten P. Urinary incontinence in the elderly can be treated. Nederlands Tijdschrift voor Geneeskunde. 2016;160(17).

166. Tkacheva ON, Runikhina NK, Ostapenko VS, Sharashkina NV, Mkhitaryan EA, Onuchina YS. The prevalence of geriatric syndroms among patients in Moscow outpatient setting. European Geriatric Medicine. 2016;7:S114.

167. Sakushima K, Yamazaki S, Fukuma S, Hayashino Y, Yabe I, Fukuhara S, et al. Influence of urinary urgency and other urinary disturbances on falls in Parkinson's disease. Journal of the Neurological Sciences. 2016;360:153-7.

168. Sacco E, Bientinesi R, Gandi C. Objectively improving appropriateness of pads prescription to patients with urinary incontinence: A pad test-based study. Neurourology and Urodynamics. 2016;35:S460-S2.

169. Pinkas J, Gujski M, Humeniuk E, Raczkiewicz D, Bejga P, Owoc A, et al. State of health and quality of life of women at advanced age. Medical Science Monitor. 2016;22:3095-105.

170. Pahwa AK, Andy UU, Newman DK, Stambakio H, Schmitz KH, Arya LA. Noctural enuresis as a risk factor for falls in older community dwelling women with urinary incontinence. Journal of Urology. 2016;195(5):1512-6.

171. Nguyen MT, Cohen SA, Mei JY, Ackerman AL, Oliver J, Kreydin EI. Preliminary report on the of the vastus lateralis-fascia lata graft for repair of anterior vaginal wall prolapse. Female Pelvic Medicine and Reconstructive Surgery. 2016;22(5):S149.

172. Neville CE. Bladder control problems in elders: Assessment and treatment strategies for all rehabilitation clinicians-clinical commentary. Topics in Geriatric Rehabilitation. 2016;32(4):231-50.

173. Nelson C, Mulhall J. Erectile function and urinary bother following radical prostatectomy (RP). Journal of Sexual Medicine. 2016;13(5):S31-S2.

174. Mariyanovski V, Mladenov B, Kazalakova K. Transobturator Tape (TOT) for stress urinary incontinence - A reliable and safe primary intervention or after failed physiotherapy/kinesitherapy. European Urology, Supplements. 2016;15(10):e1331-e2.

175. Mancini M, Righetto M, Dal Moro F, Zattoni F. First autoptic studies on vesico-vaginal fistulas: Discovery of aetiopathogenesis due to female masturbation. European Urology, Supplements. 2016;15(3):e375.

176. Lukaszyk C, Harvey L, Sherrington C, Keay L, Tiedemann A, Coombes J, et al. Risk factors, incidence, consequences and prevention strategies for falls and fall-injury within older indigenous populations: a systematic review. Australian and New Zealand journal of public health. 2016;40(6):564-8.

177. Kinback NC. Normal pressure hydrocephalus associated with toxic minocycline findings: A case report. PM and R. 2016;8(9):S236.

178. Ishida M, Akrour R, Lang P, Schurch B. Management of the elderly's urinary incontinence preliminary study of the assessment of needs in acute geriatrics service. Neurourology and Urodynamics. 2016;35:S197-S8.

179. Heiry M, Cortez M. Autonomic dysfunction in dementia with lewy bodies, a case series. Neurology. 2016;86(16).

180. Gürler H, Bayraktar N, Erdil F. Effectiveness of falling prevention program for elderly people undergoing fracture treatment in Turkey. European Geriatric Medicine. 2016;7:S135.

181. Gibson W, Wagg A. Are older women more likely to receive surgical treatment for stress urinary incontinence since the introduction of the mid-urethral sling? An examination of Hospital Episode Statistics data. BJOG: An International Journal of Obstetrics and Gynaecology. 2016;123(8):1386-92.

182. Denys M, Decalf V, Bruneel E, Kumps C, Velghe A, Petrovic M, et al. Exploring nocturia in an older population. Neurourology and Urodynamics. 2016;35:S227-S8.

183. Del Deo F, Grimaldi A, Iervolino SA, Pezzella M, Passaretta A, Altamura F, et al. Efficacy of an orally administered combination of hyaluronic acid, chondroitin sulfate, curcumin and quercetin for the prevention of recurrent urinary tract infections in postmenopausal women. Neurourology and Urodynamics. 2016;35:S33-S4.

184. Clarkson B, Griffiths D, Tyagi S, Resnick N. Assessment of repeatability of functional MRI protocol to assess brain activity during urgency. Neurourology and Urodynamics. 2016;35:S319-S20.

185. Brown J, King J. Age-stratified trends in 20 years of stress incontinence surgery in Australia. Australian and New Zealand Journal of Obstetrics and Gynaecology. 2016;56(2):192-8.

186. Barker K, Sran M, Briffa K, Simmonds J. The role of physiotherapy in the management of osteoporosis & bone health. Manual Therapy. 2016;25:e18-e9.

187. Yuh B, Sevilla C, Ginsberg D. An argument for the dynamic state of neurogenic bladder in the cervical level spinal cord injury patient. Neurourology and Urodynamics. 2015;34:S42-S3.

188. Weinberg A, Deibert C, Hernendez K, Valenzuela R. Inflatable penile prosthesis after quadratic transobturator male sling procedure. Journal of Sexual Medicine. 2015;12:155.

189. Weinberg A, Deibert C, Hernendez K, Valenzuela R. Simultaneous inflatable penile prosthesis and quadratic transobturator male sling procedure. Journal of Sexual Medicine. 2015;12:140.

190. Udell JE, Drahota A, Dean TP, Sander R, Mackenzie H. Interventions for preventing falls in older people: An overview of Cochrane Reviews. Cochrane Database of Systematic Reviews. 2015;2015(1).

191. Tannenbaum C, van den Heuvel E, Fritel X, Southall K, Jutai J, Rajabali S, et al. Continence Across Continents To Upend Stigma and Dependency (CACTUS-D): Study protocol for a cluster randomized controlled trial. Trials. 2015;16(1).

192. Semiz M, Kucuk A, Ugur Uslu A, Balkarli A, Sahin M, Can B, et al. Sleep disturbances in patients with Sjogren syndrome. Klinik Psikofarmakoloji Bulteni. 2015;25:S190.

193. Rehman R, Chodankar R. Unusual case of multiple sclerosis (MS) in pregnancy. BJOG: An International Journal of Obstetrics and Gynaecology. 2015;122:262.

194. Raval AD, Zhou S, Wei W, Bhattacharjee S, Miao R, Sambamoorthi U. 30-Day Readmission among Elderly Medicare Beneficiaries with Type 2 Diabetes. Population Health Management. 2015;18(4):256-64.

195. Raup VT, Eswara JR, Geminiani J, Brandes SB. Artificial urinary sphincter for treatment of incontinence in the elderly. Journal of Sexual Medicine. 2015;12:142.

196. Rankin-Wagenaar R, Durbin-Johnson B, Dall'era M, Valicenti R, Shindel A. Pre-treatment counseling on quality of life issues before management of prostate malignancy. Journal of Sexual Medicine. 2015;12:131-2.

197. Prada GI, Popescu MB, Nacu RM, Fita IG, Ignat IA, Andruta DM, et al. Comorbidities in osteoarticular diseases in older people. European Geriatric Medicine. 2015;6:S63.

198. Paich K, Dunn R, Skolarus T, Montie J, Palapattu G, Wood D, et al. Acceptance of a pre-operative educational seminar about recovery from side-effects after prostate cancer surgery. Journal of Sexual Medicine. 2015;12:116.

199. Lima CA, Soares WJ, Bilton TL, Dias RC, Ferrioll E, Perracini MR. Correlates of excessive daytime sleepiness in community-dwelling older adults: an exploratory study. Revista brasileira de epidemiologia = Brazilian journal of epidemiology. 2015;18(3):607-17.

200. Lamloum M, Boudokhane M, Ben Salem T, Ben Nasr M, Said F, Hamzaoui A, et al. Geriatric profile of diabetes in the elderly. European Geriatric Medicine. 2015;6:S128.

201. Kim KH. The role of primary care of voiding dysfunction in rehabilitation and convalescent hospitals. Journal of the Korean Medical Association. 2015;58(6):557-62.

202. Flora RF. The modified equilibrium pressure point theory of stress urinary incontinence. Journal of Minimally Invasive Gynecology. 2015;22(3):S28-S9.

203. Edwards R, Hunter K, Wagg A. Lower urinary tract symptoms and falls in older women: A case control study. Maturitas. 2015;80(3):308-11.

204. Dinsmoor J, Smith MP. Hickam's dictumor occam's razor. Journal of General Internal Medicine. 2015;30:S407.

205. Diabaté I, Sow I, Bâ A, Fall V. Indications of urological surgery by vagina route: A series of 30 cases. African Journal of Urology. 2015;21(1):30-5.

206. Ashangari C, Suleman A. A comprehensive study on postural orthostatic tachycardia syndrome (POTS) symptoms. Autonomic Neuroscience: Basic and Clinical. 2015;192:123.

207. Ajmera M, Raval A, Zhou S, Wei W, Bhattacharya R, Pan C, et al. A real-world observational study of time to treatment intensification among elderly patients with inadequately controlled type 2 diabetes mellitus. Journal of Managed Care Pharmacy. 2015;21(12):1184-93.

208. Abreu HC, Reiners AA, Azevedo RC, Silva AM, Abreu D, Oliveira A. Incidence and predicting factors of falls of older inpatients. Revista de saude publica. 2015;49:37.

209. Zhu ML, Zhou XL, Liu XH. The geriatric syndromes should be valued in china: An investigation in very old Beijing community senior citizens. Journal of the American Geriatrics Society. 2014;62:S369.

210. Zhu M, Liu X, Lin K, Wu J. The neglected geriatric syndromes in the eldest people in beijing communities. Journal of the American Geriatrics Society. 2014;62:S60.

211. Shore ND, Bono P, Massard C, Snapir A, Sarapohja T, Fizazi K. ODM-201 and the CNS: A clinical perspective. Journal of Clinical Oncology. 2014;32(4).

212. Sarigül M, Özel S, Delialioʇlu SÜ, Köklü K. Assessment of balance and falls in geriatric patients and determining risk factors. Clinical and Experimental Rheumatology. 2014;32(4):S93.

213. Sammels M, Vandesande J, Vlaeyen E, Peerlinck K, Milisen K. Falling and fall risk factors in adults with haemophilia: An exploratory study. Haemophilia. 2014.

214. Richardson ML, Sokol ER. A cost effectiveness analysis of conservative versus surgical management for the initial treatment of stress urinary incontinence. Journal of Minimally Invasive Gynecology. 2014;21(2):S8-S9.

215. Rafiq M, McGovern A, Jones S, Harris K, Tomson C, Gallagher H, et al. Falls in the elderly were predicted opportunistically using a decision tree and systematically using a database-driven screening tool. Journal of Clinical Epidemiology. 2014;67(8):877-86.

216. Rachdi I, Lamloum M, Said F, Ben Salem T, Hamzaoui A, Khanfir M, et al. Gerontologic assessment of fragility in elderly: About 162 cases. European Geriatric Medicine. 2014;5:S240-S1.

217. Rachdi I, Lamloum M, Ben Ghorbel I, Ben Salem T, Said F, Hamzaoui A, et al. Polymedication and fragility in elderly: About 162 cases. European Geriatric Medicine. 2014;5:S250.

218. Mani A, Dunning K, Alwell K, Moomaw CJ, Kleindorfer DO, Woo D, et al. Falls with injury after stroke are associated with incontinence and depression. Stroke. 2014;45.

219. Lino VT, Portela MC, Camacho LA, Rodrigues NC. Reliability of screening tests for health-related problems among low-income elderly. Cadernos de saúde pública. 2014;30(12):2691-6.

220. Lim SC, Mamun K, Lim JKH. Comparison between elderly inpatient fallers with and without dementia. Singapore Medical Journal. 2014;55(2):67-71.

221. Liang CK, Chou MY, Peng LN, Liao MC, Chu CL, Lin YT, et al. Gait speed and risk assessment for falls among men aged 80 years and older: A prospective cohort study in Taiwan. European Geriatric Medicine. 2014;5(5):298-302.

222. Lake AMG, Moalli P, E. Richter H, Kim HY, Nager CW, Sirls L, et al. Comparison of ROC curves for preoperative vlpp, MUCP and urinary NTX as predictors for midurethralsling outcomes. Neurourology and Urodynamics. 2014;33(2):166.

223. Lake A, Moalli P, Richter H, Kim HY, Nager C, Sirls L, et al. Comparison of roc curves for preoperative VLPP, MUCP and urinary NTX as predictors for midurethral sling outcomes. Journal of Urology. 2014;191(4):e405.

224. Greene S, Cogan N, Briggs R, Coughlan T, O'Neill D, Mc Cabe D, et al. Neuromedical sequelae post-stroke. Irish Journal of Medical Science. 2014;183(7):S332.

225. Greene M, Valcour V, Miao Y, Covinsky K, Madamba J, Mattes M, et al. Geriatric syndromes are common among older HIV-infected adults. Topics in Antiviral Medicine. 2014;22:392-3.

226. Greene M, Covinsky K, Deeks S, Miao Y, Madamba J, Mattes M, et al. Geriatric syndromes in older HIV-infected adults. Journal of the American Geriatrics Society. 2014;62:S11.

227. Grande E, Guillen-Ponce C, De Prado JMV, Reboredo M, Duran G, Sureda BM, et al. Regorafenib as a single agent for first-line treatment of frail and/or unfit for polychemotherapy patients with metastatic colorectal cancer (mCRC): A study of the Spanish Cooperative Group for digestive tumor therapy (TTD). Journal of Clinical Oncology. 2014;32(15).

228. Gokce A, Sandoval V, Tan RBW, Hellstrom WJ. Long-term outcomes and potential risk factors of artificial urinary sphincter placement for urinary incontinence after radical prostatectomy: A single surgeon, single center experience. Journal of Sexual Medicine. 2014;11:191.

229. Freemantle N, Khalaf K, Loveman C, Stanisic S, Gultyaev D, Lister J, et al. Cost-utility analysis of onabotulinumtoxinA versus best supportive care in the treatment of idiopathic overactive bladder with urinary incontinence among patients not adequately managed with anticholinergic therapy. European Urology, Supplements. 2014;13(1):e701.

230. Figler BD, Gore JL, Rivara FP, Holt SK, Voelzke BB, Wessells H. Diagnosis and treatment of post-prostatectomy erectile dysfunction and urinary incontinence in young privately insured patients, 2007-2010. Journal of Sexual Medicine. 2014;11:152.

231. Dellaroza MSG, Pimenta CAM, Lebrão ML, Duarte YAO, Braga PE. Association between chronic pain and self-reported falls in the SABE study population. Cadernos de Saude Publica. 2014;30(3):522-32.

232. Cofield SS, Salter AR, Tyry T, Tang Y, Yang H, Onyenwenyi A, et al. Multiple sclerosis-associated bladder dysfunction in the NARCOMS registry: A 5-year follow up study. Multiple Sclerosis. 2014;20(1):418.

233. Canning CG, Allen NE, Bloem BR, Keus SH, Munneke M, Nieuwboer A, et al. Interventions for prevention of falls in people with Parkinson's disease: A protocol for a systematic review. Movement Disorders. 2014;29:S229.

234. Bresee C, Dubina ED, Khan AA, Sevilla C, Grant D, Eilber KS, et al. Prevalence and correlates of urinary incontinence among older community-dwelling women. Female Pelvic Medicine and Reconstructive Surgery. 2014;20(6):328-33.

235. Borazjani A, Tadesse H, Kember A, Ayenachew F, Goldman HB, Wall L. The post-fistula incontinence severity scale (PFISS): A pictorial questionnaire for assessment of urinary incontinence following successful closure of obstetric fistula. Female Pelvic Medicine and Reconstructive Surgery. 2014;20:S209.

236. Borazjani A, Tadesse H, Kember A, Ayenachew F, Damaser M, Wall L, et al. Application of the 1-hour pad-test and a novel pictorial questionnaire in the assessment of urinary incontinence following successful closure of obstetric vesicovaginal fistula. Neurourology and Urodynamics. 2014;33(6):1030-2.

237. Borazjani A, Tadesse H, Ayenachew F, Goldman HB, Wall L. Assessment of 24-hour frequency in patients with persistent urinary incontinence following successful closure of obstetric vesicovaginal fistula. Female Pelvic Medicine and Reconstructive Surgery. 2014;20:S210.

238. Andreasson A, Fall M, Persson E, Stranne J, Peeker R. High revision rate following artificial urethral sphincter implantation. Scandinavian Journal of Urology. 2014;48(6):544-8.

239. Acosta KR, Cano J, Rosales M, Cuanang J, Ang J, Dejan RJ. Acute deep cerebral venous thrombosis associated with cerebral hemorrhage in an elderly female: A case report. Journal of Thrombosis and Haemostasis. 2014;12:43.

240. Wolf U, Neef R, Schendel K, Presek P. Bedside implementation of clinical pharmacology to meet the specific requirements of the elderly patient in traumatology. Naunyn-Schmiedeberg's Archives of Pharmacology. 2013;386:S92-S3.

241. Samanta SK, Joseph J, Kundu S, Abhishek K. A study on the spectrum of respiratory disorders in the elderly. Lung India. 2013;30:S42-S3.

242. Rogo-Gupta L, Litwin MS, Saigal CS, Anger JT. Trends in the surgical management of stress urinary incontinence among female medicare beneficiaries, 2002-2007. Neurourology and Urodynamics. 2013;32(2):192.

243. Raval A, Wei W, Zhou S, Bhattacharjee S, Miao R, Sambamoorthi U. Prevalence and factors associated with hospital re-admissions among elderly medicare beneficiaries with type 2 diabetes mellitus (T2DM). Diabetes. 2013;62:A67.

244. Püllen R, Laupheimer U, Hermann E. Predictors for falls in elderly hospital patients. European Geriatric Medicine. 2013;4:S65-S6.

245. Orces C. Prevalence and determinants of falls among older adults in Ecuador: An analysis of the SABE I survey. Journal of the American Geriatrics Society. 2013;61:S34.

246. Mwambelo MC, Mdolo TC, Mfune PHK, Allain TJ. Are geriatric giants a problem in older adults living in urban Malawi? Age and Ageing. 2013;42:ii19.

247. Kim H, Suzuki T, Yoshida H, Shimada H, Yamashiro Y, Sudo M, et al. Are gait parameters related to knee pain, urinary incontinence and a history of falls in community-dwelling elderly women? Japanese Journal of Geriatrics. 2013;50(4):528-35.

248. Jojima T, Aso Y. [Attention to the use of oral anti-diabetic medication in older adults with type 2 diabetes]. Nihon rinsho Japanese journal of clinical medicine. 2013;71(11):1987-92.

249. Hussain A, Pansota MS, Rasool M, Tabassum SA, Ahmad I, Saleem MS. Outcome of end-to-end urethroplasty in post-traumatic stricture of posterior urethra. Journal of the College of Physicians and Surgeons Pakistan. 2013;23(4):272-5.

250. Hersh L, Salzman B. Clinical management of urinary incontinence in women. American Family Physician. 2013;87(9):634-40.

251. Giuliano L, Magnano San Lio P, Fatuzzo D, Tata F, Sueri C, Pappalardo I, et al. Nocturnal convulsive events in patients with obstructive sleep apnea syndrome. Bollettino - Lega Italiana contro l'Epilessia. 2013(145):302-4.

252. Erekson EA, Ciarleglio MM, Strohbehn K, Hanissian PD, Bynum JP, Fried TR. Functional disability, compromised mobility and daily urinary incontinence in older women. Female Pelvic Medicine and Reconstructive Surgery. 2013;19:S88-S9.

253. Edwards R, Hunter K, Wagg A. Lower urinary tract symptoms and falls in women: A case control study. Neurourology and Urodynamics. 2013;32(6):825-6.

254. Damián J, Pastor-Barriuso R, Valderrama-Gama E, de Pedro-Cuesta J. Factors associated with falls among older adults living in institutions. 2013. p. 6.

255. Coyne KS, Wein A, Nicholson S, Kvasz M, Chen CI, Milsom I. Comorbidities and personal burden of urgency urinary incontinence: A systematic review. International Journal of Clinical Practice. 2013;67(10):1015-33.

256. Brodaty H, Kurrle S. Physical morbidity in dementia. Alzheimer's and Dementia. 2013;9(4):P310.

257. Batchelor FA, Dow B, Low MA. Do continence management strategies reduce falls? A systematic review. Australasian Journal on Ageing. 2013;32(4):211-6.

258. Bahat G, Bay I, Selcuk Akpinar T, Tufan A, Kilic C, Baskent A, et al. Determinants of falls and/or fear of falls in community dwelling elderly. Osteoporosis International. 2013;24(1):S315-S6.

259. Arnold PM, Teuber J. Marfan syndrome and symptomatic sacral cyst: Report of two cases. Journal of Spinal Cord Medicine. 2013;36(5):499-503.

260. Whitney JC, Lord SR, Close JCT, Jackson SHD. Simple predictors of falls for cognitively impaired residential care dwellers. Age and Ageing. 2012;41:i39.

261. Warner J, Grimsby G, Wolter C. Bladder capacity on preoperative urodynamics may impact outcomes of transobturator male slings. Journal of Sexual Medicine. 2012;9:58.

262. Vivaldi G. Normal pressure hydrocephalus presenting with cerebellar stroke: A case report. PM and R. 2012;4(10):S374-S5.

263. Toba K. [Locomotive syndrome and frailty. Frail elderly]. Clinical calcium. 2012;22(4):13-9.

264. Sakushima K, Yamazaki S, Hayashino Y, Fukuhara S, Yabe I, Sasaki H. Association between falls and urinary disturbance in Parkinson's disease. European Journal of Neurology. 2012;19:324.

265. Rogo-Gupta L, Litwin MS, Saigal CS, Anger JT. Trends in the surgical management of stress urinary incontinence among female medicare beneficiaries, 2002-2007. Female Pelvic Medicine and Reconstructive Surgery. 2012;18(5):S143.

266. Rhee E. Quadratic male sling: An innovative treatment for male stress urinary incontinence in the complicated male. Journal of Sexual Medicine. 2012;9:9.

267. Paolone D, Williams D. Risks of robotic-assisted laparoscopic prostatectomy as presented on hospital websites. Journal of Sexual Medicine. 2012;9:45.

268. Ortiz JR, Efeovbokham T, Stephens-Kelly C, Rohner I, Suh T. Fall reduction in an ace unit-a model for quality improvement. Journal of the American Geriatrics Society. 2012;60:S226.

269. Moret F, Jaccard-Ruedin H, Bula C, Monod S. The high diagnostic yield of an outpatient geriatric clinic. Journal of the American Geriatrics Society. 2012;60:S175.

270. McCool A. Simultaneous virtue male sling and Titan inflatable penile prosthesis: Single center patient satisfaction and surgical technique. Journal of Sexual Medicine. 2012;9:30-1.

271. Lukazewski A, Mikula B, Servi A, Martin B. Evaluation of a web-based tool in screening for medication-related problems in community-dwelling older adults. Consultant Pharmacist. 2012;27(2):106-13.

272. Lamartina C. Expert's comment concerning Grand Rounds case entitled Limited access surgery for 360 degrees in situ fusion in a dysraphic patient with high grade spondylolisthesis (by M. A. König and B. M. Boszczyk). European Spine Journal. 2012;21(3):396-9.

273. Kandil IM, El Hemaly AKM, Mousa LAS, Serour AG. Imaging the internal urethral sphincter (IUS) as described in a novel concept on the patho-physiology of urinary incontinence and vaginal prolapse. International Journal of Gynecology and Obstetrics. 2012;119:S384.

274. Jones L, Sawczuk A, Shin D, Fromer D. Trans-vaginal mesh placement for the surgical treatment of stress urinary incontinence and pelvic organ prolapse: The male sexual experience. Journal of Sexual Medicine. 2012;9:24.

275. Griebling TL. Re: Does better quality of care for falls and urinary incontinence result in better participant-reported outcomes? Journal of Urology. 2012;187(3):967-8.

276. Griebling TL. Re: Association between the geriatric giants of urinary incontinence and falls in older people using data from the leicestershire MRC incontinence study. Journal of Urology. 2012;188(1):225-6.

277. Gomes G, Hallal P, Alves V. Barriers to engagement in physical activity among adults and elderly non-participants of a physical activity intervention in primary health care. Journal of Science and Medicine in Sport. 2012;15:S164.

278. Garcia-Gracia C, Arce K, Tarchini G. Quadriplegia secondary to an epidural tuberculous abscess. Neurology. 2012;78(1).

279. Faouzi M, Wiseem H, Ghassen T, Adnen H, Nejib BS, Adel S, et al. Suburethral compressive strips in the treatment of male post-operative urinary incontinence study about 28 patients describing our modified technique of Invance®. Journal of Endourology. 2012;26:A140.

280. Enemchukwu E, Kaufman M, Whittam B, Milam D. Comorbid erectile dysfunction in men requiring surgical intervention for postprostatectomy urinary incontinence. Journal of Sexual Medicine. 2012;9:47.

281. Enemchukwu E, Kaufman M, Milam D. Presenting symptoms in patients with artificial urinary sphincter cuff erosion. Journal of Sexual Medicine. 2012;9:60.

282. Daviet JC, Bonan I, Caire JM, Colle F, Damamme L, Froger J, et al. Therapeutic patient education for stroke survivors: Non-pharmacological management. A literature review. Annals of Physical and Rehabilitation Medicine. 2012;55(9-10):641-56.

283. Davies R, Lisk R, Yeong K. Audit into fragility hip fractures in local care home residents. Osteoporosis International. 2012;23:S575-S6.

284. Chancellor MB. State-of-the-Art Lecture 2: Clinical development of muscle stem cells for stress urinary incontinence treatment. International Journal of Urology. 2012;19(4):383-4.

285. Alemdarolu E, Uçan H, Topçuolu AM, Sivas F. In-hospital predictors of falls in community-dwelling individuals after stroke in the first 6 months after a baseline evaluation: A prospective cohort study. Archives of Physical Medicine and Rehabilitation. 2012;93(12):2244-50.

286. Zvara P, Ursiny M, Alavian M, Garett Esters M, Blaivas J. Porous bioceramic particles, a new bulking agent for treatment of stress urinary incontinence. International Urogynecology Journal and Pelvic Floor Dysfunction. 2011;22:S1651-S2.

287. Wittmann D, He C, Coelho M, Hollenbeck B, Montie J, Wood D. Patients' pre-operative expectations of urinary and sexual functioning do not match their actual outcomes one year after radical prostatectomy. Journal of Sexual Medicine. 2011;8:33.

288. Wilbanks J. Male sling for the treatment of male stress urinary incontinence (SUI). Journal of Sexual Medicine. 2011;8:35.

289. Wennberg AL, Altman D, Lundholm C, Klint S, Iliadou A, Peeker R, et al. Genetic influences are important for most but not all lower urinary tract symptoms: A population-based survey in a cohort of adult Swedish twins. European Urology. 2011;59(6):1032-8.

290. Tubaro A, Koelbl H, Laterza R, Khullar V, De Nunzio C. Ultrasound Imaging of the Pelvic Floor: Where Are We Going? Neurourology and Urodynamics. 2011;30(5):729-34.

291. Nadaa A, Vittal P, Bhutto J, Reynaud P. When good drugs behave badly. Journal of General Internal Medicine. 2011;26:S374.

292. Morone G, Iosa M, Paolucci S. Patients' walking ability overestimation increases the fall risk in stroke. Gait and Posture. 2011;33:S55.

293. Lucchetti G, Granero AL. Use of comprehensive geriatric assessment in general practice: Results from the 'Senta Pua' project in Brazil. European Journal of General Practice. 2011;17(1):20-7.

294. Lee U, Scott V, Rashid R, Behniwal A, Maliski S, Anger J. Defining OAB: Disagreement among the experts. Neurourology and Urodynamics. 2011;30(2):207-8.

295. Lee CY, Chen LK, Lo YK, Liang CK, Chou MY, Lo CC, et al. Urinary incontinence: An under-recognized risk factor for falls among elderly dementia patients. Neurourology and Urodynamics. 2011;30(7):1286-90.

296. Knoll LD, Christine B. Simultaneous advance male sling and an inflatable penile prosthesis: Concurrent placement does not increase potential for implant infection. Journal of Sexual Medicine. 2011;8:34.

297. Iturrioz I, Olaizola A, Garate MJ, Mondragon G, Arrieta M, Idiaquez M, et al. Belt restraint reduction. a real possibility in a Gerontologic Center. European Geriatric Medicine. 2011;2:S99.

298. Härlein J, Halfens RJG, Dassen T, Lahmann NA. Falls in older hospital inpatients and the effect of cognitive impairment: A secondary analysis of prevalence studies. Journal of Clinical Nursing. 2011;20(1-2):175-83.

299. Gomes T, Juurlink DN, Ho JMW, Schneeweiss S, Mamdani MM. Risk of serious falls associated with oxybutynin and tolterodine: A population based study. Journal of Urology. 2011;186(4):1340-4.

300. Ghannam S, Pinkhasov R, Jhaveri J, Chan S, Lee M, Shabsigh R. Urinary incontinence: What happens in the bedroom and between the sheets? Journal of Sexual Medicine. 2011;8:16.

301. Ghannam S, Jhaveri J, Pinkhasov R, Hakimian P, Lindsay G, Shabsigh R. How common is urinary incontinence among older american men? Journal of Sexual Medicine. 2011;8:46.

302. Fritel X, Lachal L, Cassou B, Fauconnier A, Dargent-Molina P. Relationship between physical capability and urinary incontinence in 1942 women of 75-85 years. Neurourology and Urodynamics. 2011;30(6):1168-9.

303. Edwards R, Martin FC, Grant R, Lowe D, Potter J, Husk J, et al. Is urinary continence considered in the assessment of older people after a fall in England and Wales? Cross-sectional clinical audit results. Maturitas. 2011;69(2):179-83.

304. Divani AA, Majidi S, Barrett AM, Noorbaloochi S, Luft AR. Consequences of stroke in community-dwelling elderly: The health and retirement study, 1998 to 2008. Stroke. 2011;42(7):1821-5.

305. Christine B, Bella A. Post-radical prostatectomy climacturia resolution with treatment of persistent incontinence using the advance male sling. Journal of Sexual Medicine. 2011;8:37.

306. Zhang AJ, Yu XJ, Wang M. The clinical manifestations and pathophysiology of cerebral small vessel disease. Neuroscience Bulletin. 2010;26(3):257-64.

307. Yalcin I, Peng G, Viktrup L, Bump RC. Reductions in stress urinary incontinence episodes: What is clinically important for women? Neurourology and Urodynamics. 2010;29(3):344-7.

308. Wein AJ. Randomized, placebo-controlled trial of the cognitive effect, safety, and tolerability of oral extended-release oxybutynin in cognitively impaired nursing home residents with urge urinary incontinence. Journal of Urology. 2010;184(5):2030-1.

309. Vinsnes AG, Helbostad JL, Nyronning S, Granbo R, Seim A. A general physical training programme for residents in nursing homes and the effect on urinary incontinence: A randomized controlled trial. Neurourology and Urodynamics. 2010;29(6):852-3.

310. Shah H. Outcome of holmium laser enucleation (HoLEP) for large (more than 100 g) symptomatic benign prostatic hyperplasia. Journal of Urology. 2010;183(4):e130.

311. Rosenzweig AB, Hofmann MT. A unique cause of cognitive and functional decline in a 70 year old man presenting with a traumatic fall. Journal of the American Geriatrics Society. 2010;58:S133.

312. Richy FF, Gunn A, Makaroff L, Gervasoni C, Helmers S. Gastrointestinal disorders in patients with Parkinson's disease: A double-edged sword. Movement Disorders. 2010;25:S261.

313. Richy F, Gunn A, Makaroff L, Gervasoni C, Helmers S. Gastrointestinal disorders in patients with Parkinson's disease: A double-edged sword. Value in Health. 2010;13(3):A137.

314. Oliver D, Healey F, Haines TP. Preventing falls and fall-related injuries in Hospitals. Clinics in Geriatric Medicine. 2010;26(4):645-92.

315. Moon H, Kim J, Park S, Kim Y, Park H, Choi H, et al. Influence of overactive bladder on falls: Study on females aged 40 and older in urban and rural communities. Urology. 2010;76(3):S94.

316. McCool A, Steidle C, Brady J, Tu LM, Corcos J. Early clinician experience with the coloplast virtue® male sling for the treatment of male stress urinary incontinence (SUI). Journal of Sexual Medicine. 2010;47:33.

317. Mahajan ST, Patel PB, Marrie RA. Under Treatment of Overactive Bladder Symptoms in Patients With Multiple Sclerosis: An Ancillary Analysis of the NARCOMS Patient Registry. Journal of Urology. 2010;183(4):1432-7.

318. Mahajan ST, Patel PB, Marrie R. The impact of overactive bladder symptoms and treatments on quality of life measures in patients with multiple sclerosis. Journal of Pelvic Medicine and Surgery. 2010;16(2):S28-S9.

319. Hasegawa J, Kuzuya M, Iguchi A. Urinary incontinence and behavioral symptoms are independent risk factors for recurrent and injurious falls, respectively, among residents in long-term care facilities. Archives of Gerontology and Geriatrics. 2010;50(1):77-81.

320. Gobierno Hernández J, Pérez de las Casas MO, Madan Pérez MT, Baute Díaz D, Manzaneque Lara C, Domínguez Coello S. Can we prevent falls in the elderly from primary care? Atencion Primaria. 2010;42(5):284-91.

321. Florent R, Gunn A, Makaroff L, Gervasoni C, Helmers S. Gastrointestinal disorders in patients with Parkinson's disease (PD): A double-edged sword. Movement Disorders. 2010;25:S669-S70.

322. Finlayson J, Morrison J, Jackson A, Mantry D, Cooper SA. Injuries, falls and accidents among adults with intellectual disabilities. Prospective cohort study. Journal of Intellectual Disability Research. 2010;54(11):966-80.

323. Divani AA, Azquez GV, Barrett AM, Baybutt T, Luft AR. Post-stroke falling and injury due to fall among elderly population: Health and retirement study survey 2002-2006. Stroke. 2010;41(4):e235.

324. Anger JT, Scott V, Rashid R, Behniwal A, Litwin M, Maliski S. Defining OAB: Disagreement among the experts. Journal of Pelvic Medicine and Surgery. 2010;16(5):S142-S3.

325. Albaba M. A nonagenarian male with functional decline and urinary urgency: A case of subdural hematoma. Journal of the American Geriatrics Society. 2010;58:S140.

326. Wennberg AL, Molander U, Fall M, Edlund C, Peeker R, Milsom I. Lower urinary tract symptoms: Lack of change in prevalence and help-seeking behaviour in two population-based surveys of women in 1991 and 2007. BJU International. 2009;104(7):954-9.

327. Wennberg AL, Molander U, Fall M, Edlund C, Peeker R, Milsom I. A Longitudinal Population-based Survey of Urinary Incontinence, Overactive Bladder, and Other Lower Urinary Tract Symptoms in Women. European Urology. 2009;55(4):783-91.

328. Sran MM. Prevalence of Urinary Incontinence in Women With Osteoporosis. Journal of Obstetrics and Gynaecology Canada. 2009;31(5):434-9.

329. Mousa L, El Hemaly A, Kandil I, Serour A, El Sheikha K. Menopause: Pelvic organ prolapse (POP) and stress urinary incontinence (SUI). International Journal of Gynecology and Obstetrics. 2009;107:S276-S7.

330. Morris EB, Khan RB, Ledet D, Howell C, Pui CH, Hudson MM, et al. Neurological morbidity in survivors of childhood acute lymphoblastic leukemia (ALL). Annals of Neurology. 2009;66:S103.

331. Morris B, Khan R, Ledet D, Howell C, Pui C, Hudson M, et al. Neurological morbidity in survivors of childhood acute lymphoblastic leukemia (ALL). Journal of Clinical Oncology. 2009;27(15):9529.

332. Lucioni A, Kobashi KC. Bone-anchored suburethral sling: Surgical technique and outcomes. Current Urology Reports. 2009;10(5):384-9.

333. Lee PG, Cigolle C, Blaum C. The co-occurrence of chronic diseases and geriatric syndromes: The health and retirement study. Journal of the American Geriatrics Society. 2009;57(3):511-6.

334. Lee MJ, Chang CP, Lee YH, Wu YC, Tseng HW, Tung YY, et al. Longitudinal evaluation of an N-Ethyl-N-nitrosourea-created murine model with normal pressure hydrocephalus. PLoS ONE. 2009;4(11).

335. Ibrahim M, Mousa L, El Hemaly A, Kandil I, Serour A, El Sheikha K. Menopause: Pelvic organ prolapse (POP) and stress urinary incontinence (SUI). International Journal of Gynecology and Obstetrics. 2009;107:S653-S4.

336. Hunter K, Freund-Heritage R, Chan L, Tilroe S, Lechelt K. Pilot of the ICIQ-OAB in an outreach falls prevention clinic for older adults. Neurourology and Urodynamics. 2009;28(7):714.

337. Hosseini J, Tavakkoli Tabassi K, Razi A. Delayed retropubic urethroplasty of completely transected urethra associated with pelvic fracture in girls. Urology journal. 2009;6(4):272-5.

338. El Hemaly A, Kandil I, Mousa L, Serour A, El Sheikha K. “Urethro-Vaginoplasty” an operation innovated for the treatment of stress urinary incontinence, SUI, and vaginal prolapse. International Journal of Gynecology and Obstetrics. 2009;107:S590-S1.

339. Divani AA, Vazquez G, Barrett AM, Asadollahi M, Luft AR. Risk factors associated with injury attributable to falling among elderly population with history of stroke. Stroke. 2009;40(10):3286-92.

340. Boele van Hensbroek P, van Dijk N, van Breda GF, Scheffer AC, van der Cammen TJ, Lips P, et al. The CAREFALL Triage instrument identifying risk factors for recurrent falls in elderly patients. American Journal of Emergency Medicine. 2009;27(1):23-36.

341. Araki A, Ito H. Diabetes mellitus and geriatric syndromes. Geriatrics and Gerontology International. 2009;9(2):105-14.

342. Rapp K, Lamb SE, Büchele G, Lall R, Lindemann U, Becker C. Prevention of falls in nursing homes: Subgroup analyses of a randomized fall prevention trial. Journal of the American Geriatrics Society. 2008;56(6):1092-7.

343. Nikolaus T. 25 Years of progress in geriatric medicine: How much therapy can be tolerated by the patient? MMW-Fortschritte der Medizin. 2008;150(48):112-3.

344. Nakagawa Y, Sannomiya K, Kinoshita M, Shiomi T, Okada K, Yokoyama H, et al. Development of an assessment sheet for fall prediction in stroke inpatients in convalescent rehabilitation wards in Japan. Environmental Health and Preventive Medicine. 2008;13(3):138-47.

345. Hosseini J, Tavakkoli Tabassi K. Surgical repair of posterior urethral defects: review of literature and presentation of experiences. Urology journal. 2008;5(4):215-22.

346. Harvey MA, Johnston SL, Davies GAL. Mid-trimester serum relaxin concentrations and post-partum pelvic floor dysfunction. Acta Obstetricia et Gynecologica Scandinavica. 2008;87(12):1315-21.

347. Groenendijk PM, Lycklama À Nyeholt AAB, Heesakkers JPFA, Van Kerrebroeck PEV, Hassouna MM, Gajewski JB, et al. Urodynamic evaluation of sacral neuromodulation for urge urinary incontinence. BJU International. 2008;101(3):325-9.

348. Duckett RAJ, Grapsas P, Eaton M, Basu M. The effect of spinal anaesthesia on urethral function. International Urogynecology Journal. 2008;19(2):257-60.

349. Dingwall L. Promoting social continence using incontinence management products. British journal of nursing (Mark Allen Publishing). 2008;17(9):s12-9.

350. Delbaere K, Close JCT, Menz HB, Cumming RG, Cameron ID, Sambrook PN, et al. Development and validation of fall risk screening tools for use in residential aged care facilities. Medical Journal of Australia. 2008;189(4):193-6.

351. Candel-Parra E, Córcoles-Jiménez MP, Del Egido-Fernández MA, Villada-Munera A, Jiménez-Sánchez MD, Moreno-Moreno M, et al. Independence in activities of daily living 6 months after surgery in previously independent elderly patients with hip fracture caused by a fall. Enfermería clínica. 2008;18(6):309-16.

352. Bartodziej U, Szyłło K, Włodarczyk B, Górski J. Analysis of urodynamic study selected parameters as evaluation of lower urinary tract dysfunction following a radical hysterectomy and radiotherapy because of cervical cancer. Przeglad Menopauzalny. 2008;12(6):332-7.

353. Stenzelius K. Editorial Comment on: Association between Physical Activity and Urinary Incontinence in a Community-Based Elderly Population Aged 70 Years and Over. European Urology. 2007;52(3):874-5.

354. Shafik IA, Shafik A. Ischiocavernosus perineorrhaphy for the treatment of stress urinary incontinence: A novel technique. Journal of Gynecologic Surgery. 2007;23(3):105-10.

355. Ljungqvist L, Peeker R, Fall M. Female Urethral Diverticulum: 26-Year Followup of a Large Series. Journal of Urology. 2007;177(1):219-24.

356. Fehrling M, Fall M, Peeker R. Maximal functional electrical stimulation as a single treatment: Is it cost-effective? Scandinavian Journal of Urology and Nephrology. 2007;41(2):132-7.

357. Zhang Q, Pang Q, Ge Z. Internal fixation for pelvic posterior ring lesions. Zhongguo xiu fu chong jian wai ke za zhi = Zhongguo xiufu chongjian waike zazhi = Chinese journal of reparative and reconstructive surgery. 2006;20(12):1214-6.

358. Teo JSH, Briffa NK, Devine A, Dhaliwal SS, Prince RL. Do sleep problems or urinary incontinence predict falls in elderly women? Australian Journal of Physiotherapy. 2006;52(1):19-24.

359. Swarztrauber K, Graf E, Cheng E. The quality of care delivered to Parkinson's disease patients in the U.S. Pacific Northwest Veterans Health System. BMC Neurology. 2006;6.

360. Robinson J. Continence: sizing and fitting a penile sheath. British journal of community nursing. 2006;11(10):420-7.

361. Miller AH, Mangione KK. Does delirium need immediate medical referral in a frail, homebound elder? Journal of Geriatric Physical Therapy. 2006;29(2):57-63.

362. Mattiasson A, Teleman P. Abnormal urethral motor function is common in female stress, mixed, and urge incontinence. Neurourology and Urodynamics. 2006;25(7):703-8.

363. Machtens S, Karstens JH, Baumann R, Jonas U. Interstitial brachytherapy (LDR-Brachytherapy) in the treatment of patients with prostate cancer. European Urology, Supplements. 2006;5(6):514-21.

364. Colombel M, Poissonnier L, Martin X, Gelet A. Clinical results of the prostate HIFU project. European Urology, Supplements. 2006;5(6):491-4.

365. Altman D, Zetterstrom J, Schultz I, Nordenstam J, Hjern F, Lopez A, et al. Pelvic organ prolapse and urinary incontinence in women with surgically managed rectal prolapse: A population-based case-control study. Coloproctology. 2006;28(5):255-63.

366. Wider C, Vingerhoets F, Bogousslavsky J. Gait disorders in the elderly: Physiological and semiological aspects. Schweizer Archiv fur Neurologie und Psychiatrie. 2005;156(2):58-65.

367. Tannenbaum C, Mayo N, Ducharme F. Older women's health priorities and perceptions of care delivery: Results of the WOW health survey. Canadian Medical Association Journal. 2005;173(2):153-9.

368. Takazawa K, Arisawa K. Relationship between the type of urinary incontinence and falls among frail elderly women in Japan. Journal of Medical Investigation. 2005;52(3-4):165-71.

369. Mecocci P, Von Strauss E, Cherubini A, Ercolani S, Mariani E, Senin U, et al. Cognitive impairment is the major risk factor for development of geriatric syndromes during hospitalization: Results from the GIFA study. Dementia and Geriatric Cognitive Disorders. 2005;20(4):262-9.

370. Mancini C, Williamson D, Binkin N, Michieletto F, De Giacomi GV. Epidemiology of falls among the elderly. Igiene e sanità pubblica. 2005;61(2):117-32.

371. Hu TW, Wagner TH. Health-related consequences of overactive bladder: An economic perspective. BJU International. 2005;96(SUPPL. 1):43-5.

372. Higashi T, Hays RD, Brown JA, Kamberg CJ, Pham C, Reuben DB, et al. Do proxies reflect patients' health concerns about urinary incontinence and gait problems? Health and quality of life outcomes. 2005;3.

373. Gallien P, Adrien S, Petrilli S, Durufle A, Robineau S, Kerdoncuff V, et al. Standing at home and quality of life three years after stroke. Annales de Readaptation et de Medecine Physique. 2005;48(5):225-30.

374. Corna LM, Cairney J. The role of social support in the relationship between urinary incontinence and psychological distress in older adults. Canadian Journal on Aging. 2005;24(3):285-94.

375. Becker C, Loy S, Sander S, Nikolaus T, Rißmann U, Kron M. An algorithm to screen long-term care residents at risk for accidental falls. Aging - Clinical and Experimental Research. 2005;17(3):186-92.

376. Balash Y, Peretz C, Leibovich G, Herman T, Hausdorff JM, Giladi N. Falls in outpatients with Parkinson's disease: Frequency, impact and identifying factors. Journal of Neurology. 2005;252(11):1310-5.

377. Warnke A, Meyer G, Bender R, Mühlhauser I. Predictors of Adherence to the Use of Hip Protectors in Nursing Home Residents. Journal of the American Geriatrics Society. 2004;52(3):340-5.

378. Oliver D, Daly F, Martin FC, McMurdo MET. Risk factors and risk assessment tools for falls in hospital in-patients: A systematic review. Age and Ageing. 2004;33(2):122-30.

379. Mann E, Koller M, Mann C, Van Der Cammen T, Steurer J. Comprehensive Geriatric Assessment (CGA) in general practice: Results from a pilot study in Vorarlberg, Austria. BMC Geriatrics. 2004;4:1-10.

380. Kilonzo M, Vale L, Stearns SC, Grant A, Cody J, Glazener CMA, et al. Cost effectiveness of tension-free vaginal tape for the surgical management of female stress incontinence. International Journal of Technology Assessment in Health Care. 2004;20(4):455-63.

381. Fall M. Do objective urodynamic or clinical findings determine impact of urinary incontinence or its treatment on quality of life?: Editorial comment. Urology. 2004;63(1):71-2.

382. Wilson TS, Lemack GE, Zimmern PE. Management of intrinsic sphincteric deficiency in women. Journal of Urology. 2003;169(5):1662-9.

383. Williams JG, Cheung WY, Cohen DR, Hutchings HA, Longo MF, Russell IT. Can randomised trials rely on existing electronic data? A feasibility study to explore the value of routine data in health technology assessment. Health technology assessment (Winchester, England). 2003;7(26):iii, v-x, 1-117.

384. Wennberg AL, Edlund C, Fall M, Peeker R. Stamey's Abdominovaginal Needle Colposuspension for the Correction of Female Genuine Stress Urinary Incontinence: Long-term Results. Scandinavian Journal of Urology and Nephrology. 2003;37(5):419-23.

385. Pushkar DI, Rasner PI. Radical prostatectomy: surgical techniques and preliminary results. Urologiia (Moscow, Russia : 1999). 2003(2):12-7.

386. Pils K, Neumann F, Meisner W, Schano W, Vavrovsky G, Van der Cammen TJM. Predictors of falls in elderly people during rehabilitation after hip fracture - Who is at risk of a second one? Zeitschrift fur Gerontologie und Geriatrie. 2003;36(1):16-22.

387. Luber KM. Risks, comorbidities, complications and quality of life in patients with urinary incontinence. Director (Cincinnati, Ohio). 2003;11(4):166, 9-72; quiz 73.

388. Kron M, Loy S, Sturm E, Nikolaus T, Becker C. Risk indicators for falls in institutionalized frail elderly. American Journal of Epidemiology. 2003;158(7):645-53.

389. Hader C, Welz-Barth A, Keller T. Urinary incontinence - Case report. Deutsche Medizinische Wochenschrift. 2003;128(14):745.

390. De Rekeneire N, Visser M, Peila R, Nevitt MC, Cauley JA, Tylavsky FA, et al. Is a fall just a fall: Correlates of falling in healthy older persons. The health, aging and body composition study. Journal of the American Geriatrics Society. 2003;51(6):841-6.

391. Tromp E. Risk profiles and preventive measures of falls in elderly persons. Tijdschrift voor Gerontologie en Geriatrie. 2002;33(1):21-5.

392. Peeker R, Edlund C, Wennberg AL, Fall M. The treatment of sphincter incontinence with periurethral silicone implants (Macroplastique). Scandinavian Journal of Urology and Nephrology. 2002;36(3):194-8.

393. Mason M, Tully S. Urinary incontinence in the older acute care population: effects of knowledge, attitudes and beliefs of nurses on continence management. Perspectives (Gerontological Nursing Association (Canada)). 2002;26(3):4-9.

394. Ippolito C, Spisani L, De Luca G, Romano A, Daniele C, Cartei F, et al. Role of transrectal ultrasound-sonography in prostatic brachytherapy. Archivio Italiano di Urologia e Andrologia. 2002;74(4):295-8.

395. Geisthövell F, Rabe T. Individualized therapeutic strategies of menopausal transition, and peri-/postmenopause - Update 2002. Reproduktionsmedizin. 2002;18(5):247-68.

396. Edlund C, Peeker R, Fall M. Urinary incontinence in men over 50. Journal of the British Menopause Society. 2002;8(2):63-8.

397. Tromp AM, Pluijm SMF, Smit JH, Deeg DJH, Bouter LM, Lips P. Fall-risk screening test: A prospective study on predictors for falls in community-dwelling elderly. Journal of Clinical Epidemiology. 2001;54(8):837-44.

398. Sze KH, Wong E, Leung HY, Woo J. Falls among Chinese stroke patients during rehabilitation. Archives of Physical Medicine and Rehabilitation. 2001;82(9):1219-25.

399. Edlund C, Hellström M, Peeker R, Fall M. First Scandinavian experience of electrical sacral nerve stimulation in the treatment of the overactive bladder. Scandinavian Journal of Urology and Nephrology. 2000;34(6):366-76.

400. Wilkins K. Medications and fall-related fractures in the elderly. Health reports / Statistics Canada, Canadian Centre for Health Information = Rapports sur la santé / Statistique Canada, Centre canadien d'information sur la santé. 1999;11(1):45-53.

401. Wehrend A, Funk J. Case report. Urinary incontinence in a newborn foal. Tierärztliche Praxis Ausgabe G, Grosstiere/Nutztiere. 1999;27(1):8, 52-3.

402. Sáez Aldana F, Martínez Galarreta MV, Martínez-Íñiguez Blasco J. Analysis of falls producing hip fracture in the elderly. Revista de Ortopedia y Traumatologia. 1999;43(2):99-106.

403. Gregory SP, Holt PE, Parkinson TJ, Wathes CM. Vaginal position and length in the bitch: relationship to spaying and urinary incontinence. The Journal of small animal practice. 1999;40(4):180-4.

404. Fall M. Urinary incontinence in men--a neglected problem? A quarter of all 80-year old men suffer of urinary leakage. Läkartidningen. 1999;96(18):2227-31.

405. Fall M. Advantages and pitfalls of functional electrical stimulation. Acta Obstetricia et Gynecologica Scandinavica, Supplement. 1998;77(168):16-21.

406. Tutuarima JA, Van Der Meulen JHP, De Haan RJ, Van Straten A, Limburg M. Risk factors for falls of hospitalized stroke patients. Stroke. 1997;28(2):297-301.

407. Nyberg L, Gustafson Y. Fall prediction index for patients in stroke rehabilitation. Stroke. 1997;28(4):716-21.

408. Méndez Rubio JI, Zunzunegui MV, Béland F. The prevalence of and factors associated with falls in older persons living in the community. Medicina clínica. 1997;108(4):128-32.

409. Engberg S, McDowell BJ, Weber E, Brodak I, Donovan N, Engberg R. Assessment and management of urinary incontinence among homebound older adults: a clinical trial protocol. Advanced practice nursing quarterly. 1997;3(2):48-56.

410. Brandeis GH, Baumann MM, Hossain M, Morris JN, Resnick NM. The prevalence of potentially remediable urinary incontinence in frail older people: A study using the minimum data set. Journal of the American Geriatrics Society. 1997;45(2):179-84.

411. Meier C, Hess C, Meier-Abt PJ, Krahenbuhl S. Effects of cisapride on the function of the urinary bladder. Schweizerische Rundschau fur Medizin/Praxis. 1996;85(3):62-4.

412. Hahn I, Milsom I, Ohlsson BL, Ekelund P, Uhlemann C, Fall M. Comparative assessment of pelvic floor function using vaginal cones, vaginal digital palpation and vaginal pressure measurements. Gynecologic and Obstetric Investigation. 1996;41(4):269-74.

413. Abate G, Zito M, Puddu GM, Di Iorio A. Physiopathologic considerations and therapeutic implications in arterial hypertension in the elderly. Giornale di Gerontologia. 1996;44(4):273-317.

414. Juma S. Anterior vaginal suspension for vaginal vault prolapse. Techniques in urology. 1995;1(3):150-6.

415. Achilli MP, Guarnaschelli C, Arrigoni N, Felicetti G, Zelaschi F, Scoppetta FP, et al. Uroflowmetric evaluation of stress and emergency urinary incontinence treated with perineal rehabilitation. Europa Medicophysica. 1995;31(1):45-8.

416. List J, Stendel R, Rudolph KH, Brock M. Manifestation of split cord malformation type I (diastematomyelia) in adults. Zentralblatt fur Neurochirurgie. 1994;55(4):212-7.

417. Fowler CJ, Beck RO, Gerrard S, Betts CD, Fowler CG. Intravesical capsaicin for treatment of detrusor hyperreflexia. Journal of Neurology Neurosurgery and Psychiatry. 1994;57(2):169-73.

418. Fall M, Lindstrom S. Functional electrical stimulation: Physiological basis and clinical principles. International Urogynecology Journal. 1994;5(5):296-304.

419. Schnizer W. Therapeutic muscle training. Wiener klinische Wochenschrift. 1993;105(8):232-8.

420. Hahn I, Milson I, Fall M, Ekelund P. Long-term results of pelvic floor training in female stress urinary incontinence. British Journal of Urology. 1993;72(4):421-7.

421. Geirsson G, Fall M, Lindstrom S. Subtypes of overactive bladder in old age. Age and Ageing. 1993;22(2):125-31.

422. Boccafoschi C, Annoscia S, Lozzi C, Signorello D. Vesico-urethral and entero-urethral anastomosis: anatomo-surgical considerations and technical note. Archivio italiano di urologia, andrologia : organo ufficiale [di] Società italiana di ecografia urologica e nefrologica / Associazione ricerche in urologia. 1993;65(5):563-9.

423. Milsom I, Fall M, Ekelund P. Urinary incontinence--an expensive national disease. Läkartidningen. 1992;89(20):1772-4.

424. Simeonova Z, Bengtsson C, Ekelund P, Milsom I. Urinary incontinence and other urogenital problems in women--cases for primary health care? Läkartidningen. 1991;88(50):4329-31.

425. Hahn I, Sommar S, Fall M. A comparative study of pelvic floor training and electrical stimulation for the treatment of genuine female stress urinary incontinence. Neurourology and Urodynamics. 1991;10(6):545-54.

426. Hahn I, Fall M. Objective quantification of stress urinary incontinence: A short, reproducible, provocative pad-test. Neurourology and Urodynamics. 1991;10(5):475-81.

427. Fall M, Lindstrom S. Electrical stimulation: A physiologic approach to the treatment of urinary incontinence. Urologic Clinics of North America. 1991;18(2):393-407.

428. DuBeau CE, Resnick NM. Evaluation of the causes and severity of geriatric incontinence: A critical appraisal. Urologic Clinics of North America. 1991;18(2):243-56.

429. Kohorn EI. The surgery of stress urinary incontinence. Obstetrics and Gynecology Clinics of North America. 1989;16(4):841-52.

430. Mazieres L, Robain G, Perrigot M. Neurophysiological basis for the therapeutic effects of perineal stimulation in urinary incontinence. Annales de Readaptation et de Medecine Physique. 1987;30(4):463-7.

431. Fall M, Frankenberg S, Frisen M. 456,000 Swedes may have urinary incontinence. Only 25% seek medical help. Lakartidningen. 1985;82(22):2054-6.

432. Spangler PF, Risley TR, Bilyew DD. The management of dehydration and incontinence in nonambulatory geriatric patients. Journal of applied behavior analysis. 1984;17(3):397-401.

433. Fall M, Erlandson BE, Pettersson S. Evaluation of history and simple supine cystometry as a preoperative test in stress urinary incontinence. Acta Obstetricia et Gynecologica Scandinavica. 1984;63(3):241-4.

434. Fall M. Does electrostimulation cure urinary incontinence. Journal of Urology. 1984;131(4):664-7.

435. Carlsson CA, Erlandson BE, Fall M. Electrostimulation in disorders of the voiding mechanism of the bladder. Lakartidningen. 1979;76(12):1085-7.

436. Kishida S, Uchida E, Kakei K, Takamura N. Exophthalmos, a left cervical mass, somnolence, fecal and urinary incontinence (biopsy of the mass): (reticulum cell sarcoma). Nippon rinsho Japanese journal of clinical medicine. 1977;35 Suppl 2:3174-5, 540-541.

437. Erlandson BE, Fall M. Intravaginal electrical stimulation in urinary incontinence: An experimental and clinical study. Scandinavian Journal of Urology and Nephrology. 1977;SUPP. 44.

438. Melchior H. Incontinence of urine; diagnosis and treatment. Therapiewoche. 1976;26(37):5645-53.

439. Hodgkinson CP. Stress urinary incontinence in the human female. Gravity and ecomorphologic influences on bladder and urethral function of the human female. Henry Ford Hospital medical journal. 1969;17(3):145-64.

**Web of Science: 702studies**

1. Zullo AR, Sorial MN, Lee Y, Lary CW, Kiel DP, Berry SD. Predictors of Hip Fracture Despite Treatment with Bisphosphonates among Frail Older Adults. Journal of the American Geriatrics Society. 2020;68(2):256-60.

2. Ye PP, Liu YS, Zhang J, Peng K, Pan XR, Shen Y, et al. Falls prevention interventions for community-dwelling older people living in mainland China: a narrative systematic review. Bmc Health Services Research. 2020;20(1).

3. Spaltenstein J, Bula C, Santos-Eggimann B, Krief H, Seematter-Bagnoud L. Factors associated with going outdoors frequently: a cross-sectional study among Swiss community-dwelling older adults. Bmj Open. 2020;10(8).

4. Shin J, Han SH, Choi J, Kim YS, Lee J. Importance of Geriatric Syndrome Screening within 48 Hours of Hospitalization for Identifying Readmission Risk: A Retrospective Study in an Acute-Care Hospital. Annals of Geriatric Medicine and Research. 2020;24(2):83-90.

5. Serati M, Tarcan T, Finazzi-Agro E, Soligo M, Braga A, Athanasiou S, et al. The bladder is an unreliable witness: The case for urodynamic investigations in female stress urinary incontinence. European Journal of Obstetrics & Gynecology and Reproductive Biology. 2020;244:35-7.

6. Schnitzer S, Bluher S, Teti A, Schaeffner E, Ebert N, Martus P, et al. Risk Profiles for Care Dependency: Cross-Sectional Findings of a Population-Based Cohort Study in Germany. Journal of Aging and Health. 2020;32(5-6):352-60.

7. Schluter PJ, Askew DA, Jamieson HA, Arnold EP. Urinary and fecal incontinence are independently associated with falls risk among older women and men with complex needs: A national population study. Neurourology and Urodynamics. 2020;39(3):945-53.

8. Savas S, Saka B, Akin S, Tasci I, Tasar PT, Tufan A, et al. The prevalence and risk factors for urinary incontinence among inpatients, a multicenter study from Turkey. Archives of Gerontology and Geriatrics. 2020;90.

9. Park J, Lee K, Lee K. Association between urinary urgency and falls among rural dwelling older women. Journal of Advanced Nursing. 2020;76(3):846-55.

10. Paquin MH, Duclos C, Lapierre N, Dubreucq L, Morin M, Meunier J, et al. The effects of a strong desire to void on gait for incontinent and continent older community-dwelling women at risk of falls. Neurourology and Urodynamics. 2020;39(2):642-9.

11. Oliveira CED, Felipe SGB, da Silva C, de Carvalho DB, Silva F, Figueiredo MDF, et al. Clinical and functional vulnerability of elderly people from a day center. Acta Paulista De Enfermagem. 2020;33.

12. Ni BY, Pu LH, Tan M, Wang Y, Hu XY, Liu ZY. THE FREQUENCY AND INFLUENCING FACTORS OF COMMON GERIATRIC SYNDROMES IN HOSPITALIZED PATIENTS. Acta Medica Mediterranea. 2020;36(3):2077-84.

13. Naidoo K, Waggie F, van Wyk JM. A review of geriatric care training in the undergraduate nursing and medical curricula at the University of KwaZulu-Natal, South Africa. African Journal of Health Professions Education. 2020;12(3):130-3.

14. Moon S, Chung HS, Yu JM, Na HR, Kim SJ, Ko KJ, et al. Impact of urinary incontinence on falls in the older population: 2017 national survey of older Koreans. Archives of Gerontology and Geriatrics. 2020;90.

15. Meyer I, Morgan SL, Markland AD, Szychowski JM, Richter HE. Pelvic floor disorder symptoms and bone strength in postmenopausal women. International Urogynecology Journal. 2020;31(9):1777-84.

16. McClurg D, Panicker J, Walker RW, Cunnington A, Deane KHO, Harari D, et al. Stimulation of the tibial nerve: a protocol for a multicentred randomised controlled trial for urinary problems associated with Parkinson's disease-STARTUP. Bmj Open. 2020;10(2).

17. Magnuszewski L, Swietek M, Kasiukiewicz A, Kuprjanowicz B, Baczek J, Wojszel ZB. Health, Functional and Nutritional Determinants of Falls Experienced in the Previous Year-A Cross-Sectional Study in a Geriatric Ward. International Journal of Environmental Research and Public Health. 2020;17(13).

18. Lee K, Davis MA, Marcotte JE, Pressler SJ, Liang J, Gallagher NA, et al. Falls in community-dwelling older adults with heart failure: A retrospective cohort study. Heart & Lung. 2020;49(3):238-50.

19. Kachru N, Holmes HM, Johnson ML, Chen H, Aparasu RR. Risk of Mortality Associated with Non-selective Antimuscarinic medications in Older Adults with Dementia: a Retrospective Study. Journal of General Internal Medicine. 2020;35(7):2084-93.

20. Isbell T, Bordes JD, Murdock C, Siddiqui G, Rianon N. Diuretic use is not associated with falls in older women with urinary incontinence: preliminary data. Journal of the American Geriatrics Society. 2020;68:S289-S.

21. High R, Thai K, Virani H, Kuehl T, Danford J. Prevalence of Pelvic Floor Disorders in Female CrossFit Athletes. Female Pelvic Medicine and Reconstructive Surgery. 2020;26(8):498-502.

22. Hentzen C, Villaume A, Turmel N, Miget G, Le Breton F, Chesnel C, et al. Time to be Ready to Void: A new tool to assess the time needed to perform micturition for patients with multiple sclerosis. Annals of Physical and Rehabilitation Medicine. 2020;63(2):99-105.

23. Fitz F, Duclos C, Paquin M, Lapierre N, Rousseau J, Dumoulin C. IMPACT OF AN URGENT DESIRE TO VOID ON TRUNK AND PELVIS MOVEMENT IN OLDER WOMEN WITH URINARY INCONTINENCE WHO HAVE EXPERIENCED FALLS. Neurourology and Urodynamics. 2020;39:S302-S4.

24. Ebot J, Domingo R, Garcia HR, Chen S. Intradural Thoracic Arachnoid Cyst Fenestration for Spinal Cord Compression: A Case Illustration and Video Demonstration. Cureus. 2020;12(1).

25. Dumoulin C, Le Berre M. Pelvic Floor Muscle Training for Older Women with Urinary Incontinence. Current Geriatrics Reports. 2020;9(2):54-63.

26. Dokuzlar O, Okudur SK, Soysal P, Kocyigit SE, Yavuz I, Smit L, et al. Factors that Increase Risk of Falling in Older Men according to Four Different Clinical Methods. Experimental Aging Research. 2020;46(1):83-92.

27. Dokuzlar O, Okudur SK, Smith L, Soysal P, Yavuz I, Aydin AE, et al. Assessment of factors that increase risk of falling in older women by four different clinical methods. Aging Clinical and Experimental Research. 2020;32(3):483-90.

28. Cho ST, Moon S, Kim SJ, Ko KJ, Choi DK, Kwon O, et al. THE ASSOCIATION BETWEEN URINARY INCONTINENCE AND FALLS: A SYSTEMATIC REVIEW AND META-ANALYSIS. Journal of Urology. 2020;203:E154-E.

29. Cheng YC, Su CH. Evidence Supports PA Prescription for Parkinson's Disease: Motor Symptoms and Non-Motor Features: A Scoping Review. International Journal of Environmental Research and Public Health. 2020;17(8).

30. Chen PL, Lin HY, Ong JR, Ma HP. Development of a fall-risk assessment profile for community-dwelling older adults by using the National Health Interview Survey in Taiwan. Bmc Public Health. 2020;20(1).

31. Britting S, Artzi-Medvedik R, Fabbietti P, Tap L, Mattace-Raso F, Corsonello A, et al. Kidney function and other factors and their association with falls updates The screening for CKD among older people across Europe (SCOPE) study. Bmc Geriatrics. 2020;20.

32. Boockvar KS, Song W, Lee S, Intrator O. Comparing Outcomes Between Thiazide Diuretics and Other First-line Antihypertensive Drugs in Long-term Nursing Home Residents. Clinical Therapeutics. 2020;42(4):583-91.

33. Aranyavalai T, Jalayondeja C, Jalayondeja W, Pichaiyongwongdee S, Kaewkungwal J, Laskin JJ. Association between walking 5000 step/day and fall incidence over six months in urban community-dwelling older people. Bmc Geriatrics. 2020;20(1).

34. Adamuz J, Juve-Udina ME, Gonzalez-Samartino M, Jimenez-Martinez E, Tapia-Perez M, Lopez-Jimenez MM, et al. Care complexity individual factors associated with adverse events and in-hospital mortality. Plos One. 2020;15(7).

35. Abbs E, Brown R, Guzman D, Kaplan L, Kushel M. Risk Factors for Falls in Older Adults Experiencing Homelessness: Results from the HOPE HOME Cohort Study. Journal of General Internal Medicine. 2020;35(6):1813-20.

36. Re: Is There an Association between Urinary Incontinence and Mortality? A Retrospective Cohort Study Reply. Journal of Urology. 2020;204(2):354-6.

37. Yunusa I, Alsumali A, Garba AE, Regestein QR, Eguale T. Assessment of Reported Comparative Effectiveness and Safety of Atypical Antipsychotics in the Treatment of Behavioral and Psychological Symptoms of Dementia A Network Meta-analysis. Jama Network Open. 2019;2(3).

38. Yang YC, Ling MH, Wang CS, Lu FH, Wu JS, Cheng HP, et al. Geriatric syndromes and quality of life in older adults with diabetes. Geriatrics & Gerontology International. 2019;19(6):518-24.

39. Winoker JS, Say RK, Mehrazin R, Stock RG, Stone NN. Permanent prostate brachytherapy is safe in men with severe baseline lower urinary tract symptoms. Brachytherapy. 2019;18(3):332-7.

40. Wiedemann A, Kirschner-Hermanns R, Heppner HJ. Palliative long-term urinary bladder drainage: the uro-geriatric point of view. Urologe. 2019;58(4):389-97.

41. Walker NAF, Syed O, Malde S, Taylor C, Sahai A. Onabotulinum toxin A Injections in Men With Refractory Idiopathic Detrusor Overactivity. Urology. 2019;123:242-6.

42. Vejux J, Ben-Sadoun G, Piolet D, Bernat V, Ould-Aoudia V, Berrut G. Screening risk and protective factors of nursing home admission. Geriatrie Et Psychologie Neuropsychiatrie De Vieillissement. 2019;17(1):39-50.

43. Vaughan CP, Burgio KL, Goode PS, Juncos JL, McGwin G, Muirhead L, et al. Behavioral therapy for urinary symptoms in Parkinson's disease: A randomized clinical trial. Neurourology and Urodynamics. 2019;38(6):1737-44.

44. Thapa S, Shmerling RH, Bean JF, Cai YR, Leveille SG. Chronic multisite pain: evaluation of a new geriatric syndrome. Aging Clinical and Experimental Research. 2019;31(8):1129-37.

45. Taylor JL, Roberts L, Hladek MD, Liu MH, Nkimbeng M, Boyd CM, et al. Achieving self-management goals among low income older adults with functional limitations. Geriatric Nursing. 2019;40(4):424-30.

46. Tannenbaum C, Fritel X, Halme A, van den Heuvel E, Jutai J, Wagg A. Long-term effect of community-based continence promotion on urinary symptoms, falls and healthy active life expectancy among older women: cluster randomised trial. Age and Ageing. 2019;48(4):526-32.

47. Takeuchi T, Yajima K. Long-term 4 Years Follow-up Study of 482 Patients Who Underwent Shunting for Idiopathic Normal Pressure Hydrocephalus -Course of Symptoms and Shunt Efficacy Rates Compared by Age Group. Neurologia Medico-Chirurgica. 2019;59(7):281-6.

48. Szabo SM, Gooch K, Schermer C, Walker D, Lozano-Ortega G, Rogula B, et al. Association between cumulative anticholinergic burden and falls and fractures in patients with overactive bladder: US-based retrospective cohort study. Bmj Open. 2019;9(5).

49. Smith K, Sutherland A, Hyde Z, Crawford R, Dwyer A, Malay R, et al. Assessment, incidence and factors associated with urinary incontinence in older Aboriginal Australians. Internal Medicine Journal. 2019;49(9):1111-8.

50. Singh DKA, Shahar S, Vanoh D, Kamaruzzaman SB, Tan MP. Diabetes, arthritis, urinary incontinence, poor self-rated health, higher body mass index and lower handgrip strength are associated with falls among community-dwelling middle-aged and older adults: Pooled analyses from two cross-sectional Malaysian datasets. Geriatrics & Gerontology International. 2019;19(8):798-803.

51. Shin JH. Nursing Staff Characteristics on Resident Outcomes in Nursing Homes. Journal of Nursing Research. 2019;27(1).

52. Shaw JS. Old wine into new wineskins: an update for female stress urinary incontinence. Current Opinion in Obstetrics & Gynecology. 2019;31(6):494-500.

53. Scelzo E, Beghi E, Rosa M, Angrisano S, Antonini A, Bagella C, et al. Deep brain stimulation in Parkinson's disease: A multicentric, long-term, observational pilot study. Journal of the Neurological Sciences. 2019;405.

54. Sayabalian A, Easton-Garrett S, Kassabian A, Kunze MB. Incontinence affects every aspect and stakeholder of an assisted living community. Geriatric Nursing. 2019;40(3):338-41.

55. Rausch C, Liang Y, Bultmann U, de Rooij SE, Johnell K, Laflamme L, et al. Social position and geriatric syndromes among Swedish older people: a population-based study. Bmc Geriatrics. 2019;19(1).

56. Pereira DT, Schwab ML, Ferrarin DA, Ripplinger A, Aiello G, Herculano LF, et al. Vertebral Fractures and Luxation in Dogs. Acta Scientiae Veterinariae. 2019;47.

57. Peeters G, Cooper R, Tooth L, van Schoor NM, Kenny RA. A comprehensive assessment of risk factors for falls in middle-aged adults: co-ordinated analyses of cohort studies in four countries. Osteoporosis International. 2019;30(10):2099-117.

58. Oshiro CES, Frankland TB, Rosales AG, Perrin NA, Bell CL, Lo SHY, et al. Fall Ascertainment and Development of a Risk Prediction Model Using Electronic Medical Records. Journal of the American Geriatrics Society. 2019;67(7):1417-22.

59. Najafpour Z, Godarzi Z, Arab M, Yaseri M. Risk Factors for Falls in Hospital In-Patients: A Prospective Nested Case Control Study. International Journal of Health Policy and Management. 2019;8(5):300-6.

60. Moon S, Roh YK, Yoon JL, Jang KU, Jung HJ, Yoo HJ, et al. Clinical Features of Geriatric Syndromes in Older Koreans with Diabetes Mellitus. Annals of Geriatric Medicine and Research. 2019;23(4):176-82.

61. Min L, Tinetti M, Langa KM, Ha J, Alexander N, Hoffman G. Measurement of Fall Injury With Health Care System Data and Assessment of Inclusiveness and Validity of Measurement Models. Jama Network Open. 2019;2(8).

62. Miki Y, Foti SC, Asi YT, Tsushima E, Quinn N, Ling H, et al. Improving diagnostic accuracy of multiple system atrophy: a clinicopathological study. Brain. 2019;142:2813-27.

63. Mallol D, Taveras R, Hartman J, Granville M, Jacobson RE. Cauda Equina Syndrome in a Patient with Intradural Schwannoma at the Same Level as an Acute L2 Compression Fracture. Cureus. 2019;11(8).

64. Longo M, Bellastella G, Maiorino MI, Meier JJ, Esposito K, Giugliano D. Diabetes and Aging: From Treatment Goals to Pharmacologic Therapy. Frontiers in Endocrinology. 2019;10.

65. Lipowski M, Kucharska-Lipowska M, Brola W. Urological complications after stroke. Aktualnosci Neurologiczne. 2019;19(3):125-31.

66. Lino VTS, Rodrigues NCP, Andrade MKD, Reis IND, Lopes LAE, Atie S. Association between visual problems, insufficient emotional support and urinary incontinence with disability in elderly people living in a poor district in Rio de Janeiro, Brazil: A six-year follow-up study. Plos One. 2019;14(5).

67. Le Berre M, Morin M, Corriveau H, Hamel M, Nadeau S, Filiatrault J, et al. Characteristics of Lower Limb Muscle Strength, Balance, Mobility, and Function in Older Women with Urge and Mixed Urinary Incontinence: An Observational Pilot Study. Physiotherapy Canada. 2019;71(3):250-60.

68. Korall AMB, Feldman F, Yang YJ, Cameron ID, Leung PM, Sims-Gould J, et al. Effectiveness of Hip Protectors to Reduce Risk for Hip Fracture from Falls in Long-Term Care. Journal of the American Medical Directors Association. 2019;20(11):1397-+.

69. Jerez-Roig J, Moreira FSM, da Camara SMA, Ferreira L, Lima KC. Predicting continence decline in institutionalized older people: A longitudinal analysis. Neurourology and Urodynamics. 2019;38(3):958-67.

70. Iliescu C, Iliescu D, Miron I, Halitchi LG. IS UROFLOWMETRIA A USEFUL EXPLORATION IN CHILD'S MICTIONAL DISORDERS? International Journal of Medical Dentistry. 2019;23(1):24-31.

71. Huang MH, Blackwood J, Godoshian M, Pfalzer L. Predictors of falls in older survivors of breast and prostate cancer: A retrospective cohort study of surveillance, epidemiology and end results-Medicare health outcomes survey linkage. Journal of Geriatric Oncology. 2019;10(1):89-97.

72. Griebling TL. Re: Falls and Hip Fractures Associated with Urinary Incontinence among Older Men and Women with Complex Needs: A National Population Study. Journal of Urology. 2019;202(1):9-10.

73. Goba GK, Legesse AY, Zelelow YB, Gebreselassie MA, Rogers RG, Kenton KS, et al. Reliability and validity of the Tigrigna version of the Pelvic Floor Distress Inventory-Short Form 20 (PFDI-20) and Pelvic Floor Impact Questionnaire-7 (PFIQ-7). International Urogynecology Journal. 2019;30(1):65-70.

74. Giraldo-Rodrguez L, Agudelo-Botero M, Mino-Len D, Alvarez-Cisneros T. Epidemiology, progression, and predictive factors of urinary incontinence in older community-dwelling Mexican adults: Longitudinal data from the Mexican Health and Aging Study. Neurourology and Urodynamics. 2019;38(7):1932-43.

75. Fritsch MA, Shelton PS. Geriatric Polypharmacy Pharmacist as Key Facilitator in Assessing for Falls Risk: 2019 Update. Clinics in Geriatric Medicine. 2019;35(2):185-+.

76. Escobar-Aguilar G, Moreno-Casbas MT, Gonzalez-Maria E, Martinez-Gimeno ML, Sanchez-Pablo C, Orts-Cortes I. The SUMAMOS EXCELENCIA ProjectSUMAMOS EXCELENCIA <SIC><SIC>. Journal of Advanced Nursing. 2019;75(7):1575-84.

77. Eglseer D, Hodl M, Lohrmann C. Six Nursing Care Problems in Hospitals A Cross-Sectional Study of Quality of Care. Journal of Nursing Care Quality. 2019;34(1):E8-E14.

78. Duong E, Al Hamarneh YN, Tsuyuki RT, Wagg A, Hunter KF, Schulz J, et al. Case finding for urinary incontinence and falls in older adults at community pharmacies. Canadian Pharmacists Journal. 2019;152(4):228-33.

79. Creevy KE, Grady J, Little SE, Moore GE, Strickler BG, Thompson S, et al. 2019 AAHA Canine Life Stage Guidelines. Journal of the American Animal Hospital Association. 2019;55(6):267-90.

80. Coon EA, Rocca W, Melson CS, Ahlskog JE, Matsumoto JY, Low PA, et al. Conjugal multiple system atrophy: Chance, shared risk factors, or evidence of transmissibility? Parkinsonism & Related Disorders. 2019;67:10-3.

81. Cleutjens F, Boonen A, van Onna MGB. Geriatric syndromes in patients with rheumatoid arthritis: a literature overview. Clinical and Experimental Rheumatology. 2019;37(3):496-501.

82. Chu CM, Schmitz KH, Khanijow K, Stambakio H, Newman DK, Arya LA, et al. Feasibility and outcomes: Pilot Randomized Controlled Trial of a home-based integrated physical exercise and bladder-training program vs usual care for community-dwelling older women with urinary incontinence. Neurourology and Urodynamics. 2019;38(5):1399-408.

83. Chiu CJ, Cheng YY. Utility of Geriatric Syndrome Indicators for Predicting Subsequent Health Care Utilization in Older Adults in Taiwan. International Journal of Environmental Research and Public Health. 2019;16(3).

84. Chen YJ, Wilson L, Kornak J, Dudley RA, Merrilees J, Bonasera SJ, et al. The costs of dementia subtypes to California Medicare fee-for-service, 2015. Alzheimers & Dementia. 2019;15(7):899-906.

85. Caramaschi IK, Botelho TL, Silva LO, Gimenez MM, Ferreira LA, Bortolini M, et al. CORRELATION BETWEEN BALANCE AND FALLS AND DEGREE OF BOTHER WITH PELVIC FLOOR SYMPTOMS IN WOMEN WITH URINARY INCONTINENCE. International Urogynecology Journal. 2019;30:S325-S.

86. Caplan EO, Abbass IM, Suehs BT, Ng DB, Gooch K, van Amerongen D. Impact of Coexisting Overactive Bladder in Medicare Patients With Dementia on Clinical and Economic Outcomes. American Journal of Alzheimers Disease and Other Dementias. 2019;34(7-8):492-9.

87. Bytyci A, Ymerhalili G, Dorner TE, Stein KV, Maxhera O. Pilot study of an integrated care model using the geriatric assessment tool. Wiener Klinische Wochenschrift. 2019;131(11-12):273-7.

88. Biryukova EV. TYPE 2 DIABETES MELLITUS IN ELDERLY PATIENTS TREATED WITH ENDOCRINOLOGISTS IN CLINICAL PRACTICE. Diabetes Mellitus. 2019;22(6):582-91.

89. Austin AM, Carmichael D, Berry S, Gozansky WS, Nelson EC, Skinner JS, et al. Chronic Condition Measurement Requires Engagement, Not Measurement Alone. Journal of Ambulatory Care Management. 2019;42(4):295-304.

90. Almajnooni RS, Sulaimani AA, Bakhsh AM, Shoushou IM, Almuqaytif AM, Alreshaid KM, et al. GERIATRIC SYNDROMES: SYSTEMATIC LITERATURE REVIEW. Indo American Journal of Pharmaceutical Sciences. 2019;6(1):943-7.

91. Aldaz T, Nigro P, Sanchez-Gomez A, Painous C, Planellas L, Santacruz P, et al. Non-motor symptoms in Huntington's disease: a comparative study with Parkinson's disease. Journal of Neurology. 2019;266(6):1340-50.

92. Yuaso DR, Santos JLF, Castro RA, Duarte YAO, Girao M, Berghmans B, et al. Female double incontinence: prevalence, incidence, and risk factors from the SABE (Health, Wellbeing and Aging) study. International Urogynecology Journal. 2018;29(2):265-72.

93. Valencia WM, Botros D, Vera-Nunez M, Dang S. Diabetes Treatment in the Elderly: Incorporating Geriatrics, Technology, and Functional Medicine. Current Diabetes Reports. 2018;18(10).

94. Valderrama-Hinds LM, Al Snih S, Chen NW, Rodriguez MA, Wong R. Falls in Mexican older adults aged 60years and older. Aging Clinical and Experimental Research. 2018;30(11):1345-51.

95. Tkacheva ON, Runikhina NK, Ostapenko VS, Sharashkina NV, Mkhitaryan EA, Onuchina JS, et al. Prevalence of geriatric syndromes among people aged 65 years and older at four community clinics in Moscow. Clinical Interventions in Aging. 2018;13:251-9.

96. Taylor DA, Merten SL, Sandercoe GD, Gahankari D, Ingram SB, Moncrieff NJ, et al. Abdominoplasty Improves Low Back Pain and Urinary Incontinence. Plastic and Reconstructive Surgery. 2018;141(3):637-45.

97. Tatum PE, Talebreza S, Ross JS. Geriatric Assessment: An Office-Based Approach. American Family Physician. 2018;97(12):776-84.

98. Talarska D, Tobis S, Kotkowiak M, Strugala M, Stanislawska J, Wieczorowska-Tobis K. Determinants of Quality of Life and the Need for Support for the Elderly with Good Physical and Mental Functioning. Medical Science Monitor. 2018;24:1604-13.

99. Sohn K, Lee CK, Shin J, Lee J. Association between Female Urinary Incontinence and Geriatric Health Problems: Results from Korean Longitudinal Study of Ageing (2006). Korean Journal of Family Medicine. 2018;39(1):10-4.

100. Smith EM, Shah AA. Screening for Geriatric Syndromes: Falls, Urinary/Fecal Incontinence, and Osteoporosis. Clinics in Geriatric Medicine. 2018;34(1):55-+.

101. Schluter PJ, Arnold EP, Jamieson HA. Falls and hip fractures associated with urinary incontinence among older men and women with complex needs: A national population study. Neurourology and Urodynamics. 2018;37(4):1336-43.

102. Ronneikko JK, Jamsen ER, Makela M, Finne-Soveri H, Valvanne JN. Reasons for home care clients' unplanned Hospital admissions and their associations with patient characteristics. Archives of Gerontology and Geriatrics. 2018;78:114-26.

103. Powell LC, Szabo SM, Walker D, Gooch K. The economic burden of overactive bladder in the United States: A systematic literature review. Neurourology and Urodynamics. 2018;37(4):1241-9.

104. Onder G, Giovannini S, Sganga F, Manes-Gravina E, Topinkova E, Finne-Soveri H, et al. Interactions between drugs and geriatric syndromes in nursing home and home care: results from Shelter and IBenC projects. Aging Clinical and Experimental Research. 2018;30(9):1015-21.

105. Omura Y. Frequent waking up for urination &/or painful leg cramp(s) in advanced aged patients during sleep was one of important causes of fall & fracture which are major causes of disability & death. These problems can be reduced by simple change of available pillow from regular position to vertical position which can cover back of both head & part of chest which improve abnormal condition of spine & Thymus gland function. Acupuncture & Electro-Therapeutics Research. 2018;43(1):1-18.

106. Nieto-Riveiro L, Groba B, Miranda MC, Concheiro P, Pazos A, Pousada T, et al. Technologies for participatory medicine and health promotion in the elderly population. Medicine. 2018;97(20).

107. Mukhtar S, Imran R, Zaheer M, Tariq H. Frequency of non-motor symptoms in Parkinson's disease presenting to tertiary care centre in Pakistan: an observational, cross-sectional study. Bmj Open. 2018;8(5).

108. Molina-Garrido MJ, Guillen-Ponce C, Blanco R, Saldana J, Feliu J, Antonio M, et al. Delphi consensus of an expert committee in oncogeriatrics regarding comprehensive geriatric assessment in seniors with cancer in Spain. Journal of Geriatric Oncology. 2018;9(4):337-45.

109. Lo TS, Uy-Patrimonio MC, Hsieh WC, Yang JC, Huang SY, Chua S. Sacrospinous ligament fixation for hysteropexy: does concomitant anterior and posterior fixation improve surgical outcome? International Urogynecology Journal. 2018;29(6):811-9.

110. Liang YJ, Rausch C, Laflamme L, Moller J. Prevalence, trend and contributing factors of geriatric syndromes among older Swedes: results from the Stockholm County Council Public Health Surveys. Bmc Geriatrics. 2018;18.

111. Lewandowicz A, Skowronek P, Maksymiuk-Klos A, Piatkiewicz P. The Giant Geriatric Syndromes Are Intensified by Diabetic Complications. Gerontology and Geriatric Medicine. 2018;4.

112. Kuntz S, Dassen T, Lahmann NA. Specific item patterns in comparison to generalized sum score-the Care Dependency Scale (CDS) as a screening tool for specific care problems. Journal of Evaluation in Clinical Practice. 2018;24(4):731-9.

113. Kim S, Park JH, Won CW. Combined effects of four major geriatric syndromes on adverse outcomes based on Korean National Health Insurance claims data. Geriatrics & Gerontology International. 2018;18(10):1463-8.

114. Kim KS, Nam JW, Choi BY, Moon HS. The association of lower urinary tract symptoms with incidental falls and fear of falling in later life: The Community Health Survey. Neurourology and Urodynamics. 2018;37(2):775-84.

115. Kemmler W, Weissenfels A, Willert S, Shojaa M, von Stengel S, Filipovic A, et al. Efficacy and Safety of Low Frequency Whole-Body Electromyostimulation (WB-EMS) to Improve Health-Related Outcomes in Non-athletic Adults. A Systematic Review. Frontiers in Physiology. 2018;9.

116. Karabulut A, Simavli S, Demirtas O, Ok N, Gungor HR, Zumrutbas A. Evaluation of overactive bladder and nocturia as a risk factor for hip fracture in climacteric women: a matched pair case control study. Journal of Obstetrics and Gynaecology. 2018;38(2):252-6.

117. Kao LT, Huang CY, Lin HC, Chu CM. No Increased Risk of Fracture in Patients Receiving Antimuscarinics for Overactive Bladder Syndrome: A Retrospective Cohort Study. Journal of Clinical Pharmacology. 2018;58(6):727-32.

118. Kang J, Kim C. Association between urinary incontinence and physical frailty in Korea. Australasian Journal on Ageing. 2018;37(3):E104-E9.

119. Kanevetci Z, Yaman H. Health Needs of Elderly People at Primary Health Care Centers in Antalya, Turkey. World Family Medicine. 2018;16(5):4-9.

120. Jorn HKS. URINARY INCONTINENCE AFTER A FALL WHILE ANTICOAGULATED: EFFECT OF A HEMATOMA IN AN UNLIKELY LOCATION. Journal of General Internal Medicine. 2018;33:S652-S3.

121. Jayadevappa R, Chhatre S, Newman DK, Schwartz JS, Wein AJ. Association between overactive bladder treatment and falls among older adults. Neurourology and Urodynamics. 2018;37(8):2688-94.

122. Hare N, Georgopoulos P, Philips KE, Johnson JE, Seary C, Panicker JN, et al. Improvement in overactive bladder symptoms in patients using functional electrical stimulation of the common peroneal nerve for walking. Clinical Rehabilitation. 2018;32(10):1357-62.

123. Gibson W, Hunter KF, Camicioli R, Booth J, Skelton DA, Dumoulin C, et al. The association between lower urinary tract symptoms and falls: Forming a theoretical model for a research agenda. Neurourology and Urodynamics. 2018;37(1):501-9.

124. Gale CR, Westbury LD, Cooper C, Dennison EM. Risk factors for incident falls in older men and women: the English longitudinal study of ageing. Bmc Geriatrics. 2018;18.

125. Fonseca ADG, Silva C, Barbosa DA, Alves ECS, de Pinho L, Brito M, et al. Factors associated to the dependence of older adults with diabetes mellitus type 2. Revista Brasileira De Enfermagem. 2018;71:868-75.

126. Feng H, Li H, Xiao LD, Ullah S, Mao P, Yang YX, et al. Aged care clinical mentoring model of change in nursing homes in China: study protocol for a cluster randomized controlled trial. Bmc Health Services Research. 2018;18.

127. Elghblawi E. Platelet-rich plasma, the ultimate secret for youthful skin elixir and hair growth triggering. Journal of Cosmetic Dermatology. 2018;17(3):423-30.

128. Ege S. Management of urinary incontinence in a geriatric rehabilitation department. Zeitschrift Fur Gerontologie Und Geriatrie. 2018;51(3):301-13.

129. DeFoor WR, Inge TH, Jenkins TM, Jackson E, Courcoulas A, Michalsky M, et al. Prospective evaluation of urinary incontinence in severely obese adolescents presenting for weight loss surgery. Surgery for Obesity and Related Diseases. 2018;14(2):214-8.

130. Chmielewska D, Sobota G, Stania M, Blaszczak E, Slomka K, Juras G. A comparison of a step-initiation task in women with and without urinary incontinence. A case-control study. Neurourology and Urodynamics. 2018;37(8):2571-7.

131. Caplan EO, Abbass IM, Suehs BT, Ng DB, Gooch K, Kirby C, et al. Impact of coexisting overactive bladder in Medicare patients with osteoporosis. Archives of Gerontology and Geriatrics. 2018;75:44-50.

132. Bulut EA, Soysal P, Isik AT. Frequency and coincidence of geriatric syndromes according to age groups: single-center experience in Turkey between 2013 and 2017. Clinical Interventions in Aging. 2018;13:1899-905.

133. Booth L, Skelton DA, Hagen S, Booth J. Age and gender stratified normative values for the International Prostate Symptom Score for adults aged 60 years and over. Neurourology and Urodynamics. 2018;37(8):2732-9.

134. Asan Z. Spinal Concussion in Adults: Transient Neuropraxia of Spinal Cord Exposed to Vertical Forces. World Neurosurgery. 2018;114:E1284-E9.

135. Agudelo-Botero M, Giraldo-Rodriguez L, Murillo-Gonzalez JC, Mino-Leon D, Cruz-Arenas E. Factors associated with occasional and recurrent falls in Mexican communityd-welling older people. Plos One. 2018;13(2).

136. Zheng FX, Lin ZD, Ye XY, Shi XL. Unusual brain images of a boy with adolescent cerebral X-linked adrenoleukodystrophy presenting with exhibitionism: A CARE-compliant case report. Medicine. 2017;96(51).

137. Yu WC, Chou MY, Peng LN, Lin YT, Liang CK, Chen LK. Synergistic effects of cognitive impairment on physical disability in all-cause mortality among men aged 80 years and over: Results from longitudinal older veterans study. Plos One. 2017;12(7).

138. Yakaryilmaz FD, Ozturk ZA. Treatment of type 2 diabetes mellitus in the elderly. World Journal of Diabetes. 2017;8(6):278-85.

139. Whittle AK, Kalsi T, Babic-Illman G, Wang Y, Fields P, Ross PJ, et al. A comprehensive geriatric assessment screening questionnaire (CGA-GOLD) for older people undergoing treatment for cancer. European Journal of Cancer Care. 2017;26(5).

140. Talarska D, Strugala M, Szewczyczak M, Tobis S, Michalak M, Wroblewska I, et al. Is independence of older adults safe considering the risk of falls? Bmc Geriatrics. 2017;17.

141. Ronneikko JK, Makela M, Jamsen ER, Huhtala H, Finne-Soveri H, Noro A, et al. Predictors for Unplanned Hospitalization of New Home Care Clients. Journal of the American Geriatrics Society. 2017;65(2):407-14.

142. Robson SJ, de Costa CM. Thirty years of the World Health Organization's target caesarean section rate: time to move on. Medical Journal of Australia. 2017;206(4):181-5.

143. Rashid N, Vassilakis M, Lin KJ, Kristy R, Ng DB. Primary Nonadherence to Overactive Bladder Medications in an Integrated Managed Care Health Care System. Journal of Managed Care & Specialty Pharmacy. 2017;23(4):484-93.

144. Ohtake PJ, Borello-France D. Rehabilitation for Women and Men With Pelvic-Floor Dysfunction. Physical Therapy. 2017;97(4):390-2.

145. Niznik J, Zhao XH, Jiang T, Hanlon J, Aspinall S, Thorpe J, et al. Anticholinergic Prescribing in Medicare Part D Beneficiaries Residing in Nursing Homes: Results from a Retrospective Cross-Sectional Analysis of Medicare Data. Drugs & Aging. 2017;34(12):925-39.

146. Mudge AM, Banks MD, Barnett AG, Blackberry I, Graves N, Green T, et al. CHERISH (collaboration for hospitalised elders reducing the impact of stays in hospital): protocol for a multi-site improvement program to reduce geriatric syndromes in older inpatients. Bmc Geriatrics. 2017;17.

147. Mooventhan A, Nivethitha L. Evidence based effects of yoga practice on various health related problems of elderly people: A review. Journal of Bodywork and Movement Therapies. 2017;21(4):1028-32.

148. Moga DC, Wu QS, Doshi P, Goodin AJ. An investigation of factors predicting the type of bladder antimuscarinics initiated in Medicare nursing homes residents. Bmc Geriatrics. 2017;17.

149. Mendes GAS, de Oliveira MF, Pinto FCG. The Timed Up and Go Test as a Diagnostic Criterion in Normal Pressure Hydrocephalus. World Neurosurgery. 2017;105:456-61.

150. Lutomski JE, Krabbe PFM, Bleijenberg N, Blom J, Kempen G, MacNeil-Vroomen J, et al. Measurement properties of the EQ-5D across four major geriatric conditions: Findings from TOPICS-MDS. Health and Quality of Life Outcomes. 2017;15.

151. Locatelli I, Monod S, Cornuz J, Bula CJ, Senn N. A prospective study assessing agreement and reliability of a geriatric evaluation. Bmc Geriatrics. 2017;17.

152. Liang CK, Chou MY, Chen LY, Wang KY, Lin SY, Chen LK, et al. Delaying cognitive and physical decline through multidomain interventions for residents with mild-to-moderate dementia in dementia care units in Taiwan: A prospective cohort study. Geriatrics & Gerontology International. 2017;17:36-43.

153. Kim S, Park JH, Ahn H, Lee S, Yoo HJ, Yoo J, et al. Risk Factors of Geriatric Syndromes in Korean Population. Annals of Geriatric Medicine and Research. 2017;21(3):123-30.

154. Jamieson HA, Schluter PJ, Pyun J, Arnold T, Scrase R, Nisbet-Abey R, et al. Fecal Incontinence Is Associated With Mortality Among Older Adults With Complex Needs: An Observational Cohort Study. American Journal of Gastroenterology. 2017;112(9):1431-7.

155. Inoue H, Kohata Y, Fukuda T, Monma M, Uzawa Y, Kubo Y, et al. Repair of damaged ligaments with tissue fixation system minisling is sufficient to cure major prolapse in all three compartments: 5-year data. Journal of Obstetrics and Gynaecology Research. 2017;43(10):1570-7.

156. Ibrahim A, Singh DKA, Shahar S. 'Timed Up and Go' test: Age, gender and cognitive impairment stratified normative values of older adults. Plos One. 2017;12(10).

157. Hung CH, Wang CJ, Tang TC, Chen LY, Peng LN, Hsiao FY, et al. Recurrent falls and its risk factors among older men living in the veterans retirement communities: A cross-sectional study. Archives of Gerontology and Geriatrics. 2017;70:214-8.

158. Huang TY, Liang CK, Shen HC, Chen HI, Liao MC, Chou MY, et al. Gait Speed rather than Dynapenia Is a Simple Indicator for Complex Care Needs: A Cross-sectional Study Using Minimum Data Set. Scientific Reports. 2017;7.

159. Huang CC, Lee JD, Yang DC, Shih HI, Sun CY, Chang CM. Associations Between Geriatric Syndromes and Mortality in Community-Dwelling Elderly: Results of a National Longitudinal Study in Taiwan. Journal of the American Medical Directors Association. 2017;18(3):246-51.

160. Hillen JB, Vitry A, Caughey GE. Disease burden, comorbidity and geriatric syndromes in the Australian aged care population. Australasian Journal on Ageing. 2017;36(2):E14-E9.

161. Held F, Le Couteur DG, Blyth FM, Hirani V, Naganathan V, Waite LM, et al. Polypharmacy in older adults: Association Rule and Frequent-Set Analysis to evaluate concomitant medication use. Pharmacological Research. 2017;116:39-44.

162. Hagen S, Glazener C, McClurg D, Macarthur C, Elders A, Herbison P, et al. Pelvic floor muscle training for secondary prevention of pelvic organ prolapse (PREVPROL): a multicentre randomised controlled trial. Lancet. 2017;389(10067):393-402.

163. Goeteyn J, Evans LA, De Cleyn S, Fauconnier S, Damen C, Hewitt J, et al. Frailty as a predictor of mortality in the elderly emergency general surgery patient. Acta Chirurgica Belgica. 2017;117(6):370-5.

164. Fritsch MA, Shelton PS. Geriatric Polypharmacy Pharmacist as Key Facilitator in Assessing for Falls Risk. Clinics in Geriatric Medicine. 2017;33(2):205-+.

165. Dufour S, Northwood M. URINARY INCONTINENCE AND FALLS: A DELPHI CONSENSUS. Neurourology and Urodynamics. 2017;36:S139-S40.

166. Dokuzlar O, Soysal P, Isik AT. Association between serum vitamin B12 level and frailty in older adults. Northern Clinics of Istanbul. 2017;4(1):22-8.

167. Day GS, Lim TS, Hassenstab J, Goate AM, Grant EA, Roe CM, et al. Differentiating cognitive impairment due to corticobasal degeneration and Alzheimer disease. Neurology. 2017;88(13):1273-81.

168. Damian J, Pastor-Barriuso R, Lopez FJG, de Pedro-Cuesta J. Urinary incontinence and mortality among older adults residing in care homes. Journal of Advanced Nursing. 2017;73(3):688-99.

169. Dahodwala N, Nwadiogbu C, Fitts W, Partridge H, Karlawish J. Parkinsonian signs are a risk factor for falls. Gait & Posture. 2017;55:1-5.

170. Chmielewska D, Stania M, Slomka K, Blaszczak E, Taradaj J, Dolibog P, et al. Static postural stability in women with stress urinary incontinence: Effects of vision and bladder filling. Neurourology and Urodynamics. 2017;36(8):2019-27.

171. Chang C, Callahan EH, Hung WW, Thomas DC, Leipzig RM, DeCherrie LV. A model for integrating the assessment and management of geriatric syndromes into internal medicine continuity practice: 5-year report. Gerontology & Geriatrics Education. 2017;38(3):271-82.

172. Carryer J, Weststrate J, Yeung P, Rodgers V, Towers A, Jones M. Prevalence of key care indicators of pressure injuries, incontinence, malnutrition, and falls among older adults living in nursing homes in New Zealand. Research in Nursing & Health. 2017;40(6):555-63.

173. Brown RT, Hemati K, Riley ED, Lee CT, Ponath C, Tieu L, et al. Geriatric Conditions in a Population-Based Sample of Older Homeless Adults. Gerontologist. 2017;57(4):757-66.

174. Bello-Chavolla OY, Aguilar-Salinas CA, Avila-Funes JA. GERIATRIC SYNDROMES AND NOT CARDIOVASCULAR RISK FACTORS ARE ASSOCIATED WITH COGNITIVE IMPAIRMENT AMONG MEXICAN COMMUNITY-DWELLING ELDERLY WITH TYPE 2 DIABETES. Revista De Investigacion Clinica-Clinical and Translational Investigation. 2017;69(3):166-72.

175. Barkin J, Habert J, Wong A, Lee LYT. The practical update for family physicians in the diagnosis and management of overactive bladder and lower urinary tract symptoms. Canadian Journal of Urology. 2017;24:1-11.

176. Balachandran A, Duckett J. Cystodistension: Is there evidence to support its use in current practice for patients with overactive bladder? Journal of Obstetrics and Gynaecology. 2017;37(6):700-3.

177. Anzaldi LJ, Davison A, Boyd CM, Leff B, Kharrazi H. Comparing clinician descriptions of frailty and geriatric syndromes using electronic health records: a retrospective cohort study. Bmc Geriatrics. 2017;17.

178. Wiedemann A. Falls and Urinary Incontinence: results of a systematic Literature Review. Zeitschrift Fur Gerontologie Und Geriatrie. 2016;49:S79-S80.

179. Vetrano DL, Foebel AD, Marengoni A, Brandi V, Collamati A, Heckman GA, et al. Chronic diseases and geriatric syndromes: The different weight of comorbidity. European Journal of Internal Medicine. 2016;27:62-7.

180. Silay K, Akinci S, Ulas A, Yalcin A, Silay YS, Akinci MB, et al. Occult urinary incontinence in elderly women and its association with geriatric condition. European Review for Medical and Pharmacological Sciences. 2016;20(3):447-51.

181. Schussler S, Dassen T, Lohrmann C. Care dependency and nursing care problems in nursing home residents with and without dementia: a cross-sectional study. Aging Clinical and Experimental Research. 2016;28(5):973-82.

182. Saraf AA, Petersen AW, Simmons SF, Schnelle JF, Bell SP, Kripalani S, et al. Medications Associated With Geriatric Syndromes and Their Prevalence in Older Hospitalized Adults Discharged to Skilled Nursing Facilities. Journal of Hospital Medicine. 2016;11(10):694-700.

183. Sakushima K, Yamazaki S, Fukuma S, Hayashino Y, Yabe I, Fukuhara S, et al. Influence of urinary urgency and other urinary disturbances on falls in Parkinson's disease. Journal of the Neurological Sciences. 2016;360:153-7.

184. Qin L, Luo XM, Zou KH, Snedecor SJ. Economic impact of using fesoterodine for the treatment of overactive bladder with urge urinary incontinence in a vulnerable elderly population in the United States. Journal of Medical Economics. 2016;19(3):229-35.

185. Pinkas J, Gujski M, Humeniuk E, Raczkiewicz D, Bejga P, Owoc A, et al. State of Health and Quality of Life of Women at Advanced Age. Medical Science Monitor. 2016;22:3095-105.

186. Phelan EA, Aerts S, Dowler D, Eckstrom E, Casey CM. Adoption of Evidence-Based Fall Prevention Practices in Primary Care for Older Adults with a History of Falls. Frontiers in Public Health. 2016;4.

187. Pahwa AK, Andy UU, Newman DK, Stambakio H, Schmitz KH, Arya LA. Noctural Enuresis as a Risk Factor for Falls in Older Community Dwelling Women with Urinary Incontinence. Journal of Urology. 2016;195(5):1512-6.

188. Ouslander JG, Naharci I, Engstrom G, Shutes J, Wolf DG, Rojido M, et al. Hospital Transfers of Skilled Nursing Facility (SNF) Patients Within 48 Hours and 30 Days After SNF Admission. Journal of the American Medical Directors Association. 2016;17(9):839-45.

189. Ouslander JG, Naharci I, Engstrom G, Shutes J, Wolf DG, Alpert G, et al. Lessons Learned From Root Cause Analyses of Transfers of Skilled Nursing Facility (SNF) Patients to Acute Hospitals: Transfers Rated as Preventable Versus Nonpreventable by SNF Staff. Journal of the American Medical Directors Association. 2016;17(7):596-601.

190. Otremba I. Risk factors for delirium - characteristics of patients at risk of delirium in Geriatric Ward. Family Medicine and Primary Care Review. 2016;18(1):39-43.

191. Noguchi N, Chan L, Cumming RG, Blyth FM, Naganathan V. A systematic review of the association between lower urinary tract symptoms and falls, injuries, and fractures in community-dwelling older men. Aging Male. 2016;19(3):168-74.

192. Noguchi N, Chan L, Cumming RG, Blyth FM, Handelsman DJ, Seibel MJ, et al. Lower Urinary Tract Symptoms and Incident Falls in Community Dwelling Older Men: The Concord Health and Ageing in Men Project. Journal of Urology. 2016;196(6):1694-9.

193. Noguchi N, Blyth FM, Waite LM, Naganathan V, Cumming RG, Handelsman DJ, et al. Prevalence of the geriatric syndromes and frailty in older men living in the community: The Concord Health and Ageing in Men Project. Australasian Journal on Ageing. 2016;35(4):255-61.

194. Neville CE. Bladder Control Problems in Elders: Assessment and Treatment Strategies for All Rehabilitation CliniciansClinical Commentary. Topics in Geriatric Rehabilitation. 2016;32(4):231-50.

195. Montera R, Plotti F, Ricciardi R, Miranda A, Venturella R, Zullo F, et al. Learning curves of a resident surgeon performing transobturator tape procedures for stress urinary incontinence. International Journal of Gynecology & Obstetrics. 2016;134(3):345-9.

196. Lukaszyk C, Harvey L, Sherrington C, Keay L, Tiedemann A, Coombes J, et al. Risk factors, incidence, consequences and prevention strategies for falls and fall-injury within older indigenous populations: a systematic review. Australian and New Zealand Journal of Public Health. 2016;40(6):564-8.

197. Lee JS, Kim SW, Fee SH, Kim JC, Choi JB, Cho SY, et al. Factors Affecting Quality of Life Among Spinal Cord Injury Patients in Korea. International Neurourology Journal. 2016;20(4):316-20.

198. Kent EE, Malinoff R, Rozjabek HM, Ambs A, Clauser SB, Topor MA, et al. Revisiting the Surveillance Epidemiology and End Results Cancer Registry and Medicare Health Outcomes Survey (SEER-MHOS) Linked Data Resource for Patient-Reported Outcomes Research in Older Adults with Cancer. Journal of the American Geriatrics Society. 2016;64(1):186-92.

199. Jung DH, Palta M, Smith M, Oliver TR, DuGoff EH. Differences in Receipt of Three Preventive Health Care Services by Race/Ethnicity in Medicare Advantage Plans: Tracking the Impact of Pay for Performance, 2010 and 2013. Preventing Chronic Disease. 2016;13.

200. Jonasson LL, Josefsson K. Staff experiences of the management of older adults with urinary incontinence. Healthy Aging Research. 2016;5.

201. Gibson W, Wagg A, Hunter KF. Urinary incontinence in older people. British Journal of Hospital Medicine. 2016;77(2):C27-C32.

202. Gibson W, Wagg A. Are older women more likely to receive surgical treatment for stress urinary incontinence since the introduction of the mid-urethral sling? An examination of Hospital Episode Statistics data. Bjog-an International Journal of Obstetrics and Gynaecology. 2016;123(8):1386-91.

203. Freemantle N, Khalaf K, Loveman C, Stanisic S, Gultyaev D, Lister J, et al. OnabotulinumtoxinA in the treatment of overactive bladder: a cost-effectiveness analysis versus best supportive care in England and Wales. European Journal of Health Economics. 2016;17(7):911-21.

204. Closs VE, Ziegelmann PK, Gomes I, Schwanke CHA. Frailty and geriatric syndromes in elderly assisted in primary health care. Acta Scientiarum-Health Sciences. 2016;38(1):9-18.

205. Chung A, Noguchi N, Chan L, Tse V. Voiding dysfunction in older men. Current Opinion in Urology. 2016;26(2):177-83.

206. Charandabi SM, Rezaei N, Hakimi S, Khatami S, Valizadeh R, Azadi A. Sleep disturbances and sexual function among men aged 45-75 years in an urban area of Iran. Sleep Science. 2016;9(1):29-34.

207. Brown J, King J. Age-stratified trends in 20 years of stress incontinence surgery in Australia. Australian & New Zealand Journal of Obstetrics & Gynaecology. 2016;56(2):192-8.

208. Boruah DK, Dhingani DD, Achar S, Prakash A, Augustine A, Sanyal S, et al. Magnetic Resonance Imaging Analysis of Caudal Regression Syndrome and Concomitant Anomalies in Pediatric Patients. Journal of Clinical Imaging Science. 2016;6.

209. Boronat-Garrido X, Kottner J, Schmitz G, Lahmann N. Incontinence-Associated Dermatitis in Nursing Homes Prevalence, Severity, and Risk Factors in Residents With Urinary and/or Fecal Incontinence. Journal of Wound Ostomy and Continence Nursing. 2016;43(6):630-5.

210. Bleijenberg N, ten Dam VH, Drubbel I, Numans ME, de Wit NJ, Schuurmans MJ. Treatment Fidelity of an Evidence-Based Nurse-Led Intervention in a Proactive Primary Care Program for Older People. Worldviews on Evidence-Based Nursing. 2016;13(1):75-84.

211. Bertschy S, Bostan C, Meyer T, Pannek J. Medical complications during pregnancy and childbirth in women with SCI in Switzerland. Spinal Cord. 2016;54(3):183-7.

212. Bansal S, Hirdes JP, Maxwell CJ, Papaioannou A, Giangregorio LM. Identifying Fallers among Home Care Clients with Dementia and Parkinson's Disease. Canadian Journal on Aging-Revue Canadienne Du Vieillissement. 2016;35(3):319-31.

213. Asemota O, Eldemire-Shearer D, Waldron NK, Standard-Goldson A. Prevalence of Self-reported Urinary Incontinence in Community-dwelling Older Adults of Westmoreland, Jamaica. Medicc Review. 2016;18(1-2):41-5.

214. Al Balushi A, Meeks MW, Hayat G, Kafaie J. Creutzfeldt-Jakob Disease: analysis of Four Cases. Frontiers in Neurology. 2016;7.

215. Weber P, Meluzinova H, Matejovska-Kubesova H, Polcarova V, Jarkovsky J, Bielakova K, et al. Geriatric giants - contemporary occurrence in 12,210 in-patients. Bratislava Medical Journal-Bratislavske Lekarske Listy. 2015;116(7):408-16.

216. Wagg A, Oelke M, Angulo JC, Scholfield D, Arumi D. Review of the Efficacy and Safety of Fesoterodine for Treating Overactive Bladder and Urgency Urinary Incontinence in Elderly Patients. Drugs & Aging. 2015;32(2):103-25.

217. Vale TC, Caramelli P, Cardoso F. Clinicoradiological comparison between vascular parkinsonism and Parkinson's disease. Journal of Neurology Neurosurgery and Psychiatry. 2015;86(5):547-53.

218. Tomioka K, Okamoto N, Morikawa M, Kurumatani N. Self-Reported Hearing Loss Predicts 5-Year Decline in Higher-Level Functional Capacity in High-Functioning Elderly Adults: The Fujiwara-Kyo Study. Journal of the American Geriatrics Society. 2015;63(11):2260-8.

219. Tavsanli NG, Turkmen SN. Fear of falling in elderly people living in a nursing home - perspective from Manisa. Journal of the Pakistan Medical Association. 2015;65(4):418-20.

220. Tannenbaum C, van den Heuvel E, Fritel X, Southall K, Jutai J, Rajabali S, et al. Continence Across Continents To Upend Stigma and Dependency (CACTUS-D): study protocol for a cluster randomized controlled trial. Trials. 2015;16.

221. Shin JH, Hyun TK. Nurse Staffing and Quality of Care of Nursing Home Residents in Korea. Journal of Nursing Scholarship. 2015;47(6):555-64.

222. Schussler S, Lohrmann C. Change in Care Dependency and Nursing Care Problems in Nursing Home Residents with and without Dementia: A 2-Year Panel Study. Plos One. 2015;10(10).

223. Schussler S, Dassen T, Lohrmann C. Comparison of care dependency and related nursing care problems between Austrian nursing home residents with and without dementia. European Geriatric Medicine. 2015;6(1):46-52.

224. Raval AD, Zhou S, Wei WH, Bhattacharjee S, Miao R, Sambamoorthi U. 30-Day Readmission Among Elderly Medicare Beneficiaries with Type 2 Diabetes. Population Health Management. 2015;18(4):256-64.

225. Ramesh B, Anuradha S. STUDY OF OUTCOME OF TOT MESH REPAIR IN GENUINE STRESS INCONTINENCE. Journal of Evolution of Medical and Dental Sciences-Jemds. 2015;4(6):1043-8.

226. Pinar R, Ergun A, Erol S, Kurtulus Z, Gur K, Sert H, et al. THE ADAPTATION OF THE EASY-CARE STANDARD ASSESSMENT INSTRUMENT INTO TURKISH AND EVALUATION OF PSYCHOMETRIC PROPERTIES AMONG TURKISH OLDER PEOPLE. Nobel Medicus. 2015;11(2):85-92.

227. Nuotio M, Luukkaala T. Factors associated with the night-time index fall in an older hip fracture population. European Geriatric Medicine. 2015;6(1):21-5.

228. McArthur C, Hirdes J, Berg K, Giangregorio L. Who Receives Rehabilitation in Canadian Long-Term Care Facilities? A Cross-Sectional Study. Physiotherapy Canada. 2015;67(2):113-21.

229. MacLachlan LS, Rovner ES. New Treatments for Incontinence. Advances in Chronic Kidney Disease. 2015;22(4):279-88.

230. Luo X, Chuang CC, Yang E, Zou KH, Araiza AL, Bhagnani T. Prevalence, management and outcomes of medically complex vulnerable elderly patients with urinary incontinence in the United States. International Journal of Clinical Practice. 2015;69(12):1517-24.

231. Lichtenstein BJ, Reuben DB, Karlamangla AS, Han WJ, Roth CP, Wenger NS. Effect of Physician Delegation to Other Healthcare Providers on the Quality of Care for Geriatric Conditions. Journal of the American Geriatrics Society. 2015;63(10):2164-70.

232. Leland NE, Crum K, Phipps S, Roberts P, Gage B. Advancing the Value and Quality of Occupational Therapy in Health Service Delivery. American Journal of Occupational Therapy. 2015;69(1):16-22.

233. Lalor AF, Brown T, Robins L, Lee DCA, O'Connor D, Russell G, et al. Anxiety and Depression during Transition from Hospital to Community in Older Adults: Concepts of a Study to Explain Late Age Onset Depression. Healthcare. 2015;3(3):478-502.

234. Lahmann NA, Tannen A, Kuntz S, Raeder K, Schmitz G, Dassen T, et al. Mobility is the key! Trends and associations of common care problems in German long-term care facilities from 2008 to 2012. International Journal of Nursing Studies. 2015;52(1):167-74.

235. Kim KH. The role of primary care of voiding dysfunction in rehabilitation and convalescent hospitals. Journal of the Korean Medical Association. 2015;58(6):557-62.

236. Kim H, Yoshida H, Hu XY, Saito K, Yoshida Y, Kim M, et al. Association between Self-Reported Urinary Incontinence and Musculoskeletal Conditions in Community-Dwelling Elderly Women: A Cross-Sectional Study. Neurourology and Urodynamics. 2015;34(4):322-6.

237. Kasikci M, Kilic D, Avsar G, Sirin M. Prevalence of urinary incontinence in older Turkish women, risk factors, and effect on activities of daily living. Archives of Gerontology and Geriatrics. 2015;61(2):217-23.

238. Karter AJ, Laiteerapong N, Chin MH, Moffet HH, Parker MM, Sudore R, et al. Ethnic Differences in Geriatric Conditions and Diabetes Complications Among Older, Insured Adults With Diabetes: The Diabetes and Aging Study. Journal of Aging and Health. 2015;27(5):894-918.

239. Huang LK, Wang YW, Chou CH, Liu YL, Hsieh JG. Application of a World Health Organization 10-minute screening tool in eastern Taiwand-Falls and self-rated health status among community-dwelling elderly. Tzu Chi Medical Journal. 2015;27(3):120-3.

240. Hodges SJ. Non-neurogenic Bladder Dysfunction in Children. Current Bladder Dysfunction Reports. 2015;10(3):245-9.

241. Greene M, Covinsky KE, Valcour V, Miao YH, Madamba J, Lampiris H, et al. Geriatric Syndromes in Older HIV-Infected Adults. Jaids-Journal of Acquired Immune Deficiency Syndromes. 2015;69(2):161-7.

242. Gosch M, Talasz H, Nicholas JA, Kammerlander C, Lechleitner M. Urinary incontinence and poor functional status in fragility fracture patients: an underrecognized and underappreciated association. Archives of Orthopaedic and Trauma Surgery. 2015;135(1):59-67.

243. Goessaert AS, Everaert K, Hoebeke P, Kapila A, Walle JV. Nocturnal enuresis and nocturia, differences and similarities - lessons to learn? Acta Clinica Belgica. 2015;70(2):81-6.

244. Godmaire GC, Grenier S, Tannenbaum C. An Independent Association Between Urinary Incontinence and Falls in Chronic Benzodiazepine Users. Journal of the American Geriatrics Society. 2015;63(5):1035-7.

245. Gnjidic D, Bennett A, Le Couteur DG, Blyth FM, Cumming RG, Waite L, et al. Ischemic heart disease, prescription of optimal medical therapy and geriatric syndromes in community-dwelling older men: A population-based study. International Journal of Cardiology. 2015;192:49-55.

246. Ghinescu M, Olaroiu M, Aurelian S, Halfens RJG, Dumitrescu L, Schols J, et al. Assessment of Care Problems in Romania: Feasibility and Exploration. Journal of the American Medical Directors Association. 2015;16(1).

247. Ghetti C, Lee M, Oliphant S, Okun M, Lowder JL. Sleep quality in women seeking care for pelvic organ prolapse. Maturitas. 2015;80(2):155-61.

248. Edwards R, Hunter K, Wagg A. Lower urinary tract symptoms and falls in older women: A case control study. Maturitas. 2015;80(3):308-11.

249. Diabate I, Sow I, Ba A, Fall V. Indications of urological surgery by vagina route: A series of 30 cases. African Journal of Urology. 2015;21(1):30-5.

250. de Oliveira MF, Reis RC, Trindade EM, Pinto FCG. Evidences in the treatment of idiopathic normal pressure hydrocephalus. Revista Da Associacao Medica Brasileira. 2015;61(3):258-62.

251. Clerencia-Sierra M, Calderon-Larranaga A, Martinez-Velilla N, Vergara-Mitxeltorena I, Aldaz-Herce P, Poblador-Plou B, et al. Multimorbidity Patterns in Hospitalized Older Patients: Associations among Chronic Diseases and Geriatric Syndromes. Plos One. 2015;10(7).

252. Carlson C, Merel SE, Yukawa M. Geriatric Syndromes and Geriatric Assessment for the Generalist. Medical Clinics of North America. 2015;99(2):263-+.

253. Bertapelle MP, Vottero M, Del Popolo G, Mencarini M, Ostardo E, Spinelli M, et al. Sacral neuromodulation and Botulinum toxin A for refractory idiopathic overactive bladder: a cost-utility analysis in the perspective of Italian Healthcare System. World Journal of Urology. 2015;33(8):1109-17.

254. Barkhausen T, Junius-Walker U, Hummers-Pradier E, Mueller CA, Theile G. "It's MAGIC" - development of a manageable geriatric assessment for general practice use. Bmc Family Practice. 2015;16.

255. Ajmera M, Raval A, Zhou S, Wei WH, Bhattacharya R, Pan CS, et al. A Real-World Observational Study of Time to Treatment Intensification Among Elderly Patients with Inadequately Controlled Type 2 Diabetes Mellitus. Journal of Managed Care & Specialty Pharmacy. 2015;21(12):1184-+.

256. Abreu HCD, Reiners AAO, Azevedo RCD, da Silva AMC, Abreu D, de Oliveira AD. Incidence and predicting factors of falls of older inpatients. Revista De Saude Publica. 2015;49.

257. Abraha I, Cruz-Jentoft A, Soiza RL, O'Mahony D, Cherubini A. Evidence of and recommendations for non-pharmacological interventions for common geriatric conditions: the SENATOR-ONTOP systematic review protocol. Bmj Open. 2015;5(1).

258. Wilkerson LM, Iwata I, Wilkerson MD, Heflin MT. An Educational Intervention to Improve Internal Medicine Interns' Awareness of Hazards of Hospitalization in Acutely Ill Older Adults. Journal of the American Geriatrics Society. 2014;62(4):727-33.

259. Wiedemann A, Anding R, Kirschner-Hermanns R. Characteristics of urinary incontinence in the elderly. Urologe. 2014;53(10):1543-50.

260. Wang HH, Sheu JT, Shyu YIL, Chang HY, Li CL. Geriatric conditions as predictors of increased number of hospital admissions and hospital bed days over one year: Findings of a nationwide cohort of older adults from Taiwan. Archives of Gerontology and Geriatrics. 2014;59(1):169-74.

261. Stewart TV, Loskutova N, Galliher JM, Warshaw GA, Coombs LJ, Staton EW, et al. Practice Patterns, Beliefs, and Perceived Barriers to Care Regarding Dementia: A Report from the American Academy of Family Physicians (AAFP) National Research Network. Journal of the American Board of Family Medicine. 2014;27(2):275-83.

262. Smith C, Finan M, Axelband J, Williams K. It is not a tumor: a rare case of tumefactive multiple sclerosis. American Journal of Emergency Medicine. 2014;32(8).

263. Siddiqui NY, Levin PJ, Phadtare A, Pietrobon R, Ammarell N. Perceptions about female urinary incontinence: a systematic review. International Urogynecology Journal. 2014;25(7):863-71.

264. Sammels M, Vandesande J, Vlaeyen E, Peerlinck K, Milisen K. Falling and fall risk factors in adults with haemophilia: an exploratory study. Haemophilia. 2014;20(6):836-45.

265. Rendtorff R, Knispel HH, Tunn R. Rupture of the Left Renal Fornix after Vaginal Repair of Postpartum Vesicovaginal Fistula. Geburtshilfe Und Frauenheilkunde. 2014;74(4):376-8.

266. Rafiq M, McGovern A, Jones S, Harris K, Tomson C, Gallagher H, et al. Falls in the elderly were predicted opportunistically using a decision tree and systematically using a database-driven screening tool. Journal of Clinical Epidemiology. 2014;67(8):877-86.

267. Pileggi C, Manuti B, Costantino R, Bianco A, Nobile CGA, Pavia M. Quality of Care in One Italian Nursing Home Measured by ACOVE Process Indicators. Plos One. 2014;9(3).

268. Min LL, Kerr EA, Blaum CS, Reuben D, Cigolle C, Wenger N. Contrasting Effects of Geriatric Versus General Medical Multimorbidity on Quality of Ambulatory Care. Journal of the American Geriatrics Society. 2014;62(9):1714-21.

269. Logan BA, Correia K, McCarthy J, Slattery MJ. Voiding dysfunction related to adverse childhood experiences and neuropsychiatric disorders. Journal of Pediatric Urology. 2014;10(4):634-8.

270. Lino VTS, Portela MC, Camacho LAB, Rodrigues NCP. Reliability of screening tests for health-related problems among low-income elderly. Cadernos De Saude Publica. 2014;30(12):2691-6.

271. Lineberry C, Stein DE. Infection, Sepsis, and Immune Function in the Older Adult Receiving Critical Care. Critical Care Nursing Clinics of North America. 2014;26(1):47-+.

272. Lim SC, Mamun K, Lim JKH. Comparison between elderly inpatient fallers with and without dementia. Singapore Medical Journal. 2014;55(2):67-71.

273. Liang CK, Chou MY, Peng LN, Liao MC, Chu CL, Lin YT, et al. Gait speed and risk assessment for falls among men aged 80 years and older: A prospective cohort study in Taiwan. European Geriatric Medicine. 2014;5(5):298-302.

274. Lahmann NA, Heinze C, Rommel A. Falls in German hospitals and nursing homes 2006-2013. Frequencies, injuries, risk assessment, and preventive measures. Bundesgesundheitsblatt-Gesundheitsforschung-Gesundheitsschutz. 2014;57(6):650-9.

275. Khan RB, Hudson MM, Ledet DS, Morris EB, Pui CH, Howard SC, et al. Neurologic morbidity and quality of life in survivors of childhood acute lymphoblastic leukemia: a prospective cross-sectional study. Journal of Cancer Survivorship. 2014;8(4):688-96.

276. Kamiya M, Sakurai T, Ogama N, Maki Y, Toba K. Factors associated with increased caregivers' burden in several cognitive stages of Alzheimer's disease. Geriatrics & Gerontology International. 2014;14:45-55.

277. Josephs KA, Duffy JR, Strand EA, Machulda MM, Senjem ML, Gunter JL, et al. The evolution of primary progressive apraxia of speech. Brain. 2014;137:2783-95.

278. Inouye BM, Shah BB, Massanyi EZ, Di Carlo HN, Kern AJ, Tourchi A, et al. Urologic complications of major genitourinary reconstruction in the exstrophy-epispadias complex. Journal of Pediatric Urology. 2014;10(4):680-7.

279. Hofmann H, Hahn S. Characteristics of nursing home residents and physical restraint: a systematic literature review. Journal of Clinical Nursing. 2014;23(21-22):3012-24.

280. Haab F. Chapter 1: The Conditions of Neurogenic Detrusor Overactivity and Overactive Bladder. Neurourology and Urodynamics. 2014;33:S2-S5.

281. Glazener CMA, MacArthur C, Hagen S, Elders A, Lancashire R, Herbison GP, et al. Twelve-year follow-up of conservative management of postnatal urinary and faecal incontinence and prolapse outcomes: randomised controlled trial. Bjog-an International Journal of Obstetrics and Gynaecology. 2014;121(1):112-20.

282. Garely AD, Noor N. Diagnosis and Surgical Treatment of Stress Urinary Incontinence. Obstetrics and Gynecology. 2014;124(5):1011-27.

283. Fraser SA, Elliott V, de Bruin ED, Bherer L, Dumoulin C. The Effects of Combining Videogame Dancing and Pelvic Floor Training to Improve Dual-Task Gait and Cognition in Women with Mixed-Urinary Incontinence. Games for Health Journal. 2014;3(3):172-8.

284. Frainey BT, Yerkes EB, Menon VS, Gong EM, Meyer TA, Bowman RM, et al. Predictors of urinary continence following tethered cord release in children with occult spinal dysraphism. Journal of Pediatric Urology. 2014;10(4):627-33.

285. Dellaroza MSG, Pimenta CAD, Lebrao ML, Duarte YAD, Braga PE. Association between chronic pain and self-reported falls in the SABE study population. Cadernos De Saude Publica. 2014;30(3):522-32.

286. Choi EJ, Kim SA, Kim NR, Rhee JA, Yun YW, Shin MH. Risk Factors for Falls in Older Korean Adults: The 2011 Community Health Survey. Journal of Korean Medical Science. 2014;29(11):1482-7.

287. Bresee C, Dubina ED, Khan AA, Sevilla C, Grant D, Eilber KS, et al. Prevalence and Correlates of Urinary Incontinence Among Older Community-Dwelling Women. Female Pelvic Medicine and Reconstructive Surgery. 2014;20(6):328-33.

288. Bhidayasiri R, Jitkritsadakul O, Petchrutchatachart S, Kaewwilai L, Panyakaew P, Boonrod N, et al. Nocturnal manifestations of atypical and vascular parkinsonism: How do they differ from Parkinson's disease? Journal of Neural Transmission. 2014;121:S69-S77.

289. Berry A, Rudick K, Richter M, Zderic S. Objective versus subjective outcome measures of biofeedback: What really matters? Journal of Pediatric Urology. 2014;10(4):620-6.

290. Bambi S, Lucchini A, Solaro M, Lumini E, Rasero L. Interventional Patient Hygiene Model. A critical reflection on basic nursing care in intensive care units. Assistenza Infermieristica E Ricerca. 2014;33(2):90-6.

291. Avelino-Silva TJ, Farfel JM, Curiati JAE, Amaral JRG, Campora F, Jacob W. Comprehensive geriatric assessment predicts mortality and adverse outcomes in hospitalized older adults. Bmc Geriatrics. 2014;14.

292. Andreasson A, Fall M, Persson E, Stranne J, Peeker R. High revision rate following artificial urethral sphincter implantation. Scandinavian Journal of Urology. 2014;48(6):544-8.

293. Allain TJ, Mwambelo M, Mdolo T, Mfune P. Falls and other geriatric syndromes in Blantyre, Malawi: a community survey of older adults. Malawi Medical Journal. 2014;26(4):105-8.

294. Abreu HCD, Reiners AAO, Azevedo RCD, da Silva AMC, Abreu D. Urinary incontinence in the prediction of falls in hospitalized elderly. Revista Da Escola De Enfermagem Da Usp. 2014;48(5):848-53.

295. Yoshida M, Murayama R, Ota E, Nakata M, Kozuma S, Homma Y. Reliability and validity of the Japanese version of the pelvic floor distress inventory-short form 20. International Urogynecology Journal. 2013;24(6):1039-46.

296. van Nie-Visser NC, Schols J, Meesterberends E, Lohrmann C, Meijers JMM, Halfens RJG. An International prevalence measurement of care problems: study protocol. Journal of Advanced Nursing. 2013;69(9):E18-E29.

297. van Bokhorst-de van der Schueren MAE, Lonterman-Monasch S, de Vries OJ, Danner SA, Kramer MHH, Muller M. Prevalence and determinants for malnutrition in geriatric outpatients. Clinical Nutrition. 2013;32(6):1007-11.

298. Vale TC, Caramelli P, Cardoso F. Vascular parkinsonism: a case series of 17 patients. Arquivos De Neuro-Psiquiatria. 2013;71(10):757-62.

299. Tyagi V, Perera M, Guerrero K. Trends in obstetric anal sphincter injuries over 10 years. Journal of Obstetrics and Gynaecology. 2013;33(8):844-9.

300. Tikkinen KAO, Agarwal A, Griebling TL. Epidemiology of male urinary incontinence. Current Opinion in Urology. 2013;23(6):502-8.

301. Spears GV, Roth CP, Miake-Lye IM, Saliba D, Shekelle PG, Ganz DA. Redesign of an Electronic Clinical Reminder to Prevent Falls in Older Adults. Medical Care. 2013;51(3):S37-S43.

302. Sorbye LW, Grue EV. Hip fracture and urinary incontinence - use of indwelling catheter postsurgery. Scandinavian Journal of Caring Sciences. 2013;27(3):632-42.

303. Seino S, Yabushita N, Kim M, Nemoto M, Jung S, Osuka Y, et al. Physical performance measures as a useful indicator of multiple geriatric syndromes in women aged 75 years and older. Geriatrics & Gerontology International. 2013;13(4):901-10.

304. Ruggero CR, Bilton TL, Teixeira LF, Ramos JDA, Alouche SR, Dias RC, et al. Gait speed correlates in a multiracial population of community-dwelling older adults living in Brazil: a cross-sectional population-based study. Bmc Public Health. 2013;13.

305. Rosso AL, Eaton CB, Wallace R, Gold R, Stefanick ML, Ockene JK, et al. Geriatric Syndromes and Incident Disability in Older Women: Results from the Women's Health Initiative Observational Study. Journal of the American Geriatrics Society. 2013;61(3):371-9.

306. Reuben DB, Ganz DA, Roth CP, McCreath HE, Ramirez KD, Wenger NS. Effect of Nurse Practitioner Comanagement on the Care of Geriatric Conditions. Journal of the American Geriatrics Society. 2013;61(6):857-67.

307. Priddis H, Dahlen HG, Schmied V, Sneddon A, Kettle C, Brown C, et al. Risk of recurrence, subsequent mode of birth and morbidity for women who experienced severe perineal trauma in a first birth in New South Wales between 2000-2008: a population based data linkage study. Bmc Pregnancy and Childbirth. 2013;13.

308. Popejoy LL, Marek KD, Scott-Cawiezell J. Patterns and Problems Associated with Transitions After Hip Fracture in Older Adults. Journal of Gerontological Nursing. 2013;39(9):43-52.

309. Oral A, Kucukdeveci A, Varela E, Ilieva EM, Valero R, Berteanu M, et al. Osteoporosis. The role of Physical and Rehabilitation Medicine Physicians. The European perspective based on the best evidence. European Journal of Physical and Rehabilitation Medicine. 2013;49(4):565-77.

310. Oliphant SS, Ghetti C, McGough RL, Wang L, Bunker CH, Lowder JL. Inpatient procedures in elderly women: An analysis over time. Maturitas. 2013;75(4):349-54.

311. Notz HJ, Hautumm B, Werdier D, Groves R, Odenthal KP. Trospium chloride once daily for overactive bladder syndrome. Results of a multicenter observational study. Urologe. 2013;52(1):65-70.

312. Magdalinou NK, Ling H, Smith JDS, Schott JM, Watkins LD, Lees AJ. Normal pressure hydrocephalus or progressive supranuclear palsy? A clinicopathological case series. Journal of Neurology. 2013;260(4):1009-13.

313. Lu FP, Chan DC, Kuo HK, Wu SC. Sex differences in the impact of diabetes on the risk of geriatric conditions. Geriatrics & Gerontology International. 2013;13(1):116-22.

314. Kashyap M, Tu LM, Tannenbaum C. Prevalence of commonly prescribed medications potentially contributing to urinary symptoms in a cohort of older patients seeking care for incontinence. Bmc Geriatrics. 2013;13.

315. Imayama I, Alfano CM, Neuhouser ML, George SM, Smith AW, Baumgartner RN, et al. Weight, inflammation, cancer-related symptoms and health-related quality of life among breast cancer survivors. Breast Cancer Research and Treatment. 2013;140(1):159-76.

316. Hussain A, Pansota MS, Rasool M, Tabassum SA, Ahmad I, Saleem MS. Outcome of End-to-End Urethroplasty in Post-traumatic Stricture of Posterior Urethra. Jcpsp-Journal of the College of Physicians and Surgeons Pakistan. 2013;23(4):272-5.

317. Hunter KF, Voaklander D, Hsu ZY, Moore KN. Lower urinary tract symptoms and falls risk among older women receiving home support: a prospective cohort study. Bmc Geriatrics. 2013;13.

318. Hersh L, Salzman B. Clinical Management of Urinary Incontinence in Women. American Family Physician. 2013;87(9):634-40.

319. Hedman AMR, Fonad E, Sandmark H. Older people living at home: associations between falls and health complaints in men and women. Journal of Clinical Nursing. 2013;22(19-20):2945-52.

320. Gnjidic D, Johnell K. Clinical implications from drug-drug and drug-disease interactions in older people. Clinical and Experimental Pharmacology and Physiology. 2013;40(5):320-5.

321. Fritel X, Lachal L, Cassou B, Fauconnier A, Dargent-Molina P. Mobility impairment is associated with urge but not stress urinary incontinence in community-dwelling older women: results from the Ossebo study. Bjog-an International Journal of Obstetrics and Gynaecology. 2013;120(12):1566-74.

322. Damian J, Pastor-Barriuso R, Valderrama-Gama E, de Pedro-Cuesta J. Factors associated with falls among older adults living in institutions. Bmc Geriatrics. 2013;13.

323. Coyne KS, Wein A, Nicholson S, Kvasz M, Chen CI, Milsom I. Comorbidities and personal burden of urgency urinary incontinence: a systematic review. International Journal of Clinical Practice. 2013;67(10):1015-33.

324. Cegelka A, Amer Geriatrics Soc Expert P. Guidelines Abstracted from the American Geriatrics Society Guidelines for Improving the Care of Older Adults with Diabetes Mellitus: 2013 Update. Journal of the American Geriatrics Society. 2013;61(11):2020-6.

325. Brown RT, Kiely DK, Bharel M, Mitchell SL. Factors Associated with Geriatric Syndromes in Older Homeless Adults. Journal of Health Care for the Poor and Underserved. 2013;24(2):456-68.

326. Bouchard D, Abramowitz L, Castinel A, Suduca JM, Staumont G, Soudan D, et al. One-year outcome of haemorrhoidectomy: a prospective multicentre French study. Colorectal Disease. 2013;15(6):719-26.

327. Booth J, Paul L, Rafferty D, MacInnes C. The relationship between urinary bladder control and gait in women. Neurourology and Urodynamics. 2013;32(1):43-7.

328. Berardelli M, De Rango F, Morelli M, Corsonello A, Mazzei B, Mari V, et al. Urinary Incontinence in the Elderly and in the Oldest Old: Correlation with Frailty and Mortality. Rejuvenation Research. 2013;16(3):206-11.

329. Batchelor FA, Dow B, Low MA. Do continence management strategies reduce falls? A systematic review. Australasian Journal on Ageing. 2013;32(4):211-6.

330. Arnold PM, Teuber J. Marfan syndrome and symptomatic sacral cyst: Report of two cases. Journal of Spinal Cord Medicine. 2013;36(5):499-503.

331. Zou BJ, Zhang YL, Li YC, Wang ZT, Zhang P, Zhang XY, et al. Survey of spinal cord injury-induced neurogenic bladder studies using the Web of Science. Neural Regeneration Research. 2012;7(23):1832-9.

332. Wong LY, Heng BH, Ng CWL, Molina JAD, George PP, Cheah JTS. Geriatric Syndromes and Depressed Mood in Lower-income Singaporeans with Diabetes: Implications for Diabetes Management and Health Promotion. Annals Academy of Medicine Singapore. 2012;41(2):67-76.

333. Stubendorff K, Aarsland D, Minthon L, Londos E. The Impact of Autonomic Dysfunction on Survival in Patients with Dementia with Lewy Bodies and Parkinson's Disease with Dementia. Plos One. 2012;7(10).

334. Roth CP, Ganz DA, Nickels L, Martin D, Beckman R, Wenger NS. Nurse Care Manager Contribution to Quality of Care in a Dual-Eligible Special Needs Plan. Journal of Gerontological Nursing. 2012;38(7):44-54.

335. Perera M, Jones B, O'Brien M, Hutson JM. Long-Term Urethral Function Measured by Uroflowmetry After Hypospadias Surgery: Comparison with an Age Matched Control. Journal of Urology. 2012;188(4):1457-62.

336. Onder G, Carpenter I, Finne-Soveri H, Gindin J, Frijters D, Henrard JC, et al. Assessment of nursing home residents in Europe: the Services and Health for Elderly in Long TERm care (SHELTER) study. Bmc Health Services Research. 2012;12.

337. Miller KL, Baraldi CA. Geriatric gynecology: promoting health and avoiding harm. American Journal of Obstetrics and Gynecology. 2012;207(5):355-67.

338. Mahler M, Sarvimaki A. Fear of falling from a daily life perspective; narratives from later life. Scandinavian Journal of Caring Sciences. 2012;26(1):38-44.

339. LoGiudice DC, Smith K, Atkinson D, Dwyer A, Lautenschlager N, Almeida OA, et al. Preliminary evaluation of the prevalence of falls, pain and urinary incontinence in remote living Indigenous Australians over the age of 45 years. Internal Medicine Journal. 2012;42(6):E102-E7.

340. Liu JM, Meng FX, Liu ZL. Seizure-related adverse events during video-electroencephalography monitoring. Epileptic Disorders. 2012;14(1):51-6.

341. Lamartina C. Expert's comment concerning Grand Rounds case entitled "Limited access surgery for 360 degrees in situ fusion in a dysraphic patient with high grade spondylolisthesis" (by M. A. Konig and B. M. Boszczyk). European Spine Journal. 2012;21(3):396-9.

342. Kim HJ, Chun MH, Han EY, Yi JH, Kim DK. The utility of a bladder scan protocol using a portable ultrasonographic device in subacute stroke patients. Disability and Rehabilitation. 2012;34(6):486-90.

343. Jacobs JM, Maaravi Y, Cohen A, Bursztyn M, Ein-Mor E, Stessman J. Changing Profile of Health and Function from Age 70 to 85 Years. Gerontology. 2012;58(4):313-21.

344. Heinze C, Dassen T, Grittner U. Use of physical restraints in nursing homes and hospitals and related factors: a cross-sectional study. Journal of Clinical Nursing. 2012;21(7-8):1033-40.

345. Groshaus H, Boscan A, Khandwala F, Holroyd-Leduc J. Use of Clinical Decision Support to Improve the Quality of Care Provided to Older Hospitalized Patients. Applied Clinical Informatics. 2012;3(1):94-102.

346. Griebling TL. Re: Does Better Quality of Care for Falls and Urinary Incontinence Result in Better Participant-Reported Outcomes? Editorial Comment. Journal of Urology. 2012;187(3):967-8.

347. Griebling TL. Re: Association Between the Geriatric Giants of Urinary Incontinence and Falls in Older People Using Data from the Leicestershire MRC Incontinence Study Editorial Comment. Journal of Urology. 2012;188(1):225-6.

348. Griebling TL. Re: Urinary Incontinence: An Under-Recognized Risk Factor for Falls Among Elderly Dementia Patients Editorial Comment. Journal of Urology. 2012;188(4):1215-.

349. Grandstaff M, Lyons D. Impact of a Continence Training Program on Patient Safety and Quality. Rehabilitation Nursing. 2012;37(4):180-4.

350. Gobeaux N, Yates DR, Denys P, Even-Schneider A, Richard F, Chartier-Kastler E. Supratrigonal cystectomy with hautmann pouch as treatment for neurogenic bladder in spinal cord injury patients: Long-term functional results. Neurourology and Urodynamics. 2012;31(5):672-6.

351. Glass PG, Lees AJ, Bacellar A, Zijlmans J, Katzenschlager R, Silveira-Moriyama L. The clinical features of pathologically confirmed vascular Parkinsonism. Journal of Neurology Neurosurgery and Psychiatry. 2012;83(10):1027-9.

352. Foley AL, Loharuka S, Barrett JA, Mathews R, Williams K, McGrother CW, et al. Association between the Geriatric Giants of urinary incontinence and falls in older people using data from the Leicestershire MRC Incontinence Study. Age and Ageing. 2012;41(1):35-40.

353. Choi K, Park E, Lee IS. Homebound status and Related Factors According to Age in Female Elders in the Community. Journal of Korean Academy of Nursing. 2012;42(2):291-301.

354. Castrejon-Perez RC, Borges-Yanez SA, Gutierrez-Robledo LM, Avila-Funes JA. Oral health conditions and frailty in Mexican community-dwelling elderly: a cross sectional analysis. Bmc Public Health. 2012;12.

355. Casazza BA. Diagnosis and Treatment of Acute Low Back Pain. American Family Physician. 2012;85(4):343-50.

356. Brown RT, Kiely DK, Bharel M, Mitchell SL. Geriatric Syndromes in Older Homeless Adults. Journal of General Internal Medicine. 2012;27(1):16-22.

357. Arai H, Ouchi Y, Yokode M, Ito H, Uematsu H, Eto F, et al. Toward the realization of a better aged society: Messages from gerontology and geriatrics. Geriatrics & Gerontology International. 2012;12(1):16-22.

358. Alemdaroglu E, Ucan H, Topcuoglu AM, Sivas F. In-Hospital Predictors of Falls in Community-Dwelling Individuals After Stroke in the First 6 Months After a Baseline Evaluation: A Prospective Cohort Study. Archives of Physical Medicine and Rehabilitation. 2012;93(12):2244-50.

359. Akman YR, Basari MM. UROLOGIC PROBLEMS IN THE ELDERLY POPULATION. Turkish Journal of Geriatrics-Turk Geriatri Dergisi. 2012;15(4):455-62.

360. Zuck P, Allaert FA. Acute dry coughs have underestimated impacts on patients' everyday lives. Revue De Pneumologie Clinique. 2011;67(5):304-8.

361. Wennberg AL, Altman D, Lundholm C, Klint A, Iliadou A, Peeker R, et al. Genetic Influences Are Important for Most But Not All Lower Urinary Tract Symptoms: A Population-Based Survey in a Cohort of Adult Swedish Twins. European Urology. 2011;59(6):1032-8.

362. Wenger NS, Roth CP, Martin D, Nickels L, Beckman R, Kamberg C, et al. Quality of Care Provided in a Special Needs Plan Using a Nurse Care Manager Model. Journal of the American Geriatrics Society. 2011;59(10):1810-22.

363. Vergara MT, Suarez J, Orellana H, Cofre P, Germain F, Stanley W, et al. Fecal incontinence in adults. Revista Chilena De Cirugia. 2011;63(3):320-6.

364. Tubaro A, Koelbl H, Laterza R, Khullar V, de Nunzio C. Ultrasound Imaging of the Pelvic Floor: Where Are We Going? Neurourology and Urodynamics. 2011;30(5):729-34.

365. Sran MM. Filling in the Gaps Before Clients Fall Through the Cracks: Physiotherapists Have the Skills to Help Clients Preserve Bone and Prevent Falls. Physiotherapy Canada. 2011;63(3):261-2.

366. Rosso AL, Eaton CB, Wallace R, Gold R, Curb JD, Stefanick ML, et al. Combined Impact of Geriatric Syndromes and Cardiometabolic Diseases on Measures of Functional Impairment. Journals of Gerontology Series a-Biological Sciences and Medical Sciences. 2011;66(3):349-54.

367. Rosen SL, Reuben DB. Geriatric Assessment Tools. Mount Sinai Journal of Medicine. 2011;78(4):489-97.

368. Pal L, Hailpern SM, Santoro NF, Freeman R, Barad D, Kipersztok S, et al. Increased incident hip fractures in postmenopausal women with moderate to severe pelvic organ prolapse. Menopause-the Journal of the North American Menopause Society. 2011;18(9):967-73.

369. Mohile SG, Fan L, Reeve E, Jean-Pierre P, Mustian K, Peppone L, et al. Association of Cancer With Geriatric Syndromes in Older Medicare Beneficiaries. Journal of Clinical Oncology. 2011;29(11):1458-64.

370. Min LC, Reuben DB, Keeler E, Ganz DA, Fung CH, Shekelle P, et al. Is Patient-Perceived Severity of a Geriatric Condition Related to Better Quality of Care? Medical Care. 2011;49(1):101-7.

371. Min LC, Reuben DB, Adams J, Shekelle PG, Ganz DA, Roth CP, et al. Does Better Quality of Care for Falls and Urinary Incontinence Result in Better Participant-Reported Outcomes? Journal of the American Geriatrics Society. 2011;59(8):1435-43.

372. Merola A, Zibetti M, Angrisano S, Rizzi L, Ricchi V, Artusi CA, et al. Parkinson's disease progression at 30 years: a study of subthalamic deep brain-stimulated patients. Brain. 2011;134:2074-84.

373. Makaroff L, Gunn A, Gervasoni C, Richy F. Gastrointestinal Disorders in Parkinson's Disease: Prevalence and Health Outcomes in a US Claims Database. Journal of Parkinsons Disease. 2011;1(1):65-74.

374. Lucchetti G, Granero AL. Use of comprehensive geriatric assessment in general practice: Results from the 'Senta Pua' project in Brazil. European Journal of General Practice. 2011;17(1):20-7.

375. Limpawattana P, Sawanyawisuth K, Soonpornrai S, Huangthaisong W. Prevalence and recognition of geriatric syndromes in an outpatient clinic at a tertiary care hospital of Thailand. Asian Biomedicine. 2011;5(4):493-7.

376. Lee CY, Chen LK, Lo YK, Liang CK, Chou MY, Lo CC, et al. Urinary Incontinence: An Under-Recognized Risk Factor for Falls Among Elderly Dementia Patients. Neurourology and Urodynamics. 2011;30(7):1286-90.

377. Lakhan P, Jones M, Wilson A, Courtney M, Hirdes J, Gray LC. A Prospective Cohort Study of Geriatric Syndromes Among Older Medical Patients Admitted to Acute Care Hospitals. Journal of the American Geriatrics Society. 2011;59(11):2001-8.

378. Lago RB, Dieguez IM, Caro FA. Lumbar spinal cord injury without radiological abnormality in a child: an exceptional form of presentation. Archivos Argentinos De Pediatria. 2011;109(3):E47-E51.

379. Kim H, Yoshida H, Suzuki T. The effects of multidimensional exercise on functional decline, urinary incontinence, and fear of falling in community-dwelling elderly women with multiple symptoms of geriatric syndrome: A randomized controlled and 6-month follow-up trial. Archives of Gerontology and Geriatrics. 2011;52(1):99-105.

380. Idiaquez J, Roman GC. Autonomic dysfunction in neurodegenerative dementias. Journal of the Neurological Sciences. 2011;305(1-2):22-7.

381. Harlein J, Halfens RJG, Dassen T, Lahmann NA. Falls in older hospital inpatients and the effect of cognitive impairment: a secondary analysis of prevalence studies. Journal of Clinical Nursing. 2011;20(1-2):175-83.

382. Hammouda EI. Overcoming barriers to diabetes control in geriatrics. International Journal of Clinical Practice. 2011;65(4):420-4.

383. Gomes T, Juurlink DN, Ho JMW, Schneeweiss S, Mamdani MM. Risk of Serious Falls Associated With Oxybutynin and Tolterodine: A Population Based Study. Journal of Urology. 2011;186(4):1340-4.

384. Ginsberg DA, Oefelein MG, Ellsworth PI. Once-Daily Administration of Trospium Chloride Extended Release Provides 24-hr Coverage of Nocturnal and Diurnal Symptoms of Overactive Bladder: An Integrated Analysis of Two Phase III Trials. Neurourology and Urodynamics. 2011;30(4):563-7.

385. Elsawy B, Higgins KE. The Geriatric Assessment. American Family Physician. 2011;83(1):48-56.

386. Edwards R, Martin FC, Grant R, Lowe D, Potter J, Husk J, et al. Is urinary continence considered in the assessment of older people after a fall in England and Wales? Cross-sectional clinical audit results. Maturitas. 2011;69(2):179-83.

387. Dogan A, Ceceli E, Okumus M, Gokkaya NKO, Kutsal YG, Borman P, et al. Identifying the Characteristics of Geriatric Patients who Referred to Outpatient Clinics of Physical Medicine and Rehabilitation: A Multicenter Descriptive Study. Turkiye Fiziksel Tip Ve Rehabilitasyon Dergisi-Turkish Journal of Physical Medicine and Rehabilitation. 2011;57(3):143-9.

388. Divani AA, Majidi S, Barrett AM, Noorbaloochi S, Luft AR. Consequences of Stroke in Community-Dwelling Elderly The Health and Retirement Study, 1998 to 2008. Stroke. 2011;42(7):1821-5.

389. Cortes AR, Villarreal E, Galicia L, Martinez L, Vargas ER. Cross sectional geriatric assessment of Mexican older people. Revista Medica De Chile. 2011;139(6):725-31.

390. Cigolle CT, Lee PG, Langa KM, Lee YY, Tian ZY, Blaum CS. Geriatric Conditions Develop in Middle-Aged Adults with Diabetes. Journal of General Internal Medicine. 2011;26(3):272-9.

391. Chang CI, Chan DC, Kuo KN, Hsiung CA, Chen CY. Prevalence and Correlates of Geriatric Frailty in a Northern Taiwan Community. Journal of the Formosan Medical Association. 2011;110(4):247-57.

392. Boyd CM, Leff B, Wolff JL, Yu QL, Zhou J, Rand C, et al. Informing Clinical Practice Guideline Development and Implementation: Prevalence of Coexisting Conditions Among Adults with Coronary Heart Disease. Journal of the American Geriatrics Society. 2011;59(5):797-805.

393. Arlandis S, Castro D, Errando C, Fernandez E, Jimenez M, Gonzalez P, et al. Cost-Effectiveness of Sacral Neuromodulation Compared to Botulinum Neurotoxin A or Continued Medical Management in Refractory Overactive Bladder. Value in Health. 2011;14(2):219-28.

394. Altman D, Lundholm C, Milsom I, Peeker R, Fall M, Iliadou AN, et al. The Genetic and Environmental Contribution to the Occurrence of Bladder Pain Syndrome: An Empirical Approach in a Nationwide Population Sample. European Urology. 2011;59(2):280-5.

395. Zhang AJ, Yu XJ, Wang M. The clinical manifestations and pathophysiology of cerebral small vessel disease. Neuroscience Bulletin. 2010;26(3):257-64.

396. Yalcin I, Peng GB, Viktrup L, Bump RC. Reductions in Stress Urinary Incontinence Episodes: What Is Clinically Important for Women? Neurourology and Urodynamics. 2010;29(3):344-7.

397. Wu JY, He HC, Chen SW, Jin XD, Zhou YX. Surgical therapies of female stress urinary incontinence: experience in 228 cases. International Urogynecology Journal. 2010;21(6):645-9.

398. Westmoreland GR, Counsell SR, Tu WZ, Wu JW, Litzelman DK. Web-Based Training in Geriatrics for Medical Residents: A Randomized Controlled Trial Using Standardized Patients to Assess Outcomes. Journal of the American Geriatrics Society. 2010;58(6):1163-9.

399. Wenger NS, Roth CP, Hall WJ, Ganz DA, Snow V, Byrkit J, et al. Practice Redesign to Improve Care for Falls and Urinary Incontinence Primary Care Intervention for Older Patients. Archives of Internal Medicine. 2010;170(19):1765-72.

400. Warshaw GA, Modawal A, Kues J, Moore I, Margolin G, Sehgal M, et al. Community Physician Education in Geriatrics: Applying the Assessing Care of Vulnerable Elders Model with a Multisite Primary Care Group. Journal of the American Geriatrics Society. 2010;58(9):1780-5.

401. Wang J, Chang LH, Eberly LE, Virnig BA, Kane RL. Cognition Moderates the Relationship Between Facility Characteristics, Personal Impairments, and Nursing Home Residents' Activities of Daily Living. Journal of the American Geriatrics Society. 2010;58(12):2275-83.

402. Wagg A, Verdejo C, Molander U. Review of cognitive impairment with antimuscarinic agents in elderly patients with overactive bladder. International Journal of Clinical Practice. 2010;64(9):1279-86.

403. Vaughan CP, Brown CJ, Goode PS, Burgio KL, Allman RM, Johnson TM. The association of nocturia with incident falls in an elderly community-dwelling cohort. International Journal of Clinical Practice. 2010;64(5):577-83.

404. Unwin BK, Porvaznik M, Spoelhof GD. Nursing Home Care: Part II. Clinical Aspects. American Family Physician. 2010;81(10):1229-37.

405. Smith K, Flicker L, Dwyer A, Atkinson D, Almeida OP, Lautenschlager NT, et al. Factors associated with dementia in Aboriginal Australians. Australian and New Zealand Journal of Psychiatry. 2010;44(10):888-93.

406. Shaw G, Ioannou I, Gelister J, Briggs T. Management of Vesico-Urethral Anastomosis Disruption after Radical Prostatectomy. Urologia Internationalis. 2010;84(4):436-9.

407. Oliver D, Healey F, Haines TP. Preventing Falls and Fall-Related Injuries in Hospitals. Clinics in Geriatric Medicine. 2010;26(4):645-+.

408. Mahajan ST, Patel PB, Marrie RA. Under Treatment of Overactive Bladder Symptoms in Patients With Multiple Sclerosis: An Ancillary Analysis of the NARCOMS Patient Registry. Journal of Urology. 2010;183(4):1432-7.

409. Luo HB, Fang XM, Liao YL, Elliott A, Zhang XZ. Associations of Special Care Units and Outcomes of Residents With Dementia: 2004 National Nursing Home Survey. Gerontologist. 2010;50(4):509-18.

410. Kraus SR, Bavendam T, Brake T, Griebling TL. Vulnerable Elderly Patients and Overactive Bladder Syndrome. Drugs & Aging. 2010;27(9):697-713.

411. King VG, Boyles SH, Worstell TR, Zia J, Clark AL, Gregory WT. Using the Brink score to predict postpartum anal incontinence. American Journal of Obstetrics and Gynecology. 2010;203(5).

412. Jacklin P, Duckett J, Renganathan A. Analytic model comparing the cost utility of TVT versus duloxetine in women with urinary stress incontinence. International Urogynecology Journal. 2010;21(8):977-84.

413. Hutchinson AM, Milke DL, Maisey S, Johnson C, Squires JE, Teare G, et al. The Resident Assessment Instrument-Minimum Data Set 2.0 quality indicators: a systematic review. Bmc Health Services Research. 2010;10.

414. Hollingworth W, Campbell JD, Kowalski J, Ravelo A, Girod I, Briggs A, et al. Exploring the impact of changes in neurogenic urinary incontinence frequency and condition-specific quality of life on preference-based outcomes. Quality of Life Research. 2010;19(3):323-31.

415. Hernandez JG, de las Casas MO, Perez MTM, Diaz DB, Lara CM, Coello SD. Can we prevent falls in the elderly from primary care? Atencion Primaria. 2010;42(5):284-91.

416. Hasegawa J, Kuzuya M, Iguchi A. Urinary incontinence and behavioral symptoms are independent risk factors for recurrent and injurious falls, respectively, among residents in long-term care facilities. Archives of Gerontology and Geriatrics. 2010;50(1):77-81.

417. Griebling TL. Urinary Incontinence and Behavioral Symptoms are Independent Risk Factors for Recurrent and Injurious Falls, Respectively, Among Residents in Long-Term Care Facilities Editorial Comment. Journal of Urology. 2010;184(3):1054-5.

418. Goode PS, Burgio KL, Richter HE, Markland AD. Incontinence in Older Women. Jama-Journal of the American Medical Association. 2010;303(21):2172-81.

419. Golden AG, Ma QL, Nair V, Florez HJ, Roos BA. Risk for Fractures with Centrally Acting Muscle Relaxants: An Analysis of a National Medicare Advantage Claims Database. Annals of Pharmacotherapy. 2010;44(9):1369-75.

420. Ganz DA, Koretz BK, Bail JK, McCreath HE, Wenger NS, Roth CP, et al. Nurse Practitioner Comanagement for Patients in an Academic Geriatric Practice. American Journal of Managed Care. 2010;16(12):E343-E55.

421. Fry CH, Meng E, Young JS. The physiological function of lower urinary tract smooth muscle. Autonomic Neuroscience-Basic & Clinical. 2010;154(1-2):3-13.

422. Finlayson J, Morrison J, Jackson A, Mantry D, Cooper SA. Injuries, falls and accidents among adults with intellectual disabilities. Prospective cohort study. Journal of Intellectual Disability Research. 2010;54:966-80.

423. Duckett J, Basu M, Papanikolaou N. Transperineal ultrasound to assess the effect of tension-free vaginal tape position on flow rates. Ultrasound in Obstetrics & Gynecology. 2010;36(3):379-83.

424. Dubbelman Y, Groen J, Wildhagen M, Rikken B, Bosch R. The recovery of urinary continence after radical retropubic prostatectomy: a randomized trial comparing the effect of physiotherapist-guided pelvic floor muscle exercises with guidance by an instruction folder only. Bju International. 2010;106(4):515-22.

425. Cicerchia M, Ceci M, Locatelli C, Gianni W, Repetto L. Geriatric syndromes in peri-operative elderly cancer patients. Surgical Oncology-Oxford. 2010;19(3):131-9.

426. Booth JM, Lawrence M, O'Neill K, McMillan L. Exploring older peoples' experiences of nocturia: A poorly recognised urinary condition that limits participation. Disability and Rehabilitation. 2010;32(9):765-74.

427. Bloch F, Thibaud M, Dugue B, Breque C, Rigaud AS, Kemoun G. Episodes of falling among elderly people: a systematic review and meta-analysis of social and demographic pre-disposing characteristics. Clinics. 2010;65(9):895-903.

428. Wennberg AL, Molander U, Fall M, Edlund C, Peeker R, Milsom I. A Longitudinal Population-based Survey of Urinary Incontinence, Overactive Bladder, and Other Lower Urinary Tract Symptoms in Women. European Urology. 2009;55(4):783-91.

429. Wennberg AL, Molander U, Fall M, Edlund C, Peeker R, Milsom I. Lower urinary tract symptoms: lack of change in prevalence and help-seeking behaviour in two population-based surveys of women in 1991 and 2007. Bju International. 2009;104(7):954-9.

430. Wenger NS, Roth CP, Shekelle PG, Young RT, Solomon DH, Kamberg CJ, et al. A Practice-Based Intervention to Improve Primary Care for Falls, Urinary Incontinence, and Dementia. Journal of the American Geriatrics Society. 2009;57(3):547-55.

431. Wenger NS, Roth CP, Ganz D, Snow V, Minihan J, Snooks Q, et al. PRACTICE REDESIGN IMPROVES PRIMARY CARE FOR FALLS AND URINARY INCONTINENCE. Journal of General Internal Medicine. 2009;24:141-2.

432. Wang J, Kane RL, Eberly LE, Virnig BA, Chang LH. The Effects of Resident and Nursing Home Characteristics on Activities of Daily Living. Journals of Gerontology Series a-Biological Sciences and Medical Sciences. 2009;64(4):473-80.

433. Vischer UM, Bauduceau B, Bourdel-Marchasson I, Blickle JF, Constans T, Fagot-Campagna A, et al. A call to incorporate the prevention and treatment of geriatric disorders in the management of diabetes in the elderly. Diabetes & Metabolism. 2009;35(3):168-77.

434. van Hensbroek PB, van Dijk N, van Breda GF, Scheffer AC, van der Cammen TJ, Lips P, et al. The CAREFALL Triage instrument identifying risk factors for recurrent falls in elderly patients. American Journal of Emergency Medicine. 2009;27(1):23-36.

435. Temml C, Ponholzer A, Gutjahr G, Berger I, Marszalek M, Madersbacher S. Nocturia Is an Age-Independent Risk Factor for Hip-Fractures in Men. Neurourology and Urodynamics. 2009;28(8):949-52.

436. Quinn SD, Domoney C. The effects of hormones on urinary incontinence in postmenopausal women. Climacteric. 2009;12(2):106-13.

437. Oliphant SS, Wang L, Bunker CH, Lowder JL. Trends in stress urinary incontinence inpatient procedures in the United States, 1979-2004. American Journal of Obstetrics and Gynecology. 2009;200(5).

438. Min L, Yoon W, Mariano J, Wenger NS, Elliott MN, Kamberg C, et al. The Vulnerable Elders-13 Survey Predicts 5-Year Functional Decline and Mortality Outcomes in Older Ambulatory Care Patients. Journal of the American Geriatrics Society. 2009;57(11):2070-6.

439. Messinger-Rapport B, Dumas LG. Falls in the Nursing Home: A Collaborative Approach. Nursing Clinics of North America. 2009;44(2):187-+.

440. McConnell ES, Lekan D, Bunn M, Egerton E, Corazzini KN, Hendrix CD, et al. Teaching Evidence-Based Nursing Practice in Geriatric Care Settings The Geriatric Nursing Innovations Through Education Institute. Journal of Gerontological Nursing. 2009;35(4):26-35.

441. Lu FP, Lin KP, Kuo HK. Diabetes and the Risk of Multi-System Aging Phenotypes: A Systematic Review and Meta-Analysis. Plos One. 2009;4(1).

442. Lowenstein L, Pham T, Abbasy S, Kenton K, Brubaker L, Mueller ER, et al. Observations Relating to Urinary Sensation During Detrusor Overactivity. Neurourology and Urodynamics. 2009;28(6):497-500.

443. Lee PG, Cigolle C, Blaum C. The Co-Occurrence of Chronic Diseases and Geriatric Syndromes: The Health and Retirement Study. Journal of the American Geriatrics Society. 2009;57(3):511-6.

444. Lee MJ, Chang CP, Lee YH, Wu YC, Tseng HW, Tung YY, et al. Longitudinal Evaluation of an N-Ethyl-N-Nitrosourea-Created Murine Model with Normal Pressure Hydrocephalus. Plos One. 2009;4(11).

445. Lee J. Management of Acute Stroke Complication. Journal of the Korean Medical Association. 2009;52(4):365-74.

446. Kucikiene O, Lesauskaite V, Macijauskiene J, Jievaltiene G. Assessment of urinary incontinence in the elderly using the InterRAI-AC instrument. Medicina-Lithuania. 2009;45(5):365-71.

447. Irwin DE, Mungapen L, Milsom I, Kopp Z, Reeves P, Kelleher C. The economic impact of overactive bladder syndrome in six Western countries. Bju International. 2009;103(2):202-9.

448. Garcia-Fabela L, Melano-Carranza E, Aguilar-Navarro S, Garcia-Lara JMA, Gutierrez-Robledo LM, Avila-Funes JA. Hypertension as a risk factor for developing depressive symptoms among community-dwelling elders. Revista De Investigacion Clinica-Clinical and Translational Investigation. 2009;61(4):274-80.

449. Faltin DL. Epidemiology and definition of female urinary incontinence. Journal De Gynecologie Obstetrique Et Biologie De La Reproduction. 2009;38(8):S146-S52.

450. Eylgor S. Geriatric Syndromes. Turkiye Fiziksel Tip Ve Rehabilitasyon Dergisi-Turkish Journal of Physical Medicine and Rehabilitation. 2009;55:57-61.

451. Divani AA, Vazquez G, Barrett AM, Asadollahi M, Luft AR. Risk Factors Associated With Injury Attributable to Falling Among Elderly Population With History of Stroke. Stroke. 2009;40(10):3286-92.

452. Chiarelli PE, Mackenzie LA, Osmotherly PG. Urinary incontinence is associated with an increase in falls: a systematic review. Australian Journal of Physiotherapy. 2009;55(2):89-95.

453. Byles J, Millar CJ, Sibbritt DW, Chiarelli P. Living with urinary incontinence: a longitudinal study of older women. Age and Ageing. 2009;38(3):333-8.

454. Bliwise DL, Foley DJ, Vitiello MV, Ansari FP, Ancoli-Israel S, Walsh JK. Nocturia and disturbed sleep in the elderly. Sleep Medicine. 2009;10(5):540-8.

455. Bergert FW, Conrad D, Ehrenthal K, Fessler J, Gross J, Gundermann K, et al. Pharmacotherapy guidelines for the aged by family doctors for the use of family doctors. International Journal of Clinical Pharmacology and Therapeutics. 2009;47(3):141-52.

456. Araki A, Ito H. Diabetes mellitus and geriatric syndromes. Geriatrics & Gerontology International. 2009;9(2):105-14.

457. Albavera-Hernandez C, Rodriguez JM, Idrovo AJ. Safety of botulinum toxin type A among children with spasticity secondary to cerebral palsy: a systematic review of randomized clinical trials. Clinical Rehabilitation. 2009;23(5):394-407.

458. Alamgir H, Li OW, Gorman E, Fast C, Yu SC, Kidd C. Evaluation of Ceiling Lifts in Health Care Settings Patient Outcome and Perceptions. Aaohn Journal. 2009;57(9):374-80.

459. van Kerrebroeck PE, van Voskuilen AC, Heesakkers JP, Nijholt A, Siegel S, Jonas U, et al. Re: Results of sacral neuromodulation therapy for urinary voiding dysfunction: Outcomes of a prospective, worldwide clinical study - Reply. Journal of Urology. 2008;179(6):2484-.

460. Smith MD, Coppieters MW, Hodges PW. Is balance different in women with and without stress urinary incontinence? Neurourology and Urodynamics. 2008;27(1):71-8.

461. Sakakibara R, Uchiyama T, Yamanishi T, Kishi M. Dementia and lower urinary dysfunction: With a reference to anticholinergic use in elderly population. International Journal of Urology. 2008;15(9):778-88.

462. Safaz I, Alaca R, Yasar E, Tok F, Yilmaz B. Medical complications, physical function and communication skills in patients with traumatic brain injury: A single centre 5-year experience. Brain Injury. 2008;22(10):733-9.

463. Rosqvist E, Aukee P, Kallinen M, Rantanen T. Feasibility and acceptability of the pelvic floor muscle and bladder training programme. International Journal of Urological Nursing. 2008;2(3):113-8.

464. Rapp K, Lamb SE, Buchele G, Lall R, Lindemann U, Becker C. Prevention of falls in nursing homes: Subgroup analyses of a randomized fall prevention trial. Journal of the American Geriatrics Society. 2008;56(6):1092-7.

465. Rahman S, Griffin HJ, Quinn NP, Jahanshahi M. Quality of life in Parkinson's disease: The relative importance of the symptoms. Movement Disorders. 2008;23(10):1428-34.

466. Ostaszkiewicz J, O'Connell B, Millar L. Incontinence: Managed or mismanaged in hospital settings? International Journal of Nursing Practice. 2008;14(6):495-502.

467. Mock LL, Parmelee PA, Kutner N, Scott J, Johnson TM. Content validation of symptom-specific nocturia quality-of-life instrument developed in men: Issues expressed by women, as well as men. Urology. 2008;72(4):736-42.

468. Lawhorne LW, Ouslander JG, Parmelee PA, Resnick B, Calabrese B. Urinary incontinence: A neglected geriatric syndrome in nursing facilities. Journal of the American Medical Directors Association. 2008;9(1):29-35.

469. Lackner TE, Wyman JF, McCarthy TC, Monigold M, Davey C. Randomized, placebo-controlled trial of the cognitive effect, safety, and tolerability of oral extended-release oxybutynin in cognitively impaired nursing home residents with urge urinary incontinence. Journal of the American Geriatrics Society. 2008;56(5):862-70.

470. Inzitari M, Pozzi C, Ferrucci L, Chiarantini D, Rinaldi LA, Baccini M, et al. Subtle neurological abnormalities as risk factors for cognitive and functional decline, cerebrovascular events, and mortality in older community-dwelling adults. Archives of Internal Medicine. 2008;168(12):1270-6.

471. Hunter KF, Moore KN, Allen M. Lower urinary tract symptoms in older adults undergoing hip arthroplasty - A feasibility study. Journal of Wound Ostomy and Continence Nursing. 2008;35(3):334-40.

472. Hely MA, Reid WGJ, Adena MA, Halliday GA, Morris JGL. The Sydney multicenter study of Parkinson's disease: The inevitability of dementia at 20 years. Movement Disorders. 2008;23(6):837-44.

473. Harvey MA, Johnston S, Davies G. Mid-trimester serum relaxin concentrations and post-partum pelvic floor dysfunction. Acta Obstetricia Et Gynecologica Scandinavica. 2008;87(12):1315-21.

474. Groenendijk PM, Nyeholt A, Heesakkers J, van Kerrebroeck PEV, Hassouna MM, Gajewski JB, et al. Urodynamic evaluation of sacral neuromodulation for urge urinary incontinence. Bju International. 2008;101(3):325-9.

475. Fernandez HM, Callahan KE, Likourezos A, Leipzig RM. House staff member awareness of older inpatients' risks for hazards of hospitalization. Archives of Internal Medicine. 2008;168(4):390-6.

476. Farage MA, Miller KW, Berardesca E, Maibach HI. Psychosocial and societal burden of incontinence in the aged population: a review. Archives of Gynecology and Obstetrics. 2008;277(4):285-90.

477. Duckett RAJ, Grapsas P, Eaton M, Basu M. The effect of spinal anaesthesia on urethral function. International Urogynecology Journal. 2008;19(2):257-60.

478. Delbaere K, Close JCT, Menz HB, Cumming RG, Cameron ID, Sambrook PN, et al. Development and validation of fall risk screening tools for use in residential aged care facilities. Medical Journal of Australia. 2008;189(4):193-6.

479. Coll-Planas L, Denkinger MD, Nikolaus T. Relationship of urinary incontinence and late-life disability: Implications for clinical work and research in geriatrics. Zeitschrift Fur Gerontologie Und Geriatrie. 2008;41(4):283-90.

480. Cheminal R, Hotton C, Delorme E, Trackoen G, Pasquale J, Mege JL. Description and results of a prospective study on a new physiotherapy method in the management of postprostatectomy urinary incontinence. Progres En Urologie. 2008;18(5):311-7.

481. Borges P, Bretas RP, de Azevedo SF, Barbosa JMM. A profile of elderly members of community groups in Belo Horizonte, Minas Gerais State, Brazil. Cadernos De Saude Publica. 2008;24(12):2798-808.

482. Bartodziej U, Szytto K, Wtodarczyk B, Gorski J. Analysis of urodynamic study selected parameters as evaluation of lower urinary tract dysfunction following a radical hysterectomy and radiotherapy because of cervical cancer. Menopause Review-Przeglad Menopauzalny. 2008;7(6):332-7.

483. Anpalahan M, Gibson SJ. Geriatric syndromes as predictors of adverse outcomes of hospitalization. Internal Medicine Journal. 2008;38(1):16-23.

484. Wagg AR, Barron D, Kirby M, Stott D, Corlett K. A randomised partially controlled trial to assess the impact of self-help vs. structured help from a continence nurse specialist in women with undiagnosed urinary problems in primary care. International Journal of Clinical Practice. 2007;61(11):1863-73.

485. van Kerrebroeck PEV, van Voskuilen AC, Heesakkers J, Nijholt A, Siegel S, Jonas U, et al. Results of sacral neuromodulation therapy for urinary voiding dysfunction: Outcomes of a prospective, worldwide clinical study. Journal of Urology. 2007;178(5):2029-34.

486. Shakir YA, Samsioe G, Khatibi A, Nyberg P, Lidfeldt J, Agardh CD, et al. Health hazards in middle-aged women with cardiovascular disease: A case-control study of Swedish women. The Women's Health in the Lund Area (WHILA) study. Journal of Womens Health. 2007;16(3):406-14.

487. Sato M, Zuckerman IH, Stuart BC. The association between urinary incontinence and falls among the elderly living in the community: Cross-sectional study using the medicare current beneficiary survey. Journal of the American Geriatrics Society. 2007;55(4):S127-S.

488. Rubenstein LZ, Alessi CA, Josephson KR, Hoyl MT, Harker JO, Pietruszka FM. A randomized trial of a screening, case finding, and referral system for older veterans in primary care. Journal of the American Geriatrics Society. 2007;55(2):166-74.

489. Morris V, Wagg A. Lower urinary tract symptoms, incontinence and falls in elderly people: time for an intervention study. International Journal of Clinical Practice. 2007;61(2):320-3.

490. Moreira MD, Costa AR, Felipe LR, Caldas CP. The association between nursing diagnoses and the occurrence of falls observed among eldery individuals assisted in an outpatient facility. Revista Latino-Americana De Enfermagem. 2007;15(2):311-7.

491. Ljungqvist L, Peeker R, Fall M. Female urethral diverticulum: 26-year followup of a large series. Journal of Urology. 2007;177(1):219-24.

492. Liao YM, Dougherty MC, Biemer PP, Boyington AR, Liao CT, Palmer MH, et al. Prevalence of lower urinary tract symptoms among female elementary school teachers in Taipei. International Urogynecology Journal. 2007;18(10):1151-61.

493. Komesu YM, Rogers RG, Rode MA, Craig EC, Gallegos KA, Montoya AR, et al. Pelvic floor symptom changes in pessary users. American Journal of Obstetrics and Gynecology. 2007;197(6).

494. Kohta M, Minami H, Tanaka K, Kuwamura K, Kondoh T, Kohmura E. Delayed onset massive oedema and deterioration in traumatic brain injury. Journal of Clinical Neuroscience. 2007;14(2):167-70.

495. Klotz T, Bruggenjurgen B, Burkart M, Resch A. The economic costs of overactive bladder in Germany. European Urology. 2007;51(6):1654-63.

496. Kapo J, Morrison LJ, Liao S. Palliative care for the older adult. Journal of Palliative Medicine. 2007;10(1):185-209.

497. Inouye SK, Studenski S, Tinetti ME, Kuchel GA. Geriatric syndromes: Clinical, research, and policy implications of a core geriatric concept. Journal of the American Geriatrics Society. 2007;55(5):780-91.

498. Harari D, Martin FC, Buttery A, O'Neill S, Hopper A. The older persons assessment and liaison team OPAL: evaluation of comprehensive geriatric assessment in acute medical inpatients. Age and Ageing. 2007;36(6):670-5.

499. Fehrling M, Fall M, Peeker R. Maximal functional electrical stimulation as a single treatment: Is it cost-effective? Scandinavian Journal of Urology and Nephrology. 2007;41(2):132-7.

500. Eichler K, Scrabal C, Steurer J, Mann E. Preventive health risk appraisal for older people and impact on GPs' patient management: a prospective study. Family Practice. 2007;24(6):604-9.

501. DeSouza R, Shapiro A, Westney OL. Adductor brevis myositis following transobturator tape procedure: a case report and review of the literature. International Urogynecology Journal. 2007;18(7):817-20.

502. Dawson T, Lawton V, Adams E, Richmond D. Factors predictive of post-TVT voiding dysfunction. International Urogynecology Journal. 2007;18(11):1297-302.

503. Cigolle CT, Langa KM, Kabeto MU, Tian Z, Blaum CS. Geriatric conditions and disability: The health and retirement study. Annals of Internal Medicine. 2007;147(3):156-64.

504. Bottomley D, Ash D, Al-Qaisieh B, Carey B, Joseph J, St Clair S, et al. Side effects of permanent 1125 prostate seed implants in 667 patients treated in Leeds. Radiotherapy and Oncology. 2007;82(1):46-9.

505. Voaklander DC, Kelly KD, Rowe BH, Schopflocher DP, Svenson L, Yiannakoulias N, et al. Pain, medication, and injury in older farmers. American Journal of Industrial Medicine. 2006;49(5):374-82.

506. Vahakangas P, Noro A, Bjorkgren M. Provision of rehabilitation nursing in long-term care facilities. Journal of Advanced Nursing. 2006;55(1):29-35.

507. Teo JSH, Briffa NK, Devine A, Dhaliwal SS, Prince RL. Do sleep problems or urinary incontinence predict falls in elderly women? Australian Journal of Physiotherapy. 2006;52(1):19-24.

508. Swarztrauber K, Graf E, Cheng E. The quality of care delivered to Parkinson's disease patients in the US Pacific Northwest Veterans Health System. Bmc Neurology. 2006;6.

509. Schneider LS, Dagerman K, Insel PS. Efficacy and adverse effects of atypical antipsychotics for dementia: Meta-analysis of randomized, placebo-controlled trials. American Journal of Geriatric Psychiatry. 2006;14(3):191-210.

510. Reyes-Ortiz CA, Ayele H, Mulligan T, Espino DV, Berges IM, Markides KS. Higher church attendance predicts lower fear of falling in older Mexican-Americans. Aging & Mental Health. 2006;10(1):13-8.

511. Reeves P, Irwin D, Kelleher C, Milsom I, Kopp Z, Calvert N, et al. The current and future burden and cost of overactive bladder in five European countries. European Urology. 2006;50(5):1050-7.

512. Morfis L, Cordato DJ. Dementia with Lewy bodies in an elderly Greek male due to alpha-synuclein gene mutation. Journal of Clinical Neuroscience. 2006;13(9):942-4.

513. Messer KL, Herzog AR, Seng JS, Sampselle CM, Diokno AC, Raghunathan TE, et al. Evaluation of a mass mailing recruitment strategy to obtain a community sample of women for a clinical trial of an incontinence prevention intervention. International Urology and Nephrology. 2006;38(2):255-61.

514. Mattiasson A, Teleman P. Abnormal urethral motor function is common in female stress, mixed, and urge incontinence. Neurourology and Urodynamics. 2006;25(7):703-8.

515. Ishizaki T, Yoshida H, Suzuki T, Watanabe S, Niino N, Ihara K, et al. Effects of cognitive function on functional decline among community-dwelling non-disabled older Japanese. Archives of Gerontology and Geriatrics. 2006;42(1):47-58.

516. Fung CH. Computerized condition-specific templates for improving care of geriatric syndromes in a primary care setting. Journal of General Internal Medicine. 2006;21(9):989-94.

517. Durso SC. Using clinical guidelines designed for older adults with diabetes mellitus and complex health status. Jama-Journal of the American Medical Association. 2006;295(16):1935-40.

518. Dosa D, Bowers B, Gifford DR. Critical review of resident assessment protocols. Journal of the American Geriatrics Society. 2006;54(4):659-66.

519. Barber MD, Neubauer NL, Mein-Olarte V. Can we screen for pelvic organ prolapse without a physical examination in epidemiologic studies? American Journal of Obstetrics and Gynecology. 2006;195(4):942-8.

520. Avila-Funes JA, Garant MP, Aguilar-Navarro S. Relationship between determining factors for depressive symptoms and for dietary habits in older adults in Mexico. Revista Panamericana De Salud Publica-Pan American Journal of Public Health. 2006;19(5):321-30.

521. Andin U, Gustafson L, Brun A, Passant U. Clinical manifestations in neuropathologically defined subgroups of vascular dementia. International Journal of Geriatric Psychiatry. 2006;21(7):688-97.

522. Does urinary incontinence increase the risk of falls in community dwelling women? Calcified Tissue International. 2006;78:S103-S4.

523. Wenkel R, Ziemann U, Thielebein J, Prange H. Laparoscopic castration of the bitch - Presentation of new procedures for the minimally invasive ovariohysterectomy. Tieraerztliche Praxis Ausgabe Kleintiere Heimtiere. 2005;33(3):177-+.

524. Weber AM, Richter HE. Pelvic organ prolapse. Obstetrics and Gynecology. 2005;106(3):615-34.

525. Tubbs RS, Oakes WJ, Blount JP. Isolated atlantal stenosis in a patient with idiopathic growth hormone deficiency, and Klippel-Feil and Duane's syndromes. Childs Nervous System. 2005;21(5):421-4.

526. Tannenbaum C, Mayo N, Ducharme F. Older women's health priorities and perceptions of care delivery: results of the WOW health survey. Canadian Medical Association Journal. 2005;173(2):153-9.

527. Sitoh YY, Lau TC, Zochling J, Schwarz J, Chen JS, March LM, et al. Determinants of health-related quality of life in institutionalised older persons in northern Sydney. Internal Medicine Journal. 2005;35(2):131-4.

528. Ruby CM, Hanlon JT, Fillenbaum GG, Pieper CF, Branch LG, Bump RC. Medication use and control of urination among community-dwelling older adults. Journal of Aging and Health. 2005;17(5):661-74.

529. Reyes-Ortiz CA, Al Snih S, Markides KS. Falls among elderly persons in Latin America and the Caribbean and among elderly Mexican-Americans. Revista Panamericana De Salud Publica-Pan American Journal of Public Health. 2005;17(5-6):362-9.

530. Rait G, Fletcher A, Smeeth L, Brayne C, Stirling S, Nunes M, et al. Prevalence of cognitive impairment: results from the MRC trial of assessment and management of older people in the community. Age and Ageing. 2005;34(3):242-8.

531. Mecocci P, von Strauss E, Cherubini A, Ercolani S, Mariani E, Senin U, et al. Cognitive impairment is the major risk factor for development of geriatric syndromes during hospitalization: Results from the GIFA study. Dementia and Geriatric Cognitive Disorders. 2005;20(4):262-9.

532. Mamun K, Lim J. Use of physical restraints in nursing homes: Current practice in Singapore. Annals Academy of Medicine Singapore. 2005;34(2):158-62.

533. Lin TL, Ng SC, Chen YC, Hu SW, Chen GD. What affects the occurrence of nocturia more: menopause or age? Maturitas. 2005;50(2):71-7.

534. Krauss MJ, Evanoff B, Hitcho E, Ngugi KE, Dunagan WC, Fischer I, et al. A case-control study of patient, medication, and care-related risk factors for inpatient falls. Journal of General Internal Medicine. 2005;20(2):116-22.

535. Hu TW, Wagner TH. Health-related consequences of overactive bladder: an economic perspective. Bju International. 2005;96:43-5.

536. Hely MA, Morris JGL, Reid WGJ, Trafficante R. Sydney multicenter study of Parkinson's disease: non-L-dopa-responsive problems dominate at 15 years. Movement Disorders. 2005;20(2):190-9.

537. Heath JM, Brown M, Kobylarz FA, Castano S. The prevalence of undiagnosed geriatric health conditions among adult protective service clients. Gerontologist. 2005;45(6):820-3.

538. Haab F, Castro-Diaz D. Persistence with antimuscarinic therapy in patients with overactive bladder. International Journal of Clinical Practice. 2005;59(8):931-7.

539. Glazener CMA, Herbison GP, MacArthur C, Grant A, Wilson PD. Randomised controlled trial of conservative management of postnatal urinary and faecal incontinence: six year follow up. British Medical Journal. 2005;330(7487):337-9.

540. Ghei M, Malone-Lee J. Using the circumstances of symptom experience to assess the severity of urgency in the overactive bladder. Journal of Urology. 2005;174(3):972-6.

541. Darkow T, Fontes CL, Williamson TE. Costs associated with the management of overactive bladder and related comorbidities. Pharmacotherapy. 2005;25(4):511-9.

542. Brunton S, Kuritzky L. Recent developments in the management of overactive bladder: focus on the efficacy and tolerability of once daily solifenacin succinate 5 mg. Current Medical Research and Opinion. 2005;21(1):71-80.

543. Becker C, Loy S, Sander S, Nikolaus T, Rissmann U, Kron M. An algorithm to screen long-term care residents at risk for accidental falls. Aging Clinical and Experimental Research. 2005;17(3):186-92.

544. Balash Y, Peretz C, Leibovich G, Herman T, Hausdorff JM, Giladi N. Falls in outpatients with Parkinson's disease - Frequency, impact and identifying factors. Journal of Neurology. 2005;252(11):1310-5.

545. Asplund R, Johansson S, Henriksson S, Isacsson G. Nocturia, depression and antidepressant medication. Bju International. 2005;95(6):820-3.

546. Asplund R. Nocturia in relation to sleep, health, and medical treatment in the elderly. Bju International. 2005;96:15-21.

547. Warnke A, Meyer G, Bender R, Muhlhauser I. Predictors of adherence to the use of hip protectors in nursing home residents. Journal of the American Geriatrics Society. 2004;52(3):340-5.

548. Sandholzer H, Hellenbrand W, Renteln-Kruse W, Van Weel C, Walker P. STEP - Standardized assessment of elderly people in primary care. Deutsche Medizinische Wochenschrift. 2004;129:S183-S226.

549. Oliver D, Daly F, Martin FC, McMurdo MET. Risk factors and risk assessment tools for falls in hospital in-patients: a systematic review. Age and Ageing. 2004;33(2):122-30.

550. Matzel KE, Kamm MA, Stosser M, Baeten C, Christiansen J, Madoff R, et al. Sacral spinal nerve stimulation for faecal incontinence: multicentre study. Lancet. 2004;363(9417):1270-6.

551. Marin PP, Gac H, Hoyl T, Carrasco M, Duery P, Cabezas M, et al. A comparative study of institutionalized nonagenarian and younger elderly women. Revista Medica De Chile. 2004;132(1):33-9.

552. Marin PP, Carrasco M, Cabezas M, Gac H, Hoyl T, Duery P, et al. Biomedical impact of traveling for Chilean elderly. Revista Medica De Chile. 2004;132(5):573-8.

553. Kuo HK, Lipsitz LA. Cerebral white matter changes and geriatric syndromes: Is there a link? Journals of Gerontology Series a-Biological Sciences and Medical Sciences. 2004;59(8):818-26.

554. Knight GE, Burnstock G. The effect of pregnancy and the oestrus cycle on purinergic and cholinergic responses of the rat urinary bladder. Neuropharmacology. 2004;46(7):1049-56.

555. Fall M. Do objective urodynamic or clinical findings determine impact of urinary incontinence or its treatment on quality of life? Editorial comment. Urology. 2004;63(1):71-2.

556. Edlund C, Dijkema HE, Hassouna MM, Van Kerrebroeck PEV, Peeker R, Van den Hombergh U, et al. Sacral nerve stimulation for refractory urge symptoms in elderly patients. Scandinavian Journal of Urology and Nephrology. 2004;38(2):131-5.

557. Chen CCH, Kenefick AL, Tang ST, McCorkle R. Utilization of comprehensive geriatric assessment in cancer patients. Critical Reviews in Oncology Hematology. 2004;49(1):53-67.

558. Cardozo L, Lisec M, Millard R, Trip OV, Kuzmin I, Drogendijk TE, et al. Randomized, double-blind placebo controlled trial of the once daily antimuscarinic agent solifenacin succinate in patients with overactive bladder. Journal of Urology. 2004;172(5):1919-24.

559. Asplund R. Nocturia, nocturnal polyuria, and sleep quality in the elderly. Journal of Psychosomatic Research. 2004;56(5):517-25.

560. Wilson TS, Lemack GE, Zimmern PE. Management of intrinsic sphincteric deficiency in women. Journal of Urology. 2003;169(5):1662-9.

561. Wennberg AL, Edlund C, Fall M, Peeker R. Stamey's abdominovaginal needle colposuspension for the correction of female genuine stress urinary incontinence - Long-term results. Scandinavian Journal of Urology and Nephrology. 2003;37(5):419-23.

562. Vanderzeypen F, Bier JC, Genevrois C, Mendlewicz J, Lotstra F. Frontal Dementia or Dementia Praecox ? The case report of a psychotic disorder with a severe decline. Encephale-Revue De Psychiatrie Clinique Biologique Et Therapeutique. 2003;29(2):172-80.

563. Specht-Leible N, Bender M, Oster P. Causes for in-hospital treatment of nursing home residents. Zeitschrift Fur Gerontologie Und Geriatrie. 2003;36(4):274-9.

564. Schnelle JF, Kapur K, Alessi C, Osterweil D, Beck JG, Al-Samarrai NR, et al. Does an exercise and incontinence intervention save healthcare costs in a nursing home population? Journal of the American Geriatrics Society. 2003;51(2):161-8.

565. Schick E, Tessier J, Bertrand PE, Dupont C, Jolivet-Tremblay M. Observations on the function of the female urethra: I: Relation between maximum urethral closure pressure at rest and urethral hypermobility. Neurourology and Urodynamics. 2003;22(7):643-7.

566. Reuben DB, Roth C, Kamberg C, Wenger NS. Restructuring primary care practices to manage geriatric syndromes: The ACOVE-2 intervention. Journal of the American Geriatrics Society. 2003;51(12):1787-93.

567. Pils K, Neumann F, Meisner W, Schano W, Vavrovsky G, Van der Cammen TJM. Predictors of falls in elderly people during rehabilitation after hip fracture - who is at risk of a second one? Zeitschrift Fur Gerontologie Und Geriatrie. 2003;36(1):16-22.

568. Moty C, Barberger-Gateau P, De Sarasqueta AM, Teare GF, Henrard JC. Risk adjustment of quality indicators in French long term care facilities for elderly people. A preliminary study. Revue D Epidemiologie Et De Sante Publique. 2003;51(3):327-38.

569. Lindesay J, Matthews R, Jagger C. Factors associated with antipsychotic drug use in residential care: changes between 1990 and 1997. International Journal of Geriatric Psychiatry. 2003;18(6):511-9.

570. Kron M, Loy S, Sturm E, Nikolaus T, Becker C. Risk indicators for falls in institutionalized frail elderly. American Journal of Epidemiology. 2003;158(7):645-53.

571. Dehkharghani S, Bible J, Chen JG, Feldman SR, Fleischer AB. The economic burden of skin disease in the United States. Journal of the American Academy of Dermatology. 2003;48(4):592-9.

572. de Rekeneire N, Visser M, Peila R, Nevitt MC, Cauley JA, Tylavsky FA, et al. Is a fall just a fall: Correlates of falling in healthy older persons. The health, aging and body composition study. Journal of the American Geriatrics Society. 2003;51(6):841-6.

573. Broadhurst C, Wilson KCM, Kinirons MT, Wagg A, Dhesi JK. Clinical pharmacology of old age syndromes. British Journal of Clinical Pharmacology. 2003;56(3):261-72.

574. Behrens PMI, Diaz TV, Vasquez VC, Donoso SA. Dementia caused by vitamin B12 deficiency. Report of one case. Revista Medica De Chile. 2003;131(8):915-9.

575. Alessi CA, Josephson KR, Harker JO, Pietruszka FM, Hoyl MT, Rubenstein LZ. The yield, reliability, and validity of a postal survey for screening community-dwelling older people. Journal of the American Geriatrics Society. 2003;51(2):194-202.

576. Wein A, Lose GR, Fonda D. Nocturia in men, women and the elderly: a practical approach. Bju International. 2002;90:28-31.

577. Wagner TH, Hu TW, Bentkover J, LeBlanc K, Stewart W, Corey R, et al. Health-related consequences of overactive bladder. American Journal of Managed Care. 2002;8(19):S598-S607.

578. van Schoor NM, Deville WL, Bouter LM, Lips P. Acceptance and compliance with external hip protectors: A systematic review of the literature. Osteoporosis International. 2002;13(12):917-24.

579. Thakar R, Ayers S, Clarkson P, Stanton S, Manyonda I. Outcomes after total versus subtotal abdominal hysterectomy. New England Journal of Medicine. 2002;347(17):1318-25.

580. Stoddart H, Whitley E, Harvey I, Sharp D. What determines the use of home care services by elderly people? Health & Social Care in the Community. 2002;10(5):348-60.

581. Scheepens WA, de Bie RA, Weil EHJ, van Kerrebroeck PEV. Unilateral versus bilateral sacral neuromodulation in patients with chronic voiding dysfunction. Journal of Urology. 2002;168(5):2046-50.

582. Saltvedt I, Mo ESO, Fayers P, Kaasa S, Sletvold O. Reduced mortality in treating acutely sick, frail older patients in a geriatric evaluation and management unit. A prospective randomized trial. Journal of the American Geriatrics Society. 2002;50(5):792-8.

583. Peeker R, Edlund C, Wennberg AL, Fall M. The treatment of sphincter incontinence with periurethral silicone implants (Macroplastique). Scandinavian Journal of Urology and Nephrology. 2002;36(3):194-8.

584. Kelleher CJ. Economic and social impact of OAB. European Urology Supplements. 2002;1(4):11-6.

585. Girman CJ, Chandler JM, Zimmerman SI, Martin AR, Hawkes W, Hebel JR, et al. Prediction of fracture in nursing home residents. Journal of the American Geriatrics Society. 2002;50(8):1341-7.

586. Coppola L, Caserta F, Grassia A, Mastrolorenzo L, Altrui L, Tondi G, et al. Urinary incontinence in the elderly: relation to cognitive and motor function. Archives of Gerontology and Geriatrics. 2002;35(1):27-34.

587. Brown JS. Epidemiology and changing demographics of overactive bladder: A focus on the postmenopausal woman. Geriatrics. 2002;57:6-12.

588. Ali-El-Dein B, Gomha M, Ghoneim MA. Critical evaluation of the problem of chronic urinary retention after orthotopic bladder substitution in women. Journal of Urology. 2002;168(2):587-92.

589. Ahlberg J, Edlund C, Wikkelso C, Rosengren L, Fall M. Neurological signs are common in patients with urodynamically verified "idiopathic" bladder overactivity. Neurourology and Urodynamics. 2002;21(1):65-70.

590. Whitman GT. Patients with urinary incontinence and falls. Journal of the American Geriatrics Society. 2001;49(3):336-7.

591. Tromp AM, Pluijm SMF, Smit JH, Deeg DJH, Bouter LM, Lips P. Fall-risk screening test: A prospective study on predictors for falls in community-dwelling elderly. Journal of Clinical Epidemiology. 2001;54(8):837-44.

592. Sze KH, Wong E, Leung HY, Woo J. Falls among Chinese stroke patients during rehabilitation. Archives of Physical Medicine and Rehabilitation. 2001;82(9):1219-25.

593. Simons AM, Dowell CJ, Bryant CM, Prashar S, Moore KH. Use of the Dowell Bryant Incontinence Cost Index as a post-treatment outcome measure after non-surgical therapy. Neurourology and Urodynamics. 2001;20(1):85-93.

594. Radley SC, Chapple CR, Bryan NP, Clarke DE, Craig DA. Effect of methoxamine on maximum urethral pressure in women with genuine stress incontinence: A placebo-controlled, double-blind crossover study. Neurourology and Urodynamics. 2001;20(1):43-52.

595. Kirkham APS, Shah NC, Knight SL, Shah PJR, Craggs MD. The acute effects of continuous and conditional neuromodulation on the bladder in spinal cord injury. Spinal Cord. 2001;39(8):420-8.

596. Johnson TM, Ouslander JG. The shifting impact of UI. Journal of the American Geriatrics Society. 2001;49(7):998-9.

597. Janknegt RA, Hassouna MM, Siegel SW, Schmidt RA, Gajewski JB, Rivas DA, et al. Long-term effectiveness of sacral nerve stimulation for refractory urge incontinence. European Urology. 2001;39(1):101-6.

598. Goetz SM, Stuck AE, Hirschi A, Gillmann G, Dapp U, Nikolaus T, et al. Test-retest reliability of a newly developed German language instrument for multidimensional geriatric assessment. Zeitschrift Fur Gerontologie Und Geriatrie. 2001;34(3):196-206.

599. Fader M, Pettersson L, Dean G, Brooks R, Cottenden AM, Malone-Lee J. Sheaths for urinary incontinence: a randomized crossover trial. Bju International. 2001;88(4):367-72.

600. Edlund C, Peeker R, Fall M. Lidocaine cystometry in the diagnosis of bladder overactivity. Neurourology and Urodynamics. 2001;20(2):147-55.

601. Edlund C, Peeker R, Fall M. Clam ileocystoplasty: Successful treatment of severe bladder overactivity. Scandinavian Journal of Urology and Nephrology. 2001;35(3):190-5.

602. Dhar HL. Gender, aging, health and society. Journal of Association of Physicians of India. 2001;49:1012-20.

603. Sloss EM, Solomon DH, Shekelle PG, Young RT, Saliba D, MacLean CH, et al. Selecting target conditions for quality of care improvement in vulnerable older adults. Journal of the American Geriatrics Society. 2000;48(4):363-9.

604. Nair B, O'Dea I, Lim L, Thakkinstian A. Prevalence of geriatric 'syndromes' in a tertiary hospital. Australasian Journal on Ageing. 2000;19(2):81-4.

605. Miller KE, Zylstra RG, Standridge JB. The geriatric patient: A systematic approach to maintaining health. American Family Physician. 2000;61(4):1089-104.

606. McGann PE. Comorbidity in heart failure in the elderly. Clinics in Geriatric Medicine. 2000;16(3):631-+.

607. Johnson JC, Jayadevappa R, Baccash PD, Taylor L. Nonspecific presentation of pneumonia in hospitalized older people: Age effect or dementia? Journal of the American Geriatrics Society. 2000;48(10):1316-20.

608. Goetz SM, Stuck AE, Hirschi A, Gillmann G, Dapp U, Minder CE, et al. A new multidimensional assessment instrument in German for prevention in older persons: Comparison of the self-administered with the interviewer-administered version. Sozial-Und Praventivmedizin. 2000;45(3):134-46.

609. FitzGerald MP, Mollenhauer J, Hale DS, Benson JT, Brubaker L. Urethral collagen morphologic characteristics among women with genuine stress incontinence. American Journal of Obstetrics and Gynecology. 2000;182(6):1565-72.

610. Edlund C, Hellstrom M, Peeker R, Fall M. First Scandinavian experience of electrical sacral nerve stimulation in the treatment of the overactive bladder. Scandinavian Journal of Urology and Nephrology. 2000;34(6):366-76.

611. Clancy CM, Bierman AS. Quality and outcomes of care for older women with chronic disease. Womens Health Issues. 2000;10(4):178-90.

612. Brown JS, Vittinghoff E, Wyman JF, Stone KL, Nevitt MC, Ensrud KE, et al. Urinary incontinence: Does it increase risk for falls and fractures? Journal of the American Geriatrics Society. 2000;48(7):721-5.

613. Brown JS, McGhan WF, Chokroverty S. Comorbidities associated with overactive bladder. American Journal of Managed Care. 2000;6(11):S574-S9.

614. Bowles J, Brooks T, Hayes-Reams P, Butts T, Myers H, Allen W, et al. Frailty family, and church support among urban African American elderly. Journal of Health Care for the Poor and Underserved. 2000;11(1):87-99.

615. Bo K, Berghmans LCM. Nonpharmacologic treatments for overactive bladder - Pelvic floor exercises. Urology. 2000;55(5A):7-11.

616. Sugerman HJ, Felton WL, Sismanis A, Kellum JM, DeMaria EJ, Sugerman EL. Gastric surgery for pseudotumor cerebri associated with severe obesity. Annals of Surgery. 1999;229(5):634-42.

617. Saint S, Lipsky BA, Baker PD, McDonald LL, Ossenkop K. Urinary catheters: What type do men and their nurses prefer? Journal of the American Geriatrics Society. 1999;47(12):1453-7.

618. Reuben DB, Frank JC, Hirsch SH, McGuigan KA, Maly RC. A randomized clinical trial of outpatient comprehensive geriatric assessment coupled with an intervention to increase adherence to recommendations. Journal of the American Geriatrics Society. 1999;47(3):269-76.

619. Perlmutter AP, Vallancien G. Thick loop transurethral resection of the prostate. European Urology. 1999;35(2):161-5.

620. Pakiam ASI, Bergeron C, Lang AE. Diffuse Lewy body disease presenting as multiple system atrophy. Canadian Journal of Neurological Sciences. 1999;26(2):127-31.

621. Gregory SP, Holt PE, Parkinson TJ, Wathes CM. Vaginal position and length in the bitch: relationship to spaying and urinary incontinence. Journal of Small Animal Practice. 1999;40(4):180-4.

622. Geirsson G, Lindstrom S, Fall M. The bladder cooling reflex and the use of cooling as stimulus to the lower urinary tract. Journal of Urology. 1999;162(6):1890-6.

623. Coleman EA, Grothaus LC, Sandhu N, Wagner EH. Chronic care clinics: A randomized controlled trial of a new model of primary care for frail older adults. Journal of the American Geriatrics Society. 1999;47(7):775-83.

624. Carod-Artal FJ. Measurement of the quality of life in stroke survivors. Revista De Neurologia. 1999;29(5):447-56.

625. Caplan GA, Ward JA, Brennan NJ, Coconis J, Board N, Brown A. Hospital in the home: a randomised controlled trial. Medical Journal of Australia. 1999;170(4):156-60.

626. Tromp AM, Smit JH, Deeg DJH, Bouter LM, Lips P. Predictors for falls and fractures in the longitudinal aging study Amsterdam. Journal of Bone and Mineral Research. 1998;13(12):1932-9.

627. Stude DE, Bergmann TF, Finer BA. A conservative approach for a patient with traumatically induced urinary incontinence. Journal of Manipulative and Physiological Therapeutics. 1998;21(5):363-7.

628. Spennacchio M, Buonaguidi A, Bertola E, Penotti M, Vignali M. Vaginal surgery for genital prolapse associated with stress urinary incontinence: A retrospective study. Journal of Gynecologic Surgery. 1998;14(4):175-9.

629. Sherman SE, Reuben D. Measures of functional status in community-dwelling elders. Journal of General Internal Medicine. 1998;13(12):817-23.

630. Rentzhog L, Stanton SL, Cardozo L, Nelson E, Fall M, Abrams P. Efficacy and safety of tolterodine in patients with detrusor instability: a dose-ranging study. British Journal of Urology. 1998;81(1):42-8.

631. Madersbacher H, Awad S, Fall M, Janknegt RA, Stohrer M, Weisner B. Urge incontinence in the elderly - supraspinal reflex incontinence. World Journal of Urology. 1998;16:S35-S43.

632. Kobelt G, Jonsson L, Mattiasson A. Cost-effectiveness of new treatments for overactive bladder: The example of tolterodine, a new muscarinic agent: A Markov model. Neurourology and Urodynamics. 1998;17(6):599-611.

633. Fall M. Advantages and pitfalls of functional electrical stimulation. Acta Obstetricia Et Gynecologica Scandinavica. 1998;77:16-21.

634. Cho CY, Alessi CA, Cho M, Aronow HU, Stuck AE, Rubenstein LZ, et al. The association between chronic illness and functional change among participants in a comprehensive geriatric assessment program. Journal of the American Geriatrics Society. 1998;46(6):677-82.

635. Wilson PD, George M, Imrie JJ. Vaginal electrostimulation for the treatment of genuine stress incontinence. Australian & New Zealand Journal of Obstetrics & Gynaecology. 1997;37(4):446-9.

636. Tutuarima JA, vanderMeulen JHP, deHaan RJ, vanStraten A, Limburg M. Risk factors for falls of hospitalized stroke patients. Stroke. 1997;28(2):297-301.

637. Sheriff MKM, Foley S, McFarlane J, NauthMisir R, Shah PJR. Endoscopic correction of intractable stress incontinence with silicons micro-implants. European Urology. 1997;32(3):284-8.

638. Shah PN, Maly RC, Frank JC, Hirsch SH, Reuben DB. Managing geriatric syndromes: What geriatric assessment teams recommend, what primary care physicians implement, what patients adhere to. Journal of the American Geriatrics Society. 1997;45(4):413-9.

639. Rubio JIM, Zunzunegui MV, Beland F. Prevalence and factors associated to falls among non-institutionalized elderly people. Medicina Clinica. 1997;108(4):128-32.

640. Nyberg L, Gustafson Y. Fall prediction index for patients in stroke rehabilitation. Stroke. 1997;28(4):716-21.

641. Moore KH, Foote A, Siva S, King J, Burton G. The use of the bladder neck support prosthesis in combined genuine stress incontinence and detrusor instability. Australian & New Zealand Journal of Obstetrics & Gynaecology. 1997;37(4):440-5.

642. Maly RC, Hirsch SH, Reuben DB. The performance of simple instruments in detecting geriatric conditions and selecting community-dwelling older people for geriatric assessment. Age and Ageing. 1997;26(3):223-31.

643. Geirsson G, Fall M. Maximal functional electrical stimulation in routine practice. Neurourology and Urodynamics. 1997;16(6):559-65.

644. Donahue JL, Lowenthal DT. Nocturnal polyuria in the elderly person. American Journal of the Medical Sciences. 1997;314(4):232-8.

645. Chapron C, Dubuisson JB, Ansquer Y, CapellaAllouc S. Hysterectomy with adnexectomy - Can operative laparoscopy offer advantages? Journal of Reproductive Medicine. 1997;42(4):201-6.

646. Bump RC, Coates KW, Cundiff GW, Harris RL, Weidner AC. Diagnosing intrinsic sphincteric deficiency: Comparing urethral closure pressure, urethral axis, and Valsalva leak point pressures. American Journal of Obstetrics and Gynecology. 1997;177(2):303-10.

647. Brandeis GH, Baumann MM, Hossain M, Morris JN, Resnick NM. The prevalence of potentially remediable urinary incontinence in frail older people: A study using the minimum data set. Journal of the American Geriatrics Society. 1997;45(2):179-84.

648. Yusuf SW, Booth SA, Mishra RM. Falls and urinary incontinence in a 66-year-old woman. Lancet. 1996;347(9017):1738-.

649. Trsinar B, Kralj B. Maximal electrical stimulation in children with unstable bladder and nocturnal enuresis and/or daytime incontinence: A controlled study. Neurourology and Urodynamics. 1996;15(2):133-42.

650. Reuben DB, Maly RC, Hirsch SH, Frank JC, Oakes AM, Siu AL, et al. Physician implementation of and patient adherence to recommendations from comprehensive geriatric assessment. American Journal of Medicine. 1996;100(4):444-51.

651. Pressman MR, Figueroa WG, KendrickMohamed J, Greenspon LW, Peterson DD. Nocturia - A rarely recognized symptom of sleep apnea and other occult sleep disorders. Archives of Internal Medicine. 1996;156(5):545-50.

652. Mills R, Persad R, Ashken MH. Long-term follow-up results with the Stamey operation for stress incontinence of urine. British Journal of Urology. 1996;77(1):86-8.

653. Marin P, Valenzuela E, Saito NK, Castro S, Hoyl T. Pilot experience with an ambulatory geriatric assessment instrument. Revista Medica De Chile. 1996;124(6):701-6.

654. Luukinen H, Koski K, Kivela SL, Laippala P. Social status, life changes, housing conditions, health, functional abilities and life-style as risk factors for recurrent falls among the home-dwelling elderly. Public Health. 1996;110(2):115-8.

655. Hahn I, Milsom I, Ohlsson BL, Ekelund P, Uhlemann C, Fall M. Comparative assessment of pelvic floor function using vaginal cones, vaginal digital palpation and vaginal pressure measurements. Gynecologic and Obstetric Investigation. 1996;41(4):269-74.

656. Chancellor MB, Shenot PJ, Hong RD, Watanabe T, Rivas DA. Fascial sling correction of kinked efferent limb in patients with continent diversion and catheterization difficulty. Journal of Urology. 1996;156(1):162-3.

657. Voge VM, Hastings JD, Drew WE. CONVULSIVE SYNCOPE IN THE AVIATION ENVIRONMENT. Aviation Space and Environmental Medicine. 1995;66(12):1198-204.

658. Tinetti ME, Inouye SK, Gill TM, Doucette JT. SHARED RISK-FACTORS FOR FALLS, INCONTINENCE, AND FUNCTIONAL DEPENDENCE - UNIFYING THE APPROACH TO GERIATRIC SYNDROMES. Jama-Journal of the American Medical Association. 1995;273(17):1348-53.

659. Ruscoe M, Nowers M, Hastie I. A POSSIBLE CASE OF ELDERLY ABUSE. Practitioner. 1995;239(1551):355-&.

660. Lipsitz LA. The teaching nursing home: Past accomplishments and future directions. Generations-Journal of the American Society on Aging. 1995;19(4):47-51.

661. Fall M. VAGINAL WALL BIPEDICLED FLAP AND OTHER TECHNIQUES IN COMPLICATED URETHRAL DIVERTICULUM AND URETHROVAGINAL FISTULA. Journal of the American College of Surgeons. 1995;180(2):150-6.

662. Chin YK, Stanton SL. A FOLLOW-UP OF SILASTIC SLING FOR GENUINE STRESS-INCONTINENCE. British Journal of Obstetrics and Gynaecology. 1995;102(2):143-7.

663. Yasumura S, Haga H, Nagai H, Suzuki T, Amano H, Shibata H. RATE OF FALLS AND THE CORRELATES AMONG ELDERLY PEOPLE LIVING IN AN URBAN-COMMUNITY IN JAPAN. Age and Ageing. 1994;23(4):323-7.

664. Tayal SC, Bansal SK, Chadha DK. HYPOPITUITARISM - A DIFFICULT DIAGNOSIS IN ELDERLY PEOPLE BUT WORTH A SEARCH. Age and Ageing. 1994;23(4):320-2.

665. Sutton JC, Standen PJ, Wallace WA. PATIENT ACCIDENTS IN-HOSPITAL - INCIDENCE, DOCUMENTATION AND SIGNIFICANCE. British Journal of Clinical Practice. 1994;48(2):63-6.

666. Santora TA, Schinco MA, Trooskin SZ. MANAGEMENT OF TRAUMA IN THE ELDERLY PATIENT. Surgical Clinics of North America. 1994;74(1):163-86.

667. Ory MG, Cox DM. FORGING AHEAD - LINKING HEALTH AND BEHAVIOR TO IMPROVE QUALITY-OF-LIFE IN OLDER-PEOPLE. Social Indicators Research. 1994;33(1-3):89-120.

668. Nikolaus T, Barlet J, Burkhard K, Lamar N, Oster P, Schlierf G. FUNCTIONAL ASSESSMENT OF ELDERLY PATIENTS IN A GENERAL-PRACTICE. Zeitschrift Fur Gerontologie. 1994;27(6):437-41.

669. Mayo NE, Gloutney L, Levy AR. RANDOMIZED TRIAL OF IDENTIFICATION BRACELETS TO PREVENT FALLS AMONG PATIENTS IN A REHABILITATION HOSPITAL. Archives of Physical Medicine and Rehabilitation. 1994;75(12):1302-8.

670. Kutner NG, Schechtman KB, Ory MG, Baker DI, Miller JP, Province MA, et al. OLDER ADULTS PERCEPTIONS OF THEIR HEALTH AND FUNCTIONING IN RELATION TO SLEEP DISTURBANCE, FALLING, AND URINARY-INCONTINENCE. Journal of the American Geriatrics Society. 1994;42(7):757-62.

671. Fowler CJ, Beck RO, Gerrard S, Betts CD, Fowler CG. INTRAVESICAL CAPSAICIN FOR TREATMENT OF DETRUSOR HYPERREFLEXIA. Journal of Neurology Neurosurgery and Psychiatry. 1994;57(2):169-73.

672. Schnizer W. THERAPEUTIC MUSCLE TRAINING. Wiener Klinische Wochenschrift. 1993;105(8):232-8.

673. Ramsay IN, Hilton P, Cox TF. TIME-SERIES ANALYSIS OF URETHRAL ELECTRICAL CONDUCTANCE MEASUREMENTS IN THE ASSESSMENT OF UNSTABLE URETHRAL PRESSURE - RESULTS IN NORMAL-PATIENTS AND IN THOSE WITH GENUINE STRESS-INCONTINENCE. Neurourology and Urodynamics. 1993;12(1):23-31.

674. Henschke PJ. INFECTIONS IN THE ELDERLY. Medical Journal of Australia. 1993;158(12):830-&.

675. Hamdy RC, Hudgins LB, Compton R. MANAGEMENT OF HYPERTENSION IN OLDER PATIENTS. Southern Medical Journal. 1993;86(10):S1-S6.

676. Hahn I, Milsom I, Fall M, Ekelund P. LONG-TERM RESULTS OF PELVIC FLOOR TRAINING IN FEMALE STRESS URINARY-INCONTINENCE. British Journal of Urology. 1993;72(4):421-7.

677. Geirsson G, Fall M, Lindstrom S. SUBTYPES OF OVERACTIVE BLADDER IN OLD-AGE. Age and Ageing. 1993;22(2):125-31.

678. Geirsson G, Fall M, Lindstrom S. THE ICE-WATER TEST - A SIMPLE AND VALUABLE SUPPLEMENT TO ROUTINE CYSTOMETRY. British Journal of Urology. 1993;71(6):681-5.

679. Fox RA. TREATMENT RECOMMENDATIONS FOR RESPIRATORY-TRACT INFECTIONS ASSOCIATED WITH AGING. Drugs & Aging. 1993;3(1):40-8.

680. Rickwood AMK. MANAGEMENT OF THE INCONTINENT CHILD IN GENERAL-PRACTICE - THE PEDIATRIC UROLOGISTS VIEWPOINT. Scandinavian Journal of Urology and Nephrology. 1992:117-25.

681. Odonnell P, Hanish HM. TELEMETRIC ELECTROMYOGRAPHIC MONITORING IN ELDERLY INCONTINENT MEN. Neurourology and Urodynamics. 1992;11(2):115-21.

682. Freistuhler M, Passenberg P, Burger M. SHY-DRAGER SYNDROME. Deutsche Medizinische Wochenschrift. 1992;117(30):1146-8.

683. Beckingham IJ, Wemyssholden G, Lawrence WT. LONG-TERM FOLLOW-UP OF WOMEN TREATED WITH PERURETHRAL TEFLON INJECTIONS FOR STRESS-INCONTINENCE. British Journal of Urology. 1992;69(6):580-3.

684. Versi E, Cardozo L, Anand D, Cooper D. SYMPTOMS ANALYSIS FOR THE DIAGNOSIS OF GENUINE STRESS-INCONTINENCE. British Journal of Obstetrics and Gynaecology. 1991;98(8):815-9.

685. Schreiter F. OPERATIVE THERAPY OF INCONTINENCE IN THE MAN. Urologe-Ausgabe A. 1991;30(4):223-30.

686. Lagrojanssen TLM, Debruyne FMJ, Smits AJA, Vanweel C. CONTROLLED TRIAL OF PELVIC FLOOR EXERCISES IN THE TREATMENT OF URINARY STRESS-INCONTINENCE IN GENERAL-PRACTICE. British Journal of General Practice. 1991;41(352):445-9.

687. Hilton P, Mayne CJ. THE STAMEY ENDOSCOPIC BLADDER NECK SUSPENSION - A CLINICAL AND URODYNAMIC INVESTIGATION, INCLUDING ACTUARIAL FOLLOW-UP OVER 4 YEARS. British Journal of Obstetrics and Gynaecology. 1991;98(11):1141-9.

688. Hahn I, Sommar S, Fall M. A COMPARATIVE-STUDY OF PELVIC FLOOR TRAINING AND ELECTRICAL-STIMULATION FOR THE TREATMENT OF GENUINE FEMALE STRESS URINARY-INCONTINENCE. Neurourology and Urodynamics. 1991;10(6):545-54.

689. Hahn I, Naucler J, Sommar S, Fall M. URODYNAMIC ASSESSMENT OF PELVIC FLOOR TRAINING. World Journal of Urology. 1991;9(3):162-6.

690. Hahn I, Fall M. OBJECTIVE QUANTIFICATION OF STRESS URINARY-INCONTINENCE - A SHORT, REPRODUCIBLE, PROVOCATIVE PAD-TEST. Neurourology and Urodynamics. 1991;10(5):475-81.

691. Fall M, Lindstrom S. ELECTRICAL-STIMULATION - A PHYSIOLOGICAL APPROACH TO THE TREATMENT OF URINARY-INCONTINENCE. Urologic Clinics of North America. 1991;18(2):393-407.

692. Ebrahim S, Patel N, Coats M, Greig C, Gilley J, Bangham C, et al. PREVALENCE AND SEVERITY OF MORBIDITY AMONG GUJARATI ASIAN ELDERS - A CONTROLLED COMPARISON. Family Practice. 1991;8(1):57-62.

693. Ohlsson B, Fall M. ADVANTAGES OF USING PULSES OF SHORT DURATION IN ELECTRICAL-STIMULATION FOR THE TREATMENT OF URINARY-INCONTINENCE. Artificial Organs. 1987;11(5):428-.

694. Fall M, Erlandson BE, Pettersson S. EVALUATION OF HISTORY AND SIMPLE SUPINE CYSTOMETRY AS A PREOPERATIVE TEST IN STRESS URINARY-INCONTINENCE. Acta Obstetricia Et Gynecologica Scandinavica. 1984;63(3):241-4.

695. Fall M. DOES ELECTROSTIMULATION CURE URINARY-INCONTINENCE. Journal of Urology. 1984;131(4):664-7.

696. Yalcintas E, Demirci H, Karlibel IA, Turkoglu AR, Aksoy MK, Coban S. Geriatric giants in women over 65 years living in a rural area in Turkey. Journal of Women & Aging.

697. Lehti TE, Ohman H, Knuutila M, Kautiainen H, Karppinen H, Tilvis R, et al. Symptom Burden Is Associated with Psychological Wellbeing and Mortality in Older Adults. Journal of Nutrition Health & Aging.

698. Konishi S, Hatakeyama S, Imai A, Kumagai M, Okita K, Togashi K, et al. Overactive bladder and sleep disturbance have a significant effect on indoor falls: Results from the community health survey in Japan. Luts-Lower Urinary Tract Symptoms.

699. Kachru N, Holmes HM, Johnson ML, Chen H, Aparasu RR. Comparative risk of adverse outcomes associated with nonselective and selective antimuscarinic medications in older adults with dementia and overactive bladder. International Journal of Geriatric Psychiatry.

700. Hendriks M, Bartolo S, Giraudet G, Cosson M, Chazard E. Change over time in the surgical management of pelvic organ prolapse between 2008 and 2014 in France: patient profiles, surgical approaches, and outcomes. International Urogynecology Journal.

701. El-Hamamsy D, Tincello DG. Recurrent stress urinary incontinence surgery in the United Kingdom: an analysis of the British Society of Urogynaecology database (2007-2015). International Urogynecology Journal.

702. Corcoles-Jimenez MP, Candel-Parra E, del Egido-Fernandez MA, Villada-Munera A, Moreno-Moreno M, Pina-Martinez AJ, et al. Preventing Functional Urinary Incontinence in Hip-Fractured Older Adults Through Patient Education: A Randomized Controlled Trial. Journal of Applied Gerontology.
