# Supplementary material for: The impact of urinary incontinence on falls: A systematic review and meta-analysis
Source: PLoS One. 2021 May 19;16(5):e0251711. doi: 10.1371/journal.pone.0251711 (PMC8133449; doi:10.1371/journal.pone.0251711)
Supplement: Attachment — Submitted filename: Response to Reviewers_rev.docx [file pone.0251711.s004.docx]

**PLOS ONE - Decision on Manuscript PONE-D-20-28464**

Peter F.W.M. Rosier, M.D. PhD

Academic Editor

PLOS ONE

Dear Dr. Rosier:

On behalf of all of the authors for this manuscript, we really appreciate your meticulous comments as they refine this manuscript. Regarding these comments, the reviewer’s suggestions were implemented in the revised manuscript.

The changes are summarized below **in red.**

**Reviewers' comments:**

**Reviewer #1:** In this systematic review with meta-analysis, the authors focused on the relationship between urinary incontinence and falls. Although the subject is fascinating, the study is penalized by an incomplete submission (tables 1 to 3 are missing) and the absence of subgroup analysis concerning the type of urinary incontinence (urgency vs. stress). It is therefore possible to improve this manuscript by taking into account these 2 points and those that follow:

General comment: Authors should avoid the term "elderly" or "elder" and prefer "older"

Response: Thank you for your comments. We have addressed this suggestion by changing to “older” or “older adults”.

1. Abstract: There is a difference between the abstract and the methods in the dates of literature search (October 2019 in the abstract and April 2020 in the methods)

Response: We apologized the error. As we performed the updated literature search, we changed the date (December 13 2020).

**Page 2**

**Methods**

We performed a literature search for relevant studies in databases including PubMed and Embase from inception up to December 13, 2020, using several search terms related to UI and falls. Based on the data reported in these studies, we calculated the pooled odds ratios (ORs) for falls and the corresponding 95% confidence intervals (CIs) using the Mantel–Haenszel method.

2. Methods:

a. Why did the authors choose as an eligibility criteria studies with population over 20 years? The introduction is developed around the ageing of the population and the fact that both urinary incontinence and falls are geriatric syndromes. I do know that the definition of older age varies widely among countries but 20 years old is definitely not old.

Response: As reviewer’s comments, we included the studies with population over 40 years to exclude the studies with young adults. In addition, we performed subgroup analysis of the studies with population over 65 years.

**Page 7**

**Study selection**

The inclusion criteria were as follows: 1) Population: participants aged ≥40 years; 2) Intervention: presence of UI; 3) Comparators: participants without UI; 4) Outcomes: incidence of falls; and 5) Study design: case-control studies or cohort studies.

The exclusion criteria were as follows: 1) Articles published as experimental studies, only abstracts, and non-original articles, including expert opinions or reviews; 2) Studies that enrolled young adults aged < 40 years; 3) Observational studies without a control group

b. Results of the study selection process should be included in the results and not in the methods

Response: As reviewer’s comments, we included the study selection process in the results.

**Page 9**

**Study characteristics**

In total, 1,427 studies were identified from the literature search (PubMed: 286, EMBASE: 439, Web of Science: 702). After excluding 250 duplicate studies, we reviewed the remaining studies. Next, 1,177 studies were excluded during primary screening. After reviewing the texts of 107 articles, we excluded 73 additional studies, resulting in the inclusion of 34 articles [6-10, 13-40]. In addition, we could find four eligible studies from previous review [41]. Finally, a total of 38 studies with 230,129 participants were included in this meta-analysis (Fig. 1).

c. Data extraction: there is no information on urinary incontinence, its type or severity.

Response: As reviewer’s comments, we conducted additional subgroup analysis according to the type of UI. However, because of lack of data, we could not analyze the effect of the severity of UI on falls.

**Page 18**

In a subgroup analysis according to the type of UI, A significant association between UI and falls was observed in patients with urgency UI (OR, 1.76; 95% CI, 1.15-1.70) [6, 7, 9, 17, 29] and those with stress UI (OR, 1.73; 95% CI, 1.39-2.15)[6, 7, 9, 17, 29].

d. Quality assessment. This section should be renamed as: risk of bias assessment. Unfortunately, there is no validation study of the Newcastle Ottawa tool, and no validation of the cut-off used to assess the risk of bias (what is the reference for this cut-off?) Could the authors discuss the choice of this tool and use a more appropriate one?

Response: We thanks for the reviewer’s careful comments. Recently, ROBINS-I is the preferred tool to be used in Cochrane Reviews for non-randomized studies of interventions. However, ROBINS-I has limitation on this meta-analysis because our meta-analysis did not use the intervention studies. For observational studies, the Newcastle-Ottawa Scale might be an alternative option. As reviewer’s comments, Newcastle-Ottawa Scale also had several limitations. Therefore, we used ROBINS-E, a modified form of ROBINS-I for exposure studies.

Reference

https://methods.cochrane.org/methods-cochrane/robins-i-tool

Sterne J.A., Hernán M.A., Reeves B.C., Savović J., Berkman N.D., Viswanathan M., Henry D., Altman D.G., Ansari M.T., Boutron I. ROBINS-I: A tool for assessing risk of bias in non-randomised studies of interventions. BMJ. 2016;355:i4919. doi: 10.1136/bmj.i4919

Morgan RL, Thayer KA, Santesso N, Holloway AC, Blain R, Eftim SE, Goldstone AE, Ross P, Ansari M, Akl EA, Filippini T, Hansell A, Meerpohl JJ, Mustafa RA, Verbeek J, Vinceti M, Whaley P, Schünemann HJ; GRADE Working Group. A risk of bias instrument for non-randomized studies of exposures: A users' guide to its application in the context of GRADE. Environ Int. 2019 Jan;122:168-184. doi: 10.1016/j.envint.2018.11.004. Epub 2018 Nov 22. PMID: 30473382

**Page 8**

**Risk of bias assessment**

We used the Risk Of Bias In Non-randomized Studies - of Exposures (ROBINS-E), a modified form of ROBINS - of Interventions (ROBINS-I) to assess the methodological quality of the included studies[11, 12]. Discrepancies were resolved by discussion with a third investigator (J.M.Y).

**Figure 2**

e. Why the authors did not register their systematic review and meta-analysis protocol? It is well known that the absence of a published protocol can lead to bias (10.1001/jama.287.21.2831).

Response: We thanks for the reviewer’s careful comments. The study protocol was registered in PROSPERO (CRD42021225038).

**Page 7**

The study protocol was registered in PROSPERO (CRD42021225038).

f. Regarding the search strategy, there is no full electronic search strategy available as it is stated in the PRISMA checklist. Authors should provide it as a supplementary data.

Response: As reviewer’s comments, we provided the electronic search strategy in the supplementary table 2.

| **Database** | **Search term (inception to December 13, 2020)** | **Number of studies** |
| --- | --- | --- |
| **PubMed**  **No filters activated** | **(Urinary incontinence) AND (fall)** | **286** |
| **EMBASE**  **No filters activated** | **‘Urinary incontinence’ AND ‘fall’** | **439** |
| **Web of Science**  **Core Collection** | **(Urinary incontinence) AND (fall)** | **702** |

3. Results:

a. The results section is uncomplete since tables 1 to 3 are missing (problem during the submission process?). It is difficult to judge this work without these tables, and I cannot make much more comments without these tables.

Response: We apologized the mistakes. We attached Tables.

b. It seems that there is a problem with the figure 3 (“omitting” in front of each study name). Did the authors proofread their manuscript?

Response: Figure 3 (revised supplementary figure 1B) is about sensitivity analysis. In sensitivity analysis, we omitted one study to estimate the effect of each study. The results of each row mean the OR of meta-analysis after omitting each study. We modified the figure more clearly.

Figure 4

c. The study by Peeters et al in 2019 was included 4 times in the meta-analysis. Could the authors provide an explanation? Are they sure that it was not conducted in the same population?

Response: The study by Peeters used four large cohort data which are collected independently. They used the data form population-based cohort studies including the Australian Longitudinal Study on Women’s Health (Australia, n = 10,641, 51–58 years in 2004), the Irish Longitudinal Study on Ageing (Ireland, n = 4663, 40–64 years in 2010), the Longitudinal Ageing Study Amsterdam (Netherlands, n = 862, 55–64 years in 2012–13) and the MRC National Survey of Health and Development (Great Britain, n = 2987, 53 years in 1999).

4. Discussion:

Since we do not have any tables, it is difficult to understand why the authors did not discuss a critical point. I think that the association between UI and falls is rather known and admitted, but the remaining question is “is there any causality between UI and falls”? As the authors said, UI and falls are probably more related to “frailty” and the significant association that many studies highlight may not exist as such.

o Do we have any prospective data to answer this question?

Response: As the reviewer pointed out, we have searched several literatures and revised the discussion section adding the latest data.

**Page 21**

There is a well-recognized association between falls and lower urinary tract symptoms (LUTS) in the older adults [6, 7, 41, 46, 47]. Older people with urgency or urgency UI are significantly more likely to fall than age-matched controls, with ORs for falls ranging between 1.5 and 2.3[5, 41, 48, 49]. However, the reason for this association is not understood and has not been thoroughly studied [5].

In a recent systemic review on the association between falls and LUTS conducted by Noguchi et al., none of the studies identified have investigated the potential causes of these associations. In addition, the categorization of UI and degree of accounting for confounding variables were inconsistent across the studies[50]. Although the data identified were suitable only for qualitative synthesis, UI and storage symptoms among LUTS are consistently reported to have a weak to moderate association with falls[5, 50].

o Do we have some biological hypothesis to support this? (rather not, the authors started to discuss this point but not enough to my opinion, they forgot to include in there discussion the studies published by Dumoulin et al. on gait and urge to void, by Fritel et al. on urinary incontinence subtypes and mobility impairment, etc. )

Response: We fully agree with the reviewer. We have reviewed several articles, including the references the reviewer suggested. As the reviewer pointed out, we have added biological hypotheses in the discussion section for supporting causality between UI and falls with references (Dumoulin et al. and Fritel et al.).

**Page 21**

Few studies have investigated the relationship between UI and falls [41], and the causality between UI and falls remains unexplained[5]. However, one hypothesis is that a strong desire to void could change gait parameters and thus increase the risk of falls[44]. The reduced velocity and stride width during strong desire to void conditions (i.e., urgency) in the UI group could explain their higher fall rate[44]. The other hypothesis is that women with impaired mobility probably take longer time to reach the toilet; hence, if there is a high degree of urgency, then impaired mobility can increase the risk of UI [45]. Therefore, the causality between UI and falls could probably be explained by that strong desire to void and physical impairments in mobility and balance may give rise to increase the risk of falls [44, 45].

o Were the studies adjusted on potential confounders? (neurological pathologies? Comorbidities? Polypharmacy?)

Response: Thank you for your comments. As the reviewer pointed out, neurological status, comorbidity conditions and polypharmacy may act as potential confounders. However, there were no studies that were adjusted on neurologic diseases and polypharmacy. In addition, we have classified the studies that were not adjusted on age, sex and comorbidity as critical risk of bias.

o Was the association different if stress or urgency urinary incontinence were considered?

Response: We thanks for the reviewer’s comment. As reviewer’s comments, we have conducted updated literature search and additional subgroup analysis according to the type of UI. According to the type of UI, a significant association between UI and falls was observed in patients with urgency UI (OR, 1.76; 95% CI, 1.15 to 1.70) and those with stress UI (OR, 1.73; 95% CI, 1.39 to 2.15). We have updated additional results in discussion section.

**Page 18**

In a subgroup analysis according to the type of UI, A significant association between UI and falls was observed in patients with urgency UI (OR, 1.76; 95% CI, 1.15-1.70) [6, 7, 9, 17, 29] and those with stress UI (OR, 1.73; 95% CI, 1.39-2.15)[6, 7, 9, 17, 29].

**Page 20**

We conducted another subgroup analysis according to the type of UI. A previous review highlighted a predominant association of falls with urgency UI, rather than with other types of UI[41]. This association is attributed to the urgent need to use the toilet and the anxiety associated with a failure to reach the toilet. Several studies have shown that behavioral changes induced by UI can affect the likelihood of falls[42, 43]. Our analysis also showed a higher risk of falls in urgency UI than in stress UI.

**Reviewer #2:** I want to congratulate the authors for the idea and development of this manuscript. The paper adds valuable information about the link of falls and UI in terms of higher level of evidence after this systematic review.

I suggest some comments in order to improve quality and understanding of the manuscript:

1. Figure 4 can be omitted, from my view it does not add important information for the reader

Response: To reduce the number of figure, we combined funnel plot and sensitivity analysis in one figure (Figure 4)

2. Please describe acronyms TE/seTE in figure 2.

Response: As reviewer’s comments, we modified Figure 2 (revised table 3).

3. There is a significative statistical heterogeneity in the studies included. This is reflected in the results section, but it is a limitation of the study and need to be commented in the discussion. Probably this point and the quality of the study designs and the reporting scope of the original articles is the main limitation of this meta-analysis.

Response: Thank you for your comment as it certainly refines this manuscript. We have addressed the statistical heterogeneity as a limitation of the study in the discussion section as reviewer has suggested.

**Page 23**

Despite these strengths, our study was limited largely by the included studies, particularly the significant heterogeneity, the quality of the study designs, and the reporting scope of the original articles. However, when studies with critical risk of bias were excluded, significant results were observed. In addition, publication bias was not observed, and the results were not changed by specific studies in sensitivity analysis.

4. Some studies have shown association between type of UI (specially UUI) and severity (more number of UUI episodes per week). The authors don’t clarify why they don’t include this subgroup analysis. It would be very interesting to have this information in this kind of systematic review.

Response: We thanks for the reviewer’s careful comments. Although several studies reported the type of UI, the number of studies were too small to conduct meta-analysis. However, as we performed updated literature search, we could find enough studies to perform subgroup analysis. We added the results to table 2.

**Page 18**

In a subgroup analysis according to the type of UI, A significant association between UI and falls was observed in patients with urgency UI (OR, 1.76; 95% CI, 1.15-1.70) [6, 7, 9, 17, 29] and those with stress UI (OR, 1.73; 95% CI, 1.39-2.15)[6, 7, 9, 17, 29].

5. There is a previous systematic review on LUTS and falls that the authors has not mentioned [Noguchi, N., et al., A systematic review of the association between lower urinary tract symptoms and falls, injuries, and fractures in community-dwelling older men. Aging Male, 2016: p. 1-7.]. In this SR none of the identified studies examined potential causes for these associations; the categorisation of continence or not and degree of accounting for confounding variables was inconsistent across the included studies. This point should be discussed and compared with results with your SR.

Response: As the reviewer pointed out, we reviewed Noguchi N et al. A systematic review of the association between lower urinary tract symptoms and falls, injuries, and fractures in community-dwelling older men (Aging Male. 2016;19:168-174). In addition, we have compared with our results in the discussion section according to the reviewer's comments.

**Page 21**

There is a well-recognized association between falls and lower urinary tract symptoms (LUTS) in the older adults [6, 7, 41, 46, 47]. Older people with urgency or urgency UI are significantly more likely to fall than age-matched controls, with ORs for falls ranging between 1.5 and 2.3[5, 41, 48, 49]. However, the reason for this association is not understood and has not been thoroughly studied [5].

In a recent systemic review on the association between falls and LUTS conducted by Noguchi et al., none of the studies identified have investigated the potential causes of these associations. In addition, the categorization of UI and degree of accounting for confounding variables were inconsistent across the studies[50]. Although the data identified were suitable only for qualitative synthesis, UI and storage symptoms among LUTS are consistently reported to have a weak to moderate association with falls[5, 50].

6. From the point of view of the reader is not clear the categorization in the definition of falls (≥1, ≥2, ≥2 recurrent falls). It would be very helpful to provide a definition of falls (within which time lapse?, what is the definition of recurrent falls?).

Response: Thank you for pointing this out. We conducted additional subgroup analysis to the definition of falls. In this sub analysis, we defined falls as occurred at least once in the past 1 year (≥1 fall event) and recurrent falls as when those occurred twice or more in the past 1 year (≥2 fall events). This categorization has been done in most studies.

Thank you for the reviewers' warm remarks. Authors tried to revise the manuscript following their opinions as far as we can. We hope the revised manuscript will better meet the requirements of your journal for publication. We thank the editor and the reviews of PLOS ONE once again for the constructive review of our paper.

Sincerely yours,

Sung Tae Cho, MD, Ph.D.

Professor, Dept. of Urology,

Kangnam Sacred Heart Hospital

Hallym University College of Medicine
