# Supplementary material for: The impact of urinary incontinence on falls: A systematic review and meta-analysis
Source: PLoS One. 2021 May 19;16(5):e0251711. doi: 10.1371/journal.pone.0251711 (PMC8133449; doi:10.1371/journal.pone.0251711)
Supplement: Attachment — Submitted filename: Response to Reviewers_0425.docx [file pone.0251711.s005.docx]

**PLOS ONE - Decision on Manuscript PONE-D-20-28464R1**

Peter F.W.M. Rosier, M.D. PhD

Academic Editor

PLOS ONE

Dear Dr. Rosier:

We are pleased to re-submit our revised manuscript to the PLOS ONE. We express our sincere appreciation for your thoughtful comments. Following your suggestions, we describe answers about the issues raised by reviewers. In addition, we designate any changes by highlighting with **red color** in the revised manuscript.

**Reviewers' comments:**

**Reviewer #1:** Thank you to the authors for taking our remarks into account and for modifying the manuscript accordingly, which has been considerably improved. Please find additional comments.

**Methods**

- It is still not clear why this age (≥40 years) was chosen in a study about an essentially geriatric problem. Authors must explain it. Why did they not choose ≥ 60 years old? I only found 2 studies in the results with a possible mean age under 60 (Giraldo Rodriguez L et al. 2019 [29] and Abbs E et al. 2020 [31]). This remark does not mean that I want the authors to remove these studies, but I really want to know the reason behind this selection criteria.

Response: Thank you for careful comments. Considering the heterogeneous characteristics between young adults and older adults, we excluded the studies with young adults to remove their confounding effect on the results. However, considering several cohort studies often included middle aged people in their baseline survey, we thought that tight age criterion might miss large population based cohort studies such as Giraldo‐Rodríguez et al. Therefore, we chose the broad criterion (≥40 years) to screen as many studies as possible. In addition, we thought the heterogeneous effect of middle aged people could be solved by subgroup analysis stratified by age and conducted a subgroup analysis with studies in older adults (≥65 years). Nevertheless, we agreed the reviewer’ comment that this criterion could not explain enough of the aim of our study. Therefore, we modified this criterion as follow:

1) population: studies with participants aged ≥ 50 years or mean age ≥ 60 years

- Study selection: rather use “exposure” than “intervention”

Response: As reviewer’s comment, we revised the manuscript.

**Results**

- In table 1, there are some missing informations:

* population characteristics: sex ratio (Tinetti, Johansson etc.), age (de Rekeneire, Huang, etc.) or both (Kim) are missing for some of the studies. Please fill in the missing informations.

* exposition: there is no information on the definition/type/prevalence of UI. Please fill in the missing informations.

* I would merge the 2 columns “definition of falls” and “no. of participants with falls” so that you can add a “UI” column.

* for adjusted OR, I would add, as a footnote under the table, the variables of adjustment.

* when OR is not available, please give the prevalence in the two groups.

* footnotes: missing explanation of the abbreviations: ALSWH, LASA, NSHD, TILDA, UI, CI

* For the study by Stenhagen et al., were the OR calculated on the 6 years or 3 years data? Please indicate the number of participants according to the OR calculation

**Response:** We agree with the reviewer. For all studies, we have checked again to find if there was any information missing. Then, we have added the missing data and modified the table 1. as the reviewer pointed out.

- Risk of bias assessment

*Figure 2 risk of bias: The authors forgot to adapt the ROBINS-I template to the ROBINS-E tool. For example, D3 bias in classification of interventions is not applicable to the ROBINS-E tool and should be replace by “Bias in classification of exposures”. Please check every single item to be in accordance with the ROBINS-E tool.

Response: As reviewer’s comment, we revised figure 2.

*In the manuscript, I think you should explain a little bit more the risk of bias. At least write the major source of bias (bias due to confounding) and highlight the studies who correctly address confounding factors in the relationship between UI and falls.

Thank you for careful comments. We explained more details about the risk of bias in manuscript.

- Figures 3 et 4 B

The “favours A/favours B” footnote does not work here since authors assess the effect of an exposure and not an intervention. Please modify to something more adequate to this analysis

Response: As reviewer’s comment, we revised figure 3 and 4 as follow:

favours A/favours B 🡪 Decreased risk of falls/Increased risk of falls

**Discussion**

The authors have made some changes regarding the hypotheses to explain of the association between urge UI and falls. But the authors did not make any hypotheses to explain the association between stress UI and falls. To my opinion it is important, and it is one of the most interesting findings of this meta-analysis. Clinicians will remember that UI of any type is a factor associated with falls. But authors must make some hypotheses.

**Response:** We agree with the reviewer that the hypothesis of stress UI is different to those of urgency UI. Thus, we have added the sentence in the discussion section as the reviewer pointed out (page 24, lines 13-19).

Is UI just a marker of poor health? Or is UI a real causal factor? Here authors should discuss a little bit more the studies that correctly address confounding factors, especially the ones that could lead to both UI and falls (for example multimorbidity, polypharmacy, neurological diseases, sarcopenia, etc.).

**Response:** Thank you for your comments. Thus, we have performed the updated literature search and added the sentence about confounding factors such as multimorbidity, polypharmacy, neurological diseases and sarcopenia in the discussion section as the reviewer pointed out (page 25, lines 12-22).

We hope this revised work is impressive to your editor, reviewers, and readers. We thank the editor and the reviews of PLOS ONE once again for the warm review of our paper and look forward to hearing from you.

Sincerely yours,

Sung Tae Cho, MD, Ph.D.

Professor, Dept. of Urology,

Kangnam Sacred Heart Hospital

Hallym University College of Medicine
